# The genus *Haplopappus*: botany, phytochemistry, traditional uses, and pharmacological properties

**DOI:** 10.3389/fphar.2024.1490243

**Published:** 2024-10-21

**Authors:** Christina Mitsi, Javier Echeverría

**Affiliations:** Departamento de Ciencias del Ambiente, Facultad de Química y Biología, Universidad de Santiago de Chile, Santiago, Chile

**Keywords:** *Haplopappus* genus, ethnobotany, traditional uses, phytochemistry, pharmacology

## Abstract

**Background:**

The genus *Haplopappus* Cass. [Asteraceae] comprises a large number of species distributed mainly in Chile and with various traditional medicinal uses.

**Purpose:**

The present review addresses the botany, traditional uses, chemistry, biological and pharmacological activities of the genus, aiming to further potentiate the associated research and applications.

**Study design and Methods:**

Literature data on the chemistry and bioactivity of the genus Haplopappus were mainly retrieved from digital databases such as SciFinder^®^, PubMed^®^, and Google Scholar^®^, as well as from the scientific journal publishers’ platforms linked with these databases.

**Results and discussion:**

Although the majority of the botanical taxa of the genus *Haplopappus* has been understudied, available information is promising regarding its phytochemistry and bioactivity. A total of more than 400 compounds are present in different *Haplopappus* species, mostly terpenoids and phenolic compounds. Scientific literature supports various health promoting effects of *Haplopappus* extracts and isolated compounds, principally their effect against human pathogenic bacteria and their high antioxidant capacity. The existing limitations highlighted hereby are mainly associated to the lack of modern investigation regarding a wider number of *Haplopappus* species and chemical compounds, as well as to the absence of *in vivo* bioactivity results and clinical trials.

**Conclusion:**

Scientific literature supports the ethnopharmacological, phytochemical and bioactive potential of the genus *Haplopappus*, however the aforementioned limitations need to be addressed in order to further promote and broaden both scientific research and future applications and uses.

## 1 Introduction

*Haplopappus* Cass. (Asteraceae (Compositae) - Astereae - Machaerantherinae), is a strictly endemic botanical genus of southern South America, distributed in Chile, with some species also present in Argentina (Klingenberg, 2007). The vernacular name *‘bailahuén’* (*‘baylahuén’* or *‘vaila-huen’*) has been mainly attributed to the species *Haplopappus baylahuen* Remy, although the other species of the genus are commonly referred to using the same name (Vogel et al., 2007).

The different species of the genus *Haplopappus*, although used without differentiation in terms of botanical taxa, are of high ethnopharmacological importance and form part of the longstanding traditional medicines of the Andean peoples. In Chile, where the genus is mainly distributed, its species have been widely used in all territory, from the Aymara communities in the north to Mapuche communities in the south, and in big cities by different social groups (Hoffmann et al., 1992). *Bailahuén* is used at the prevention and/or treatment of various human and animal pathologies, mainly -but not exclusively-associated to gastrointestinal ailments and wound healing (Muñoz et al., 1981; de Mösbach, 1992; Hoffmann et al., 1992). Alongside its traditional use, *H. baylahuen* is also included in the German Homeopathic Pharmacopeia as a herbal medicine against fatigue and low blood pressure, although its use is considered limited (Arzneibuch, 2006; Vogel et al., 2007).

Regarding its commercialization, it is reported that its production in Chile is exclusively based on the collection of plant material in the wild, which, in most cases, is realized by non-trained individuals (Vogel et al., 2007). Furthermore, in the same study it is highlighted that the 80% of *bailahuén* commercial samples correspond to *Haplopappus multifolius*, probably due to the fact that this species is distributed in the Metropolitan Region of Santiago, where the companies that commercialize the plant material at a national and international level are also located. The over-exploitation of *H. multifolius*, along with inadequate collection practices, have led to the species being recently included in The IUCN Red List of Threatened Species as Near Threatened (Plummer, 2022).

In this context, despite its high botanical diversity and the rich ethnopharmacological background of the genus *Haplopappus*, both scientific investigation and commercial use is often limited to a few botanical taxa, while in many cases the traditional knowledge associated with the genus is not taken into consideration, thus hindering unravelling the full phytochemical and bioactive potential of the genus.

Thus, the present article aims to present a comprehensive review of the current state of knowledge regarding the botany, traditional uses, chemistry, biological and pharmacological activities of the genus *Haplopappus* in an attempt to underline its phytochemical uniqueness, elucidate its bioactive potential, and highlight future research opportunities.

## 2 Methods

**FIGURE 1:**
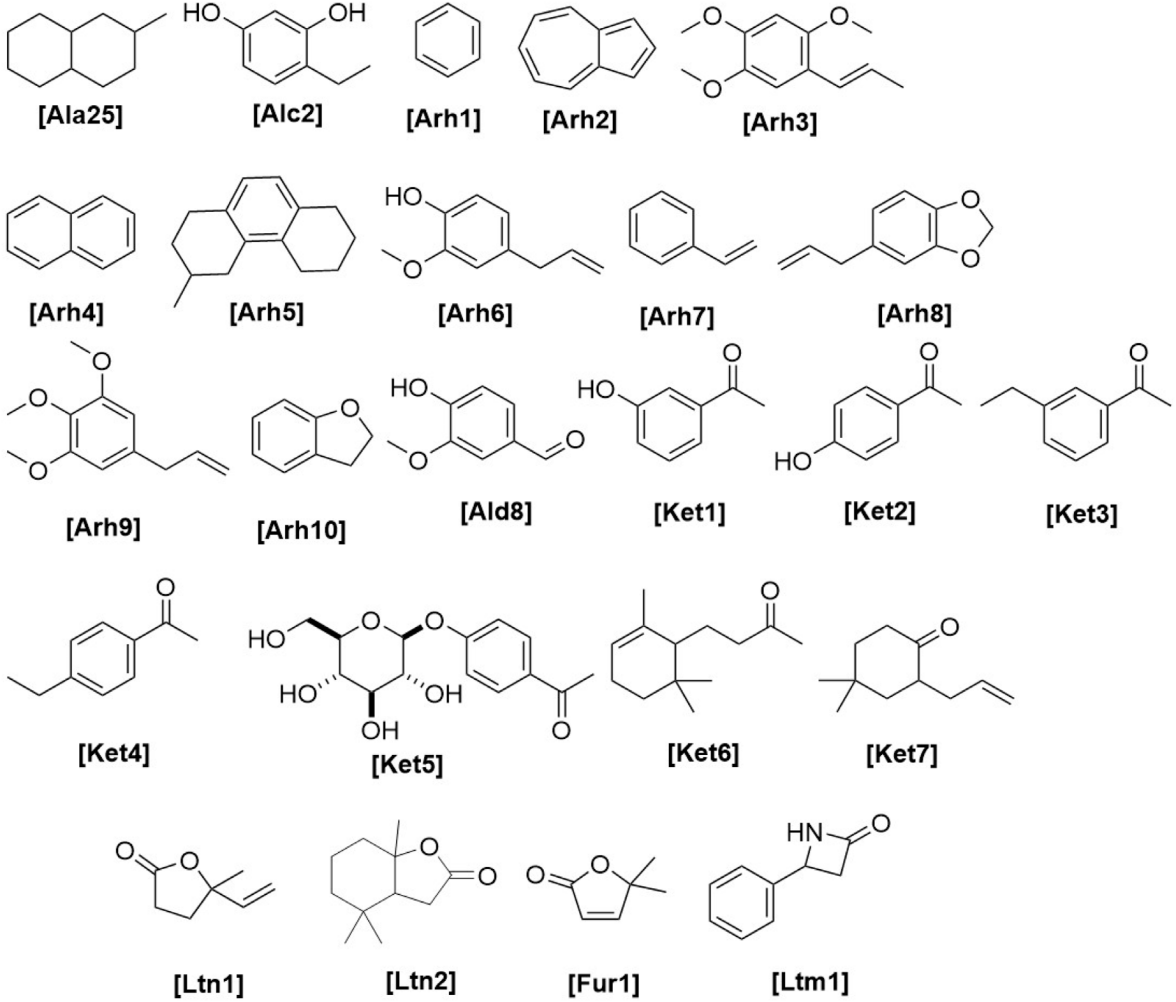
Chemical structures of miscellaneous compounds identified from *Haplopappus* species.

**FIGURE 2:**
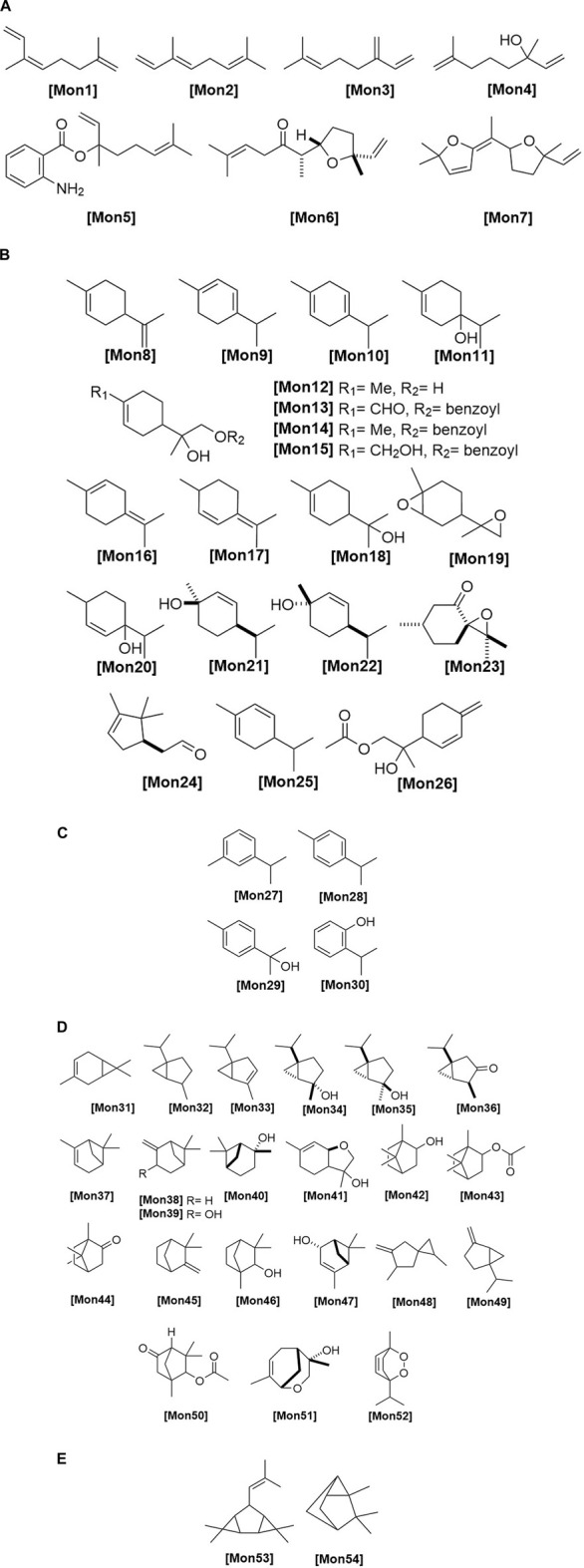
**(A)** Chemical structures of acyclic monoterpenes identified from *Haplopappus* species. **(B)** Chemical structures of monocyclic monoterpenes identified from *Haplopappus* species. **(C)** Chemical structures of aromatic monocyclic monoterpenes identified from *Haplopappus* species. **(D)** Chemical structures of bicyclic monoterpenes identified from *Haplopappus* species. **(E)** Chemical structures of tricyclic monoterpenes identified from *Haplopappus* species.,

**FIGURE 3:**
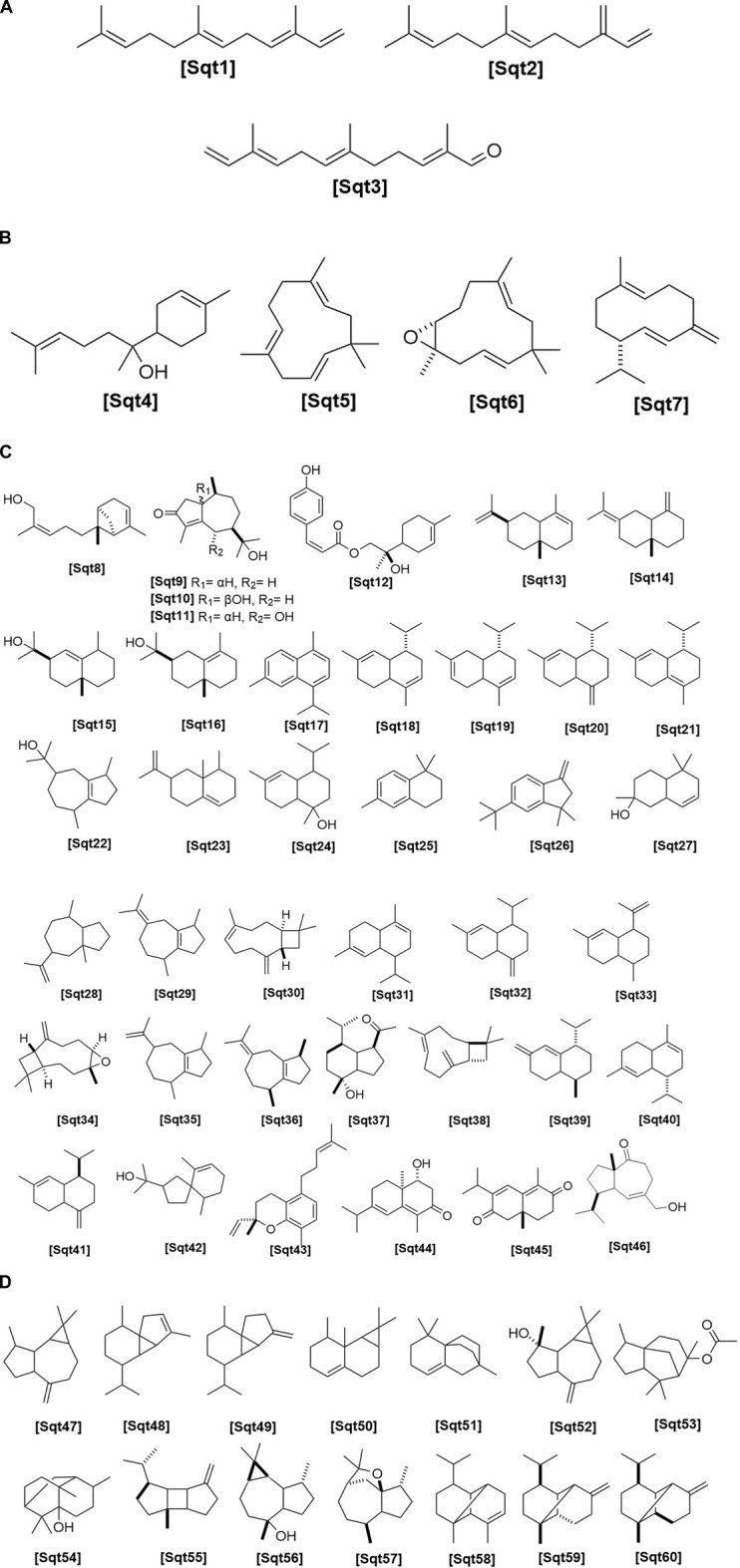
**(A)** Chemical structures of acyclic sesquiterpenes identified from *Haplopappus* species. **(B)** Chemical structures of monocyclic sesquiterpenes identified from *Haplopappus* species. **(C)** Chemical structures of bicyclic sesquiterpenes identified from *Haplopappus* species. **(D)** Chemical structures of tricyclic sesquiterpenes identified from *Haplopappus* species.,

**FIGURE 4:**
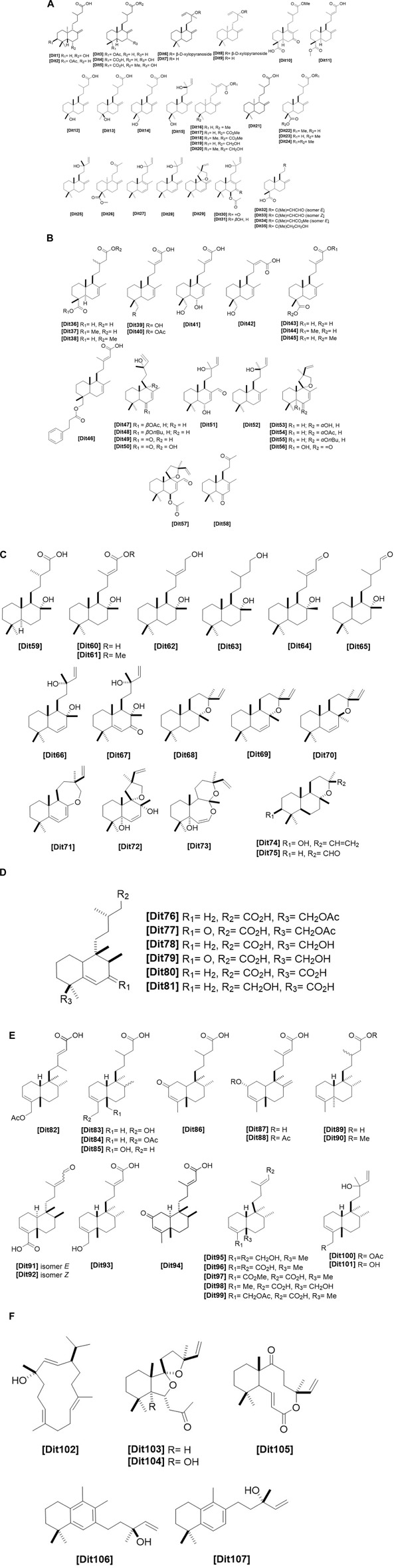
**(A)** Chemical structures of diterpenes labdane-type1 identified from *Haplopappus* species. **(B)** Chemical structures of diterpenes labdane-type2 identified from *Haplopappus* species. **(C)** Chemical structures of diterpenes labdane-type3 identified from *Haplopappus* species. **(D)** Chemical structures of diterpenes friedolabdane-type identified from *Haplopappus* species. **(E)** Chemical structures of diterpenes clerodane-type identified from *Haplopappus* species. **(F)** Chemical structures of miscellaneous diterpenes identified from *Haplopappus* species., ,

**FIGURE 5:**
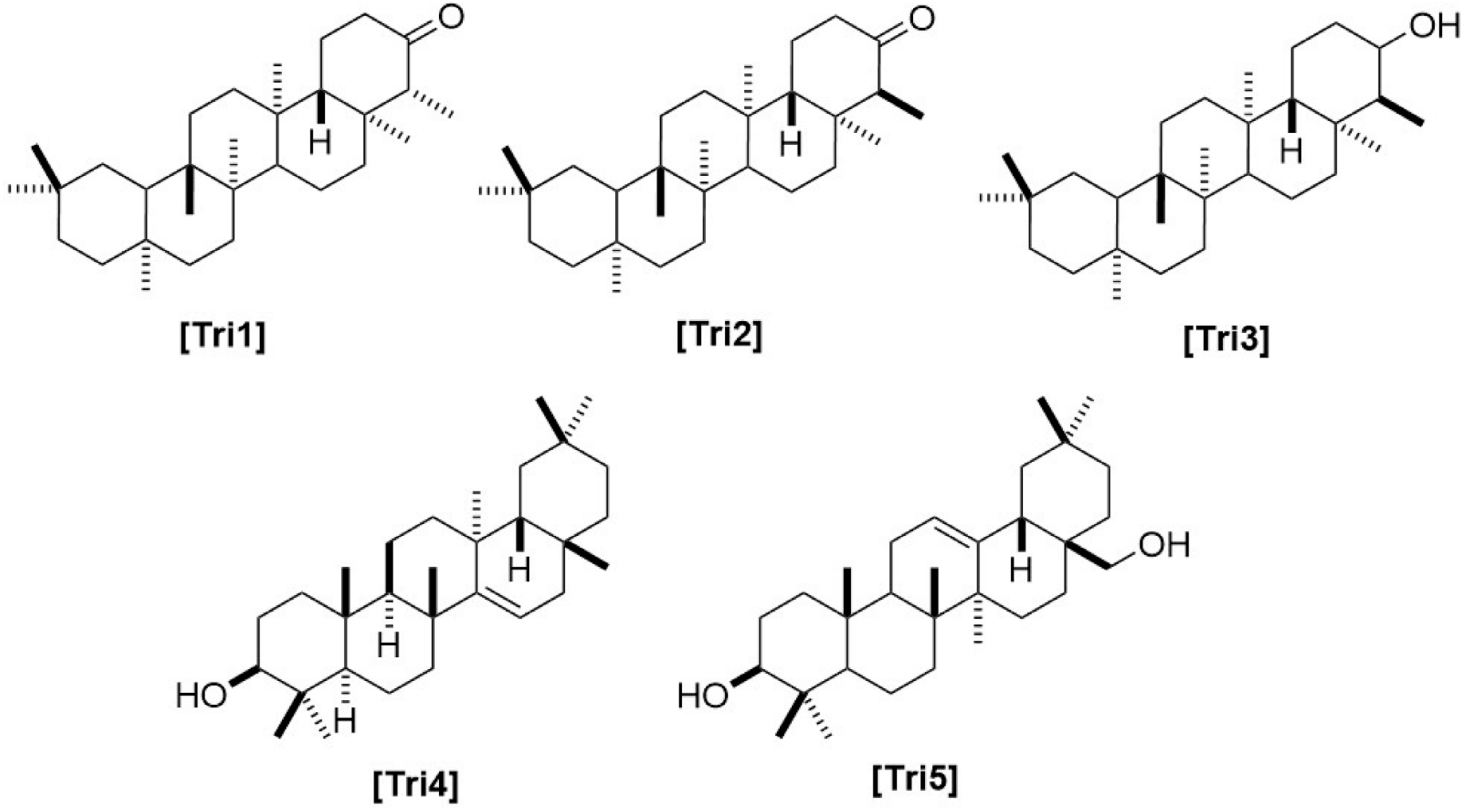
Chemical structures of triterpenes and triterpenoids identified from *Haplopappus* species.

**FIGURE 6:**
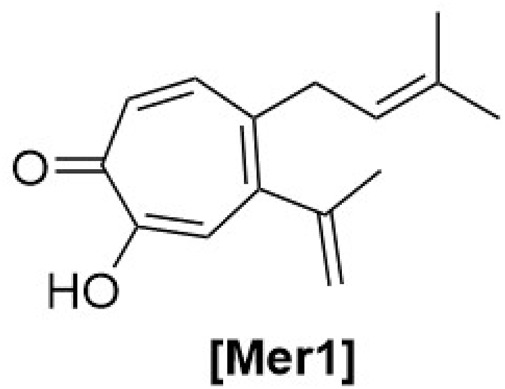
Chemical structures of meroterpenes identified from *Haplopappus* species.

**FIGURE 7:**
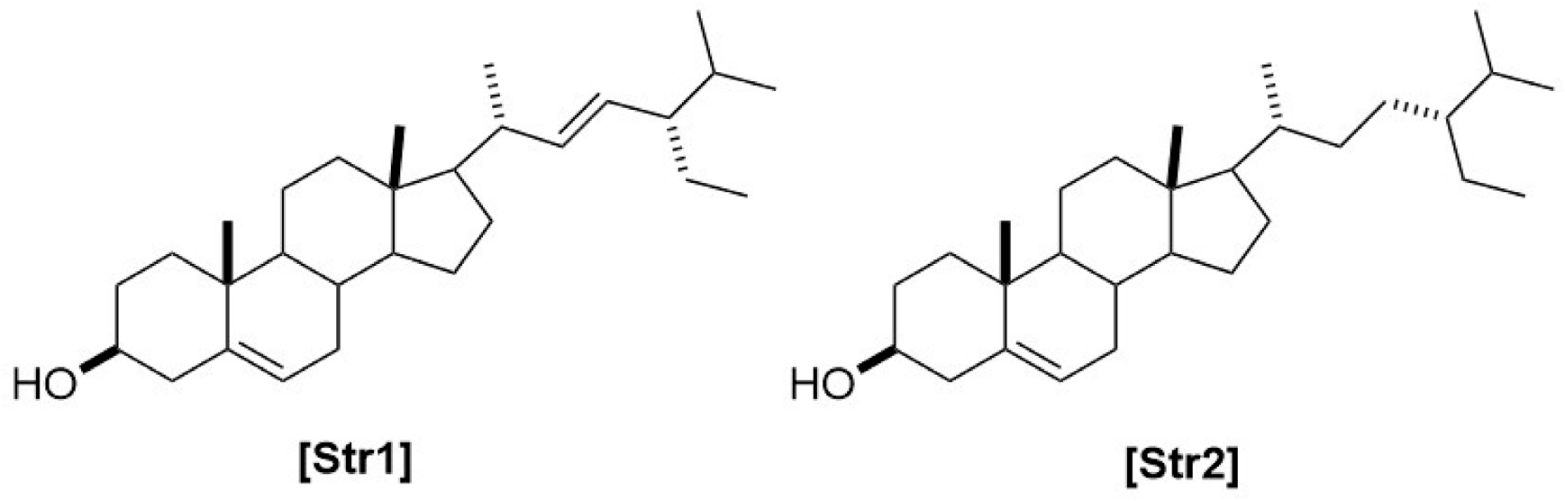
Chemical structures of steroids identified from *Haplopappus* species.

**FIGURE 8:**
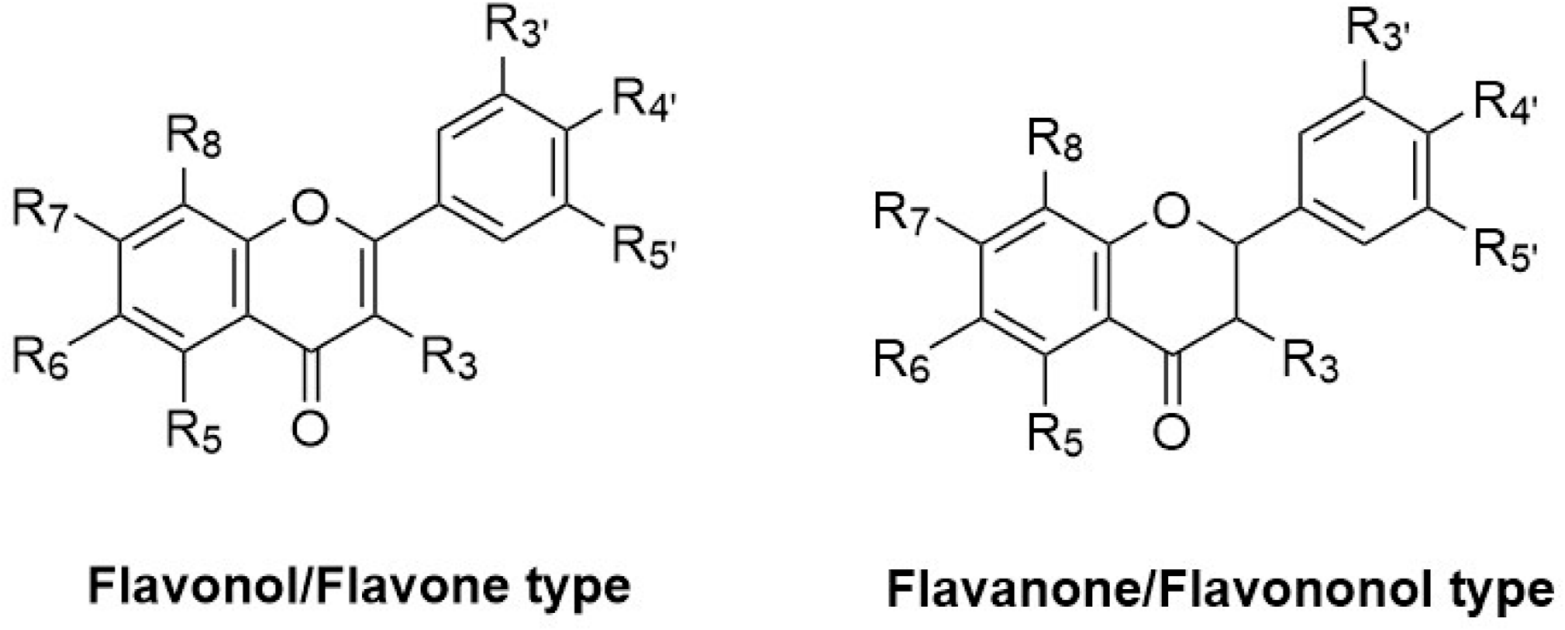
Chemical structures and substitution patterns of flavonoids identified from *Haplopappus* species.

**FIGURE 9:**
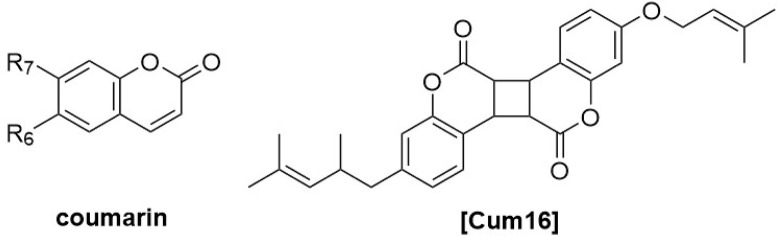
Chemical structures and substitution patterns of coumarins identified from *Haplopappu*s species.

**FIGURE 10:**
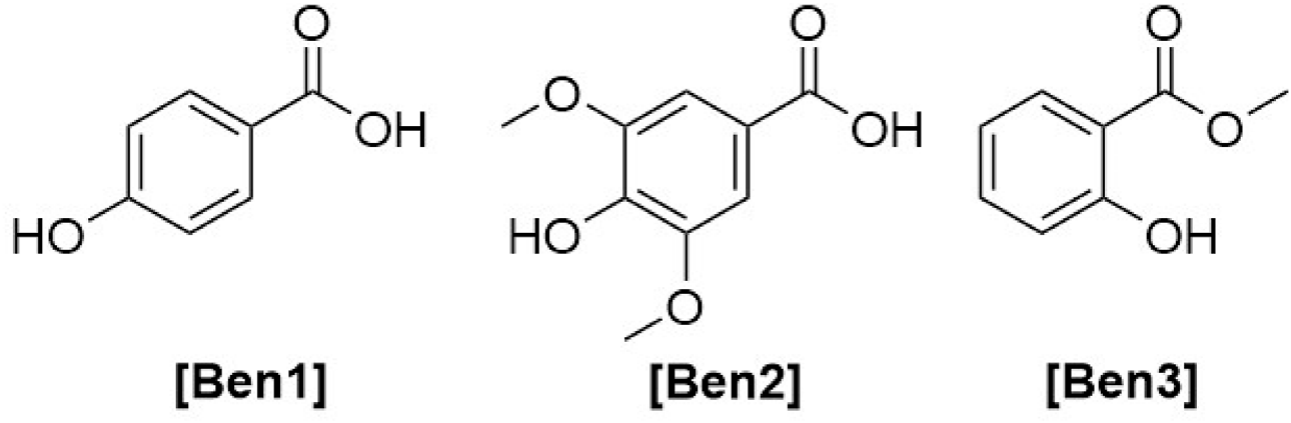
Chemical structures of benzoic acid derivatives identified from *Haplopappus* species.

**FIGURE 11:**
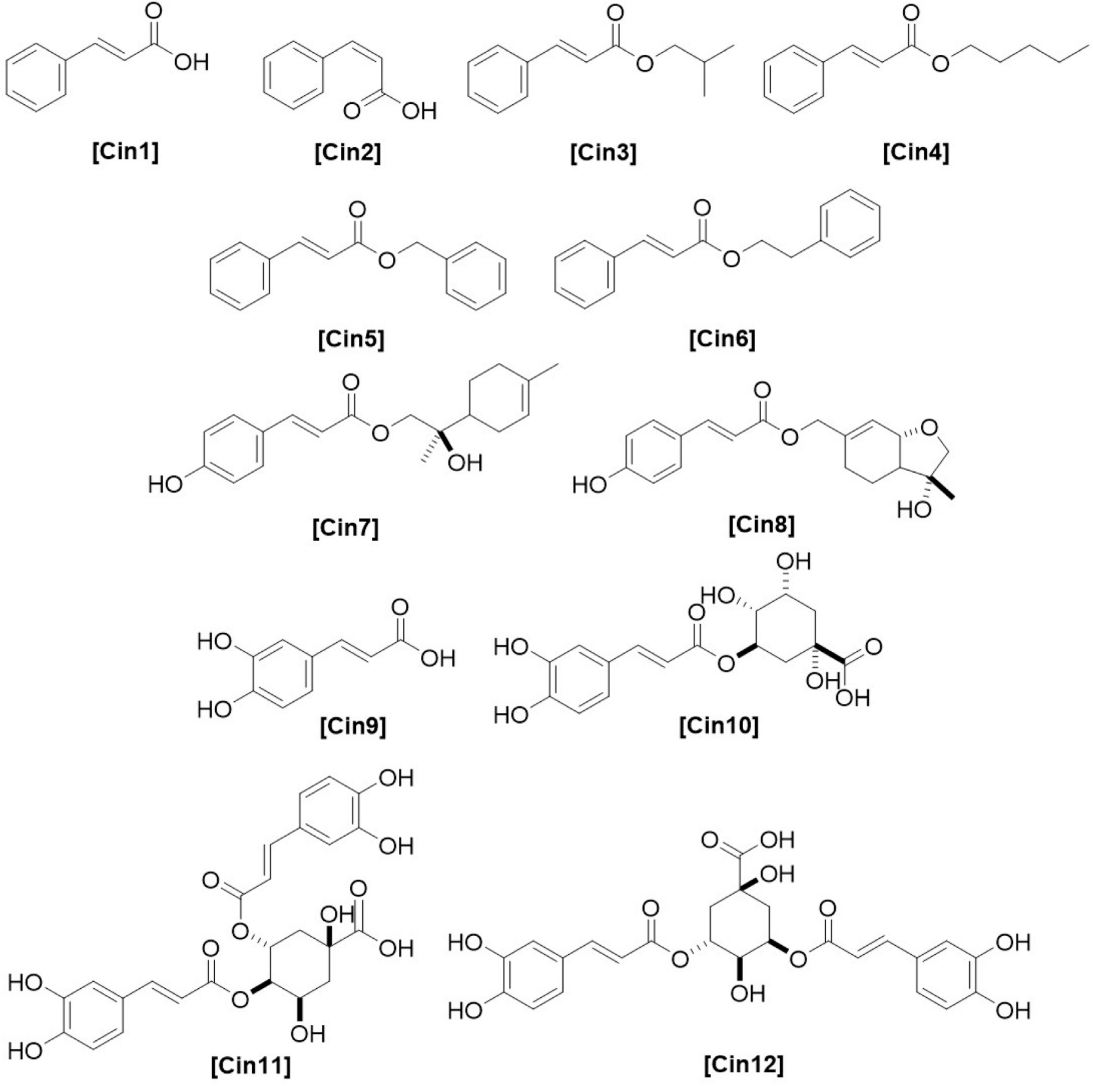
Chemical structures of cinnamic acid derivatives identified from *Haplopappus* species.

Literature data on the chemistry and bioactivity of the genus *Haplopappus* were mainly retrieved from digital databases such as SciFinder^®^, PubMed^®^, and Google Scholar^®^, as well as from the scientific journal publishers’ platforms linked with these databases. The search strategy included the scientific name of the genus, excluding the species presently classified in other genera, i.e., *Ericameria* Nutt., *Grindelia* Willd., *Gundlachia* A.Gray, *Isocoma* Nutt., *Notopappus* L. Klingberg (Klingenberg, 2007; POWO, 2024). All publications in peer-reviewed journals until May 2024 were considered. The chemical compounds present in the raw materials were classified according to their pathway and superclass (Supplementary Table S1; Figures 1–11) using the NPClassifier tool (Kim et al., 2021).

## 3 Botany and distribution

The genus *Haplopappus* Cass. (Asteraceae - Astereae - Machaerantherinae) is a strictly endemic genus of South America and its species are mainly distributed in Chile and, to a lesser extent, Argentina (Klingenberg, 2007; Rodriguez et al., 2018; Zuloaga et al., 2019; García et al., 2024).

According to the latest taxonomic studies of the genus and after the separation of numerous, mainly North American, species that formed the genus *Notopappus* L. Klingenberg, the genus *Haplopappus* consists of 70 specific and intraspecific taxa (Table 1) and is subdivided into three subgenera (*Haplopappus* subgen. *Haplopappus*, *H.* subgen. *Grindelioidae* Klingenberg, and *H.* subgen. *Baylahuen* Klingenberg) and five sections: *Haplopappus* sect. *Haplopappus*, *H.* sect. *Gymnocoma* Nuttall, *H.* sect. *Grindelioidae* Klingenberg, *H.* sect. *Chromochaeta* Candolle, and *H.* sect. *Leiachaenium* Candolle (Klingenberg, 2007; Garcia et al., 2018; García et al., 2024). *Haplopappus* taxonomy is mainly based on morphological traits, due to the limited phylogenetic data available up to date (García et al., 2024). In general, *Haplopappus* species are shrubs or subshrubs, with aerial parts that bear glandular trichomes, usually yellow florets, and numerous pappus bristles (Klingenberg, 2007; García et al., 2024).

**TABLE 1:** Scientific names and distribution of reported *Haplopappus* species (Klingenberg, 2007; Garcia et al., 2018; García et al., 2024).

No.	*Haplopappus* species	Synonyms	Distribution^a^
1	*H. angustifolius* (DC.) Reiche subsp. *angustifolius*	*Aster atenes* Kuntze, *Aster sternbergii* Kuntze, *H. durus* Reiche, *Pyrrocoma angustifolia* DC., *Pyrrocoma rigida* Phil	Chile (Atacama, Coquimbo)
2	*H. angustifolius* (DC.) Reiche subsp. *saxatilis* (Remy) Klingenb	*Aster saxatilis* (Remy) Kuntze, *Haplodiscus sphacelatus* Phil., *H. saxatilis* (Remy) Reiche, *H. sphacelatus* (Phil.) Reiche, *Pyrrocoma saxatilis* Remy	Chile (Coquimbo, Metropolitan of Santiago, Maule)
3	*H. anthylloides* Meyen & Walp	*Aster anthylloides* (Meyen & Walp.) Kuntze, *Aster radicans* (Remy) Kuntze, *H. radicans* Remy	Chile (Valparaíso, Libertador Bernardo O’Higgins, Maule, Metropolitan of Santiago) Argentina (Mendoza)
4	*H. arbutoides* Remy	*Aster arbutoides* (Remy) Kuntze, *H. obovatus* Phil., *H. baccharidifolius* Phil., *H. zanartui* (Phil.) Reiche	Chile (Coquimbo, Valparaíso, Libertador Bernardo O’Higgins, Maule, Ñuble, Biobío, Araucanía)
5	*H. baylahuen* Remy subsp. *baylahuen*	*Aster baylahuen* (Remy) Kuntze, *H. domeykoi* Phil., *H. lastarrianus* Remy, *H. medicinalis* Phil	Chile (Atacama, Coquimbo) Argentina (San Juan)
6	*H. baylahuen* Remy subsp. *fluehmannii* (Phil.) Klingenb	*H. fluehmannii* Phil	Chile (Atacama)
7	*H. bezanillanus* (Remy) Reiche	*Aster bezanillanus* (Remy) Kuntze, *Pyrrocoma bezanillana* Remy	Chile (Coquimbo)
8	*H. boelckei* Tortosa & A. Bartoli	-	Argentina (Mendoza)
9	*H. bustillosianus* Remy	*Aster bustillosianus* (Remy) Kuntze, *Aster patagoniensis* Kuntze, *H. australis* Phil., *H. glutinosus* f. *patagonicus* (Phil.) Cabrera, *H. patagonicus* Phil., *H. subandinus* Phil	Chile (Maule, Ñuble, Biobío, Araucanía, Los Lagos) Argentina
10	*H. cerberoanus* (Remy) Reiche subsp. *cerberoanus*	*Aster cerberoanus* (Remy) Kuntze, *Pyrrocoma cerberoana* Remy	Chile (Atacama, Coquimbo) Peru
11	*H. cerberoanus* (Remy) Reiche subsp. *elquianus* Klingenb	-	Chile (Coquimbo)
12	*H. chrysanthemifolius* (Less.) DC.	*Andromachia alternifolia* Kuntze, *Diplopappus chrysanthemifolius* Less., *Grindelia glutinosa* Bertero, *H. berteroi* DC., *H. leucanthemifolius* Phil	Chile (Coquimbo, Valparaíso, Libertador Bernardo O’Higgins, Maule, Ñuble, Biobío, Metropolitan of Santiago)
13	*H. coquimbensis* (Hook. & Arn.) Klingenb	*Aster hirtellus* (Phil.) Kuntze, *Diplopappus coquimbensis* Hook. & Am, *Haplodiscus elatus* Phil., *H. acanthodon* Phil. Reiche, *H. elatus* (Phil.) Reiche, *H. hirtellus* Phil., (Phil.) *H. hirtellus* Phil. var. *hirsutus, H. limarensis* Phil., *H. vidalii* Phil	Chile (Atacama, Coquimbo, Valparaíso, Libertador Bernardo O’Higgins)
14	*H. colliguayensis* M.A.Villalobos, V.Morales & Nic.García	-	Chile (Valparaíso)
15	*H. decurrens* Remy	*Aster remyanus* Kuntze	Chile (Coquimbo, Valparaíso, Libertador Bernardo O’Higgins, Metropolitan of Santiago)
16	*H. deserticola* Phil	*H. involucratus* Phil., *H. rengifoanus* Phil	Chile (Antofagasta, Atacama, Coquimbo)
17	*H. diplopappus* Remy subsp. *diplopappus*	*Aster diplopappus* (Remy) Kuntze, *Diplopappus spinulosus* Hook. & Arn., *H. heterophysus* Phil., *H. pallidus* Phil *H. peteroanus* Phil., *H. reticulatus* Phil	Chile (Valparaíso, Libertador Bernardo O’Higgins, Maule, Metropolitan of Santiago)
18	*H. diplopappus* Remy subsp. *villosus* (Phil.) L. Klingenberg	*Aster villiger* Kuntze, *Diplopappus spinulosus* Hook. & Arn., *H. diplopappus* Remy var. *struthionum* (Speg.) Cabrera, *H. villosus* Phil	Chile (Valparaíso, Libertador Bernardo O’Higgins, Maule, Metropolitan of Santiago) Argentina (Chubut, Mendoza, Santa Cruz)
19	*H. donianus* (Hook. & Arn.) Sch.Bip. ex Reiche	*Diplopappus donianus* Hook. & Arn., *Haplodiscus exserens* Phil., *Haplodiscus tenuifolius* Phil., *H. canescens* var. *exserens* (Phil.) Reiche	Chile (Valparaíso, Libertador Bernardo O’Higgins, Maule, Biobío)
20	*H. foliosus* (Hook. & Arn.) Hook. & Arn. subsp. *foliosus*	*Aster foliosus* (DC.) Kuntze, *Aster polyphyllus* (Phil.) Kuntze, *Diplopappus foliosus* Hook. & Arn., *Haplodiscus densifolius* Phil., *Haplodiscus polyphyllus* Phil., *H. foliosus* DC., *H. phyllophorus* Reiche	Chile (Coquimbo, Valparaíso, Libertador Bernardo O’Higgins, Maule, Metropolitan of Santiago)
21	*H. foliosus* (Hook. & Arn.) Hook. & Arn. subsp. *meyenii* (Walp.) L. Klingenberg	*Aster meyenii* (Walp.) Kuntze, *H. meyenii* Walp	Chile (Coquimbo)
22	*H. glabratus* Phil	*Aster glabratus* (Phil.) Kuntze, *H. arbutoides* Remy var. *glabratus* (Phil.) Reiche	Chile (Valparaíso, Libertador Bernardo O’Higgins, Maule, Metropolitan of Santiago) Argentina (Chubut, Neuquén, Río Negro, Santa Cruz)
23	*H. glutinosus* Cass	*Aster senebierifolius* Kuntze, *Diplopappus coronopifolius* Less., *H. coronopifolius* DC.	Chile (Valparaíso, Libertador Bernardo O’Higgins, Maule, Biobío, Araucania, Los Ríos, Los Lagos, Aisén) Argentina
24	*H. grindelioides* (Less.) DC.	*Aster grindelioides* (Less.) Kuntze, *Aster marginalis* (Phil.) Kuntze, *Aster reversus* Kuntze, *Diplopappus grindelioides* Less., *H. corniculatus* Phil., *H. heterocomus* Phil., *H. marginalis* Phil., *H. reflexus* Phil	Chile (Libertador Bernardo O’Higgins, Maule, Ñuble, Biobío, Araucania, Magallanes, Metropolitan of Santiago) Argentina (Chubut, Mendoza, Neuquén, Río Negro, Santa Cruz)
25	*H. humilis* (Phil.) Reiche	*Haplodiscus humilis* Phil	Chile (Libertador Bernardo O’Higgins, Maule, Ñuble, Biobío, Metropolitan of Santiago)
26	*H. integerrimus* (Hook. & Arn.) H.M. Hall	*Diplopappus integerrimus* Hook. & Arn., *Grindelia acerosa* Bertero, *H. acerosus* Phil., *H. pulchellus* var. *elongaus* Remy, *Steriphe acerosa* Phil	Chile (Coquimbo, Valparaíso, Libertador Bernardo O’Higgins, Maule, Biobío, Metropolitan of Santiago)
27	*H. kingii* (Phil.) Reiche	*Haplodiscus kingii* Phil	Chile (Atacama)
28	*H. linifolius* (Phil.) Reiche	*Aster linodes* (Phil.) Kuntze, *Pyrrocoma linifolia* Phil	Chile (Atacama, Coquimbo)
29	*H. litoralis* Phil	-	Chile (Coquimbo, Valparaíso)
30	*H. macrocephalus* (Poepp. Ex Less.) DC.	*Aster macrocephalus* (Poepp. ex Less.) Kuntze, *Aster spinuliger* Kuntze, *Diplopappus macrocephalus* Poepp. ex Less., *H. caespitosus* Nutt., *H. scaposus* Remy, *H. serrulatus* Reiche, *H. spinulosus* Phil	Chile (Valparaíso, Libertador Bernardo O’Higgins, Maule, Ñuble Biobío, Araucania, Metropolitan of Santiago)
31	*H. maulinus* Klingenb	-	Chile (Maule, Biobío)
32	*H. mendocinus* Tortosa & A. Bartoli	-	Argentina (La Pampa, Mendoza)
33	*H. mieresii* P. Medina & Nic. García	-	Chile (Coquimbo)
34	*H. mucronatus* (Hook. & Arn.) Hook	*Aplopappus macraenus* Gray, *Aster ilicifolius* (Remy) Kuntze, *Aster macraenus* (Remy) Kuntze, *Baccharis hookeriana* DC., *Baccharis mucronata* Hook. & Arn., *Diplopappus mucronatus* Hook. & Arn., *H. axilliflorus* Phil., *H. fonckii* Phil., *H. hookerianus* DC., *H. ilicifolius* Remy, *H. ilicifolius* var. *platylepis* (Phil.) Reiche, *H. platylepis* Phil., *H. macraenus* (Remy) Reiche, *Pyrrocoma macraena* Remy	Chile (Atacama, Coquimbo, Valparaíso)
35	*H. multifolius* Reiche subsp. *baccharidiformis* Klingenb	*-*	Chile (Metropolitan of Santiago)
36	*H. multifolius* Reiche subsp. *multifolius*	*Aster multifolius* (Reiche) Kuntze, *Diplopappus foliolosus* Hook. & Arn., *Diplopappus ilicifolius* Hook. & Arn., *H. rotundifolius* H.M. Hall, *Pyrrocoma foliosa* Phil	Chile (Coquimbo, Valparaíso, Metropolitan of Santiago)
37	*H. multifolius* Reiche subsp. *ovalifolius* Klingenb	-	Chile (Valparaíso, Metropolitan of Santiago)
38	*H. nahuelbutae* Klingenb	-	Chile (Biobío, Araucania)
39	*H. ochagavianus* Phil	*Aster ochayaviensis* Kuntze, *H. reicheanus* H.M. Hall, *H. tiltilensis* Phil., *H. vernicosus* Reiche	Chile (Coquimbo, Valparaíso, Metropolitan of Santiago)
40	*H. paucidentatus* Phil	*Aster glutinosus* (Less.) Kuntze, *Aster oligodontus* Kuntze, *Diplopappus glutinosus* Less., *H. glutinosus* (Less.) DC., *H. glutinosus* f. *spathulata* Cabrera., *H. prostratus* Phil	Chile (Maule, Ñuble Biobío, Araucania, Los Lagos)
41	*H. parvifolius* (DC.) Gay	*Aster parvifolius* (DC.) Kuntze, *Pyrrocoma parvifolia* DC.	Chile (Atacama, Coquimbo)
42	*H. pulchellus* DC.	*Aster valparaisanus* Kuntze	Chile (Coquimbo, Valparaíso, Libertador Bernardo O’Higgins, Maule, Metropolitan of Santiago)
43	*H. philippii* (Kuntze) H.M. Hall	*Aster philippii* Kuntze, *H. breviradiatus* Reiche, *H. paniculatus* Phil	Chile (Atacama, Coquimbo, Valparaíso)
44	*H. pinea* (Phil.) Reiche	*Aster pineus* (Phil.) Kuntze, *Pyrrocoma pinea* Phil	Chile (Coquimbo, Valparaíso)
45	*H. pinnatifidus* Nutt	*Aster andinus* Kuntze, *Aster setiger* (Phil.) Kuntze, *Diplopappus setiger* Hook. & Arn., *H. setigerus* (Phil.) Meigen, *Pyrrocoma nuttalli* Remy, *Pyrrocoma setigera* Phil	Chile (Coquimbo, Valparaíso, Libertador Bernardo O’Higgins, Maule, Metropolitan of Santiago)
46	*H. poeppigianus* (Hook. & Arn.) A. Gray	*Aster griseus* Kuntze, *Diplopappus poeppigianus* Hook. & Arn., *Grindelia canescens* Bertero, *Haplodiscus polycladus* Phil., *H. argenteus* Steud., *H. canescens* (Phil.) Reiche, *Pyrrocoma canescens* Phil	Chile (Valparaíso, Libertador Bernardo O’Higgins, Metropolitan of Santiago)
47	*H. punctatus* (Willd.) Hall	*Aster adalbertii* Kuntze*, Aster pedunculosus* (Remy) Kuntze, *Conyza punctata* Willd., *Diplopappus chamissonis* Less., *H. chamissonis* (Less.) DC., *H. corymbosus* (Phil.) Reiche, *H. pedunculosus* Remy, *H. rosmarinifolius* Reiche, *Steriphe corymbosa* Phil	Chile (Maule, Biobío)
48	*H. pusillus* Klingenb	*Aster cuneifolius* (Nutt.) Kuntze, *Diplopappus bellidifolius* Hook. & Arn., *H. cuneifolius* Nutt., *H. nanus* Phil	Chile (Coquimbo, Valparaíso, Metropolitan of Santiago)
49	*H. racemiger* Klingenb	-	Chile (Atacama, Coquimbo)
50	*H. reicheanus* H.M. Hall	*-*	Chile (Coquimbo, Valparaíso, Metropolitan of Santiago)
51	*H. remyanus* Wedd	*Aster remyanus* (Wedd.) Kuntze, *Haplodiscus latifolius* Phil., *Haplodiscus vernicosus* Phil., *Haplodiscus vernicosus* var. *geissei* Phil.*, H. latifolius* (Phil.) Reiche, *H. prinophyllus* Phil., *Pyrrocoma ilicifolia* Remy	Chile (Atacama, Coquimbo, Valparaíso, Libertador Bernardo O’Higgins, Metropolitan of Santiago)
52	*H. rengifoanus* Remy	*Aster rengifoanus* Kuntze, *Haplodiscus pachyphyllus* Phil., *Pyrrocoma densifolia* Phil	Chile (Antofagasta, Atacama, Coquimbo, Libertador Bernardo O’Higgins)
53	*H. retinervius* (Kuntze) Klingenb	*Aster retinervius* Kuntze, *Haplodiscus ischnos* Phil., *Haplodiscus landbecki* Phil., *Pyrrhocoma reticulata* Phil., *H. ischnos* (Phil.) Reiche, *H. reticulatus* (Phil.) Reiche	Chile (Coquimbo, Valparaíso)
54	*H. rigidus* Phil	*Aster atacamensis* Kuntze	Chile (Antofagasta, Atacama, Coquimbo) Argentina (Catamarca, Salta) Bolivia (Potosí)
55	*H. rosulatus* H.M. Hall	-	Chile (Antofagasta, Atacama, Coquimbo)
56	*H. schumannii* (Kuntze) G.K. Br. & W.D. Clark	*Aster schumannii* Kuntze, *H. armerioides* Phil., *H. poeppigianus* (Hook. & Arn.) A. Gray var. *radiatus* A. Gray, *H. sericeus* Phil., *Steriphe navarroi* Phil	Chile (Valparaíso, Metropolitan of Santiago)
57	*H. scrobiculatus* (Nees) DC.	*Aster cuneifolius* (Nutt.) Kuntze, *Aster densifolius* (Remy) Kuntze, *Diplopappus cuneatus* Hook. & Arn., *Diplopappus scrobiculatus* Nees, *H. densifolius* Remy, *Perezia spathulata* Phil	Chile (Coquimbo, Valparaíso, Libertador Bernardo O’Higgins, Maule, Ñuble, Biobío, Araucania, Metropolitan of Santiago) Argentina (Mendoza, San Juan)
58	*H. setulosus* Klingenb	-	Chile (Maule, Ñuble)
59	*H. stelliger* Remy	*Aster denticulatus* (Phil.) Kuntze, *Aster stelliger* (Remy) Kuntze, *H. denticulatus* (Phil.) Reiche, *Pyrrocoma denticulata* Phil	Chile (Coquimbo)
60	*H. stolpii* Phil	-	Chile (Maule, Ñuble, Biobío, Araucania, Metropolitan of Santiago)
61	*H. taeda* Reiche	*Haplodiscus peteroanus* Phil., *Haplodiscus graveolens* Phil.*, H. graveolens* (Phil.) Reiche	Chile (Valparaíso, Libertador Bernardo O’Higgins, Maule, Metropolitan of Santiago)
62	*H. teillieri* A.Cádiz-Véliz, V.Morales & Nic.García	-	Chile (Coquimbo, Valparaíso)
63	*H. uncinatus* Phil	*Aster uncinatus* (Phil.) Kuntze, *Diplopappus canescens* Hook. & Arn., *H. candolei* Phil*., H. uncinatus* Phil. var. *candolei* (Phil.) Reiche	Chile (Coquimbo, Valparaíso, Libertador Bernardo O’Higgins, Maule, Metropolitan of Santiago)
64	*H. undulatus* Klingenb	-	Chile (Coquimbo, Valparaíso, Metropolitan of Santiago)
65	*H. valparadisiacus* Klingenb	*Diplopappus inuloides* Hook. & Arn., *H. berteroi* var. *lanceolatus* DC., *H. formosus* Phil	Chile (Coquimbo, Valparaíso, Libertador Bernardo O’Higgins, Metropolitan of Santiago)
66	*H. velutinus* Remy subsp. *illinitus* (Phil.) Klingenb	*H. glutinosus* var. *illinitus* (Phil.) Reiche, *H. illinitus* Phil	Chile (Libertador Bernardo O’Higgins, Maule)
67	*H. velutinus* Remy subsp. *longipes* (Phil.) Klingenb	*Aster longipes* (Phil.) Kuntze, *Pyrrocoma longipes* Phil	Chile (Libertador Bernardo O’Higgins, Maule)
68	*H. velutinus* Remy subsp. *velutinus*	*Aster gayanus* Kuntze, *Aster scopiformis* Kuntze, *Diplopappus glutinosus* Hook. & Arn., *Haplodiscus fallax* Phil., *Haplodiscus longiscapus* Phil., *H. fallax* (Phil.) Reiche, *H. stenophyllus* Phil., *H. virgatus* Phil., *Pyrrocoma scaposa* Phil	Chile (Coquimbo, Valparaíso, Libertador Bernardo O’Higgins, Maule, Metropolitan of Santiago) Argentina (Mendoza)
69	*H. vicuniensis* Klingenb	-	Chile (Coquimbo)
70	*H. villanuevae* Phil	-	Chile (Antofagasta, Atacama)

^a^ (Rodriguez et al., 2018; Zuloaga et al., 2019; POWO, 2024).

## 4 Phytochemistry

Available scientific literature provides relevant information on the phytochemistry of the genus *Haplopappus.* However, it must be mentioned that this information refers to only 28 species and subspecies of a total of 70 taxa (Table 1), thus highlighting the largely understudied potential of the genus *Haplopappus* and stressing the need to further investigate its phytochemistry. Moreover, of these 28 taxa for which scientific evidence is available, for the 24 there are less than 35 compounds reported per taxa, whereas the remaining four species are associated to a higher -yet still rather diverse-number of reported compounds, i.e., *H. foliosus* (*n = 146*), *H. velutinus* (*n = 59*), *H. chrysanthemifolius* (*n = 52*), *H. bustillosianus* (*n = 40*).

Regarding the type of metabolites reported in *Haplopappus* species, more than 400 different molecules have been detected in various plant parts of the studied taxa. However, the number of reported compounds per chemical group is highly diverse, to an extent that it raises the question of whether this variability can be solely attributed to differences at a plant metabolic level or it can also be associated with a focus of scientific research towards certain groups of metabolites, e.g., terpenoids and phenolics. Indeed, products of the terpenoid metabolic pathway are by far the most abundant group of molecules reported in the genus *Haplopappus*, including more than 200 compounds, i.e. 54 monoterpenoids (abbreviated as **Mon** in compound codification used in the present review), 60 sesquiterpenoids **(Sqt)**, 107 diterpenoids **(Dit)**, five triterpenoids **(Tri)**, one meroterpenoid **(Mer)** and two steroids **(Str)**. The second most abundant group of reported compounds includes flavonoids (**Flv**; flavonols, *n = 46*; flavones, *n = 20*; flavanones, *n = 8*; flavanonols, *n = 11*) and other products of the metabolic pathway of shikimic acid, i.e., coumarins (**Cum**, *n = 16*), benzoic (**Ben**, *n = 3*) and cinnamic (**Cin**, *n = 12*) acid derivatives. Other compounds reported in the genus *Haplopappus* include alkanes (**Ala**, *n = 29*), alkenes (**Ale**, *n = 4*), alkynes (**Aly**, *n = 1*), alcohols (**Alc**, *n = 5*), ethers (**Eth**, *n = 1*), aromatic hydrocarbons and derivatives (**Arh**, *n = 10*), aldehydes (**Ald**, *n = 8*), ketones (**Ket**, *n = 7*), esters (**Est**, *n = 8*), furanones (**Fur**, *n = 1*), lactones (**Ltn**, *n = 2*) and lactams (**Ltm**, *n = 1*).

The aforementioned compounds as classified per chemical group are detailed in Figures 1–11 and Tables 2–4, and Supplementary Table S1, while their distribution among the studied *Haplopappus* taxa is presented as follows.

**TABLE 2:** Substitution pattern of flavonols and flavones reported in species of the genus *Haplopappus.*

No.	Compound	R_3_	R_5_	R_6_	R_7_	R_8_	R_3'_	R_4'_	R_5'_
Flavonols									
**Flv1**	quercetin	OH	OH	H	OH	H	OH	OH	H
**Flv2**	quercetin 3-methyl ether	OMe	OH	H	OH	H	OH	OH	H
**Flv3**	tamarixetin (quercetin 4′-methyl ether)	OH	OH	H	OH	H	OH	OMe	H
**Flv4**	rhamnazin (quercetin 7,3′-dimethyl ether)	OH	OH	H	OMe	H	OMe	OH	H
**Flv5**	quercetin 3,3′-dimethyl ether	OMe	OH	H	OH	H	OMe	OH	H
**Flv6**	quercetin 3,7-dimethyl ether	OMe	OH	H	OMe	H	OH	OH	H
**Flv7**	ayanin	OMe	OH	H	OMe	H	OH	OMe	H
**Flv8**	retusin (5-hydroxy-3,7,3′,4′-tetramethoxyflavone)	OMe	OH	H	OMe	H	OMe	OMe	H
**Flv9**	3-*O*-acetyl-7-methylquercetin	OAc	OH	H	OMe	H	OH	OH	H
**Flv10**	isoquercitrin (quercetin-3-*β*-D-glucoside)	O-Glu	OH	H	OH	H	OH	OH	H
**Flv11**	hyperoside (quercetin-3-*β*-D-galactoside)	O-Gal	OH	H	OH	H	OH	OH	H
**Flv12**	quercetagetin 3-methyl ether	OMe	OH	OH	OH	H	OH	OH	H
**Flv13**	quercetagetin 3,7-dimethyl ether	OMe	OH	OH	OMe	H	OH	OH	H
**Flv14**	centaureidin	OMe	OH	OMe	OH	H	OH	OMe	H
**Flv15**	betuletol (3,5,7-trihydroxy-6,4′-dimethoxyflavone)	OH	OH	OMe	OH	H	H	OMe	H
**Flv16**	eupatolitin	OH	OH	OMe	OMe	H	OH	OH	H
**Flv17**	rhamnetin	OH	OH	H	OMe	H	OH	OH	H
**Flv18**	isorhamnetin	OH	OH	H	OH	H	OMe	OH	H
**Flv19**	isorhamnetin-3-*β*-D-glucoside	O-Glu	OH	H	OH	H	OMe	OH	H
**Flv20**	isorhamnetin-3-*β*-D-galactoside	O-Gal	OH	H	OH	H	OMe	OH	H
**Flv21**	kaempferol	OH	OH	H	OH	H	H	OH	H
**Flv22**	astragalin (kaempferol 3-*β*-D-glucoside)	O-Glu	OH	H	OH	H	H	OH	H
**Flv23**	isokaempferide (kaempferol 3-methyl ether)	OMe	OH	H	OH	H	H	OH	H
**Flv24**	kaempferol 3-methyl ether 7-*β*-D-glucoside	OMe	OH	H	O-Glu	H	H	OH	H
**Flv25**	rhamnocitrin (kaempferol 7-methyl ether)	OH	OH	H	OMe	H	H	OH	H
**Flv26**	ermanin (kaempferol 3,4′-dimethyl ether)	OMe	OH	H	OH	H	H	OMe	H
**Flv27**	kaempferol 7,4′-dimethyl ether	OH	OH	H	OMe	H	H	OMe	H
**Flv28**	kumatakenin (kaempferol 3,7-dimethyl ether)	OMe	OH	H	OMe	H	H	OH	H
**Flv29**	kaempferol 3,7,4′-trimethyl ether	OMe	OH	H	OMe	H	H	OMe	H
**Flv30**	3-*O*-acetyl-7,4′-dimethylkaempferol	OAc	OH	H	OMe	H	H	OMe	H
**Flv31**	haplopappin	OMe	OH	H	OH	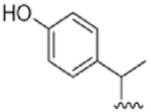	H	OMe	H
**Flv32**	haplopappin A	OH	OH	H	OMe	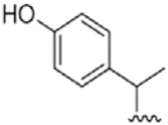	H	OMe	H
**Flv33**	myricetin	OH	OH	H	OH	H	OH	OH	OH
**Flv34**	myricetin 3′,4′-dimethyl ether	OH	OH	H	OH	H	OMe	OMe	OH
**Flv35**	myricetin 3,3′,4′-trimethyl ether	OMe	OH	H	OH	H	OMe	OMe	OH
**Flv36**	myricetin 3,7,4′-trimethyl ether	OMe	OH	H	OMe	H	OH	OMe	OH
**Flv37**	3,8-dimethylherbacetin (5,7,4′-trihydroxy-3,8-dimethoxyflavone)	OMe	OH	H	OH	OMe	H	OH	H
**Flv38**	3,8,4′-trimethylherbacetin (5,7-dihydroxy-3,8,4′-trimethoxyflavone)	OMe	OH	H	OH	OMe	H	OMe	H
**Flv39**	5,7,4′-trihydroxy-3,8,3′-trimethoxyflavone	OMe	OH	H	OH	OMe	OMe	OH	H
**Flv40**	3,5-dihydroxy-3′,4′,6,7-tetramethoxyflavone	OH	OH	OMe	OMe	H	OMe	OMe	H
**Flv41**	santin	OMe	OH	OMe	OH	H	H	OMe	H
**Flv42**	eupatorin	H	OH	OMe	OMe	H	OH	OMe	H
**Flv43**	jaceidin	OMe	OH	OMe	OH	H	OMe	OH	H
**Flv44**	jaceidin 7-methyl ether	OMe	OH	OMe	OMe	H	OMe	OH	H
**Flv45**	penduletin	OMe	OH	OMe	OMe	H	H	OH	H
**Flv46**	pachypodol	OMe	OH	H	OMe	H	OMe	OH	H
Flavones									
**Flv47**	apigenin	H	OH	H	OH	H	H	OH	H
**Flv48**	3,6-dimethoxyapigenin	OMe	OH	OMe	OH	H	H	OH	H
**Flv49**	vicenin-2	H	OH	C-Glu	OH	C-Glu	H	OH	H
**Flv50**	vitexin	H	OH	H	OH	C-Glu	H	OH	H
**Flv51**	isovitexin	H	OH	C-Glu	OH	H	H	OH	H
**Flv52**	isoschaftoside	H	OH	C-Ara	OH	C-Glu	H	OH	H
**Flv53**	luteolin	H	OH	H	OH	H	OH	OH	H
**Flv54**	luteolin 5-glucoside	H	O-Glu	H	OH	H	OH	OH	H
**Flv55**	luteolin 7-glucoside	H	OH	H	O-Glu	H	OH	OH	H
**Flv56**	chrysoeriol	H	OH	H	OH	H	OMe	OH	H
**Flv57**	velutin (luteolin 7, 3′-dimethyl ether)	H	OH	H	OMe	H	OMe	OH	H
**Flv58**	diosmetin	H	OH	H	OH	H	OH	OMe	H
**Flv59**	eupafolin (6-methoxyluteolin)	H	OH	OMe	OH	H	OH	OH	H
**Flv60**	6-methoxyluteolin 4′-methyl ether	H	OH	OMe	OH	H	OH	OMe	H
**Flv61**	cirsiliol (6-methoxyluteolin 7-methyl ether)	H	OH	OMe	OMe	H	OH	OH	H
**Flv62**	hispidulin (scutellarein 6-methyl ether)	H	OH	OMe	OH	H	H	OH	H
**Flv63**	pectolinaringenin	H	OH	OMe	OH	H	H	OMe	H
**Flv64**	scutellarein 6-*β*-D-glucoside	H	OH	O-Glu	OH	H	H	OH	H
**Flv65**	3′,4′-dihydroxyflavone 5-glucoside	H	O-Glu	H	H	H	OH	OH	H
**Flv66**	verbenacoside	H	O-Glu	H	H	H	H	OH	H

**TABLE 3:** Substitution pattern of flavanones and flavanonols reported in species of the genus *Haplopappus.*

No.	Compound	R_3_	R_5_	R_6_	R_7_	R_8_	R_3'_	R_4'_	R_5'_
Flavanones									
**Flv67**	sakuranetin (5,4′-dihydroxy-7-methoxyflavonone)	H	OH	H	OMe	H	H	OH	H
**Flv68**	sakuranetin 4′-methyl ether	H	OH	H	OMe	H	H	OMe	H
**Flv69**	persicogenin	H	OH	H	OMe	H	OH	OMe	H
**Flv70**	sternbin	H	OH	H	OMe	H	OH	OH	H
**Flv71**	eriodictyol	H	OH	H	OH	H	OH	OH	H
**Flv72**	eriodictyol 7,3′-dimethyl ether	H	OH	H	OMe	H	OMe	OH	H
**Flv73**	eriodictyol 7,3′,4′-trimethyl ether	H	OH	H	OMe	H	OMe	OMe	H
**Flv74**	pinostrobin	H	OH	H	OMe	H	H	H	H
Flavanonols									
**Flv75**	7,4′-dimethylaromadendrin	OH	OH	H	OMe	H	H	OMe	H
**Flv76**	7-O-methylaromadenrin	OH	OH	H	OMe	H	H	OH	H
**Flv77**	3-*O*-acetyl-7-*O*-aromadendrin	OAc	OH	H	OMe	H	H	OH	H
**Flv78**	padmatin	OH	OH	H	OMe	H	OH	OH	H
**Flv79**	3-*O*-acetylpadmatin	OAc	OH	H	OMe	H	OH	OH	H
**Flv80**	blumeatin B	OH	OH	H	OMe	H	OH	OMe	H
**Flv81**	7,3′-di-O-methyltaxifolin	OH	OH	H	OMe	H	OMe	OH	H
**Flv82**	dihydromyricetin	OH	OH	H	OH	H	OH	OH	OH
**Flv83**	alpinone 3-acetate	OAc	OH	H	OMe	H	H	H	H

**TABLE 4:** Substitution pattern of coumarins reported in species of the genus *Haplopappus.*

No.	Compound	R_6_	R_7_
**Cum1**	esculetin	OH	H
**Cum2**	esculin	Glu	H
**Cum3**	prenyletin	OH	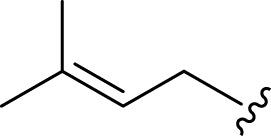
**Cum4**	haplopinol	OH	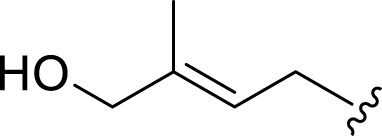
**Cum5**	6-deoxyhaplopinol	H	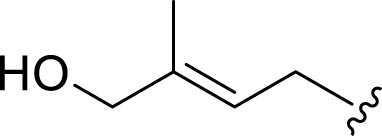
**Cum6**	6-hydroxy-7-(5′-hydroxy-3′,7′-dimethylocta-2′,6′-dien)-oxycoumarin	OH	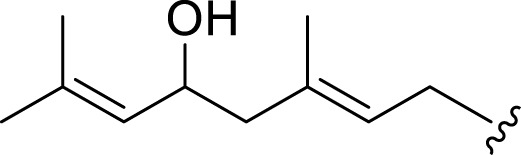
**Cum7**	6-hydroxy-7-(7′-hydroxy-3′,7′-dimethylocta-2′,5′-dien)-oxycoumarin	OH	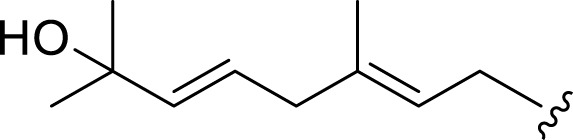
**Cum8**	6-hydroxy-7-[(*E*,*E*)-3′,7′-dimethyl-2′,4′,7′-octatrienyloxy] coumarin	OH	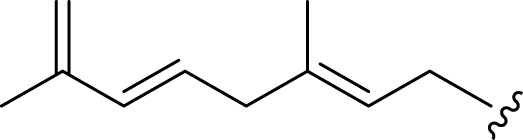
**Cum9**	scopoletin	OMe	H
**Cum10**	7-*O*-prenylscopoletin	OMe	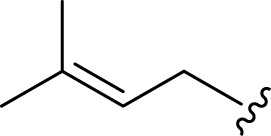
**Cum11**	7-*O*-geranylscopoletin	OMe	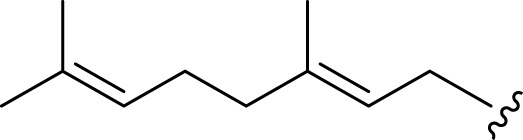
**Cum12**	scoparone	OMe	Me
**Cum13**	hernianin	H	Me
**Cum14**	umbelliferone	H	H
**Cum15**	*O*-prenylumbelliferone	H	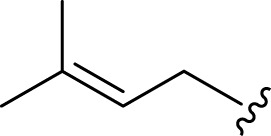

### 4.1 *H. angustifolius* (DC.) Reiche

Information on the chemical composition of *H. angustifolius* is limited to reports of the presence of hentriacontane **(Ala22)**, hexacosanol **(Alc1)**, the diterpenes haplopappic acid **(Dit96)** and its methylester **(Dit97)** and the triterpenes friedelin **(Tri1)** and *epi*-friedelinol **(Tri3)** in the aerial parts of the plant (Silva and Sammes, 1973).

### 4.2 *H. anthylloides* Meyen & Walp

The ketone 4-hydroxyacetophenone **(Ket2)** is the only compound identified in the aerial parts of *H. antylloides* (Zdero et al., 1990).

### 4.3 *H. arbutoides* Remy

The majority of the compounds identified in the aerial parts and/or resin of *H. arbutoides* belong to the diterpenoids group, i.e. 15-oxo-labda-8(17),14*E*-diene-18-oic acid **(Dit32)**, 15-oxo-labda-8(17),14*Z*-diene-18-oic acid **(Dit33)**, labda-8(17),13*E*-dien-15,18-dioic acid 15-methyl ester **(Dit34)**, 15-hydroxylabd-8(17)-en-18-oic acid **(Dit35)**, labd-13(E)-ene-8α,15-diol **(Dit62)**, 13*R-*labdane-8,15-diol **(Dit63)**, 8*α*-hydroxy-*ent*-labd-13(14)*Z*-en-15-al **(Dit64)**, 8*α*-hydroxylabdan-15-al **(Dit65)**, *epi*-manoyl oxide **(Dit68)**, 8,13-epoxy-14-labdeb-3-ol **(Dit74)**, 8,13-epoxy-labdan-15-al **(Dit75)**, 15-oxocleroda-3,13*E*-dien-18-oic acid **(Dit91)**, and 15-oxocleroda-3,13*Z*-dien-18-oic acid **(Dit92)** (Zdero et al., 1991a; Rossomando et al., 1995). Additionally, the aerial parts are reported to contain 4-hydroxyacetophenone **(Ket2)**, the sesquiterpene 1*β*-hydroxy-*β*-cyperone **(Sqt44)** and the flavonols santin **(Flv41)** and penduletin **(Flv45)** (Zdero et al., 1991a; Rossomando et al., 1995).

### 4.4 *H. baylahuen* Remy

The essential oil of the leaves of *H. baylahuen* is reported to contain eicosane **(Ala11)**, benzene **(Arh1)**, azulene **(Arh2)**, naphthalene **(Arh4)**, and the sesquiterpenes bergamotol **(Sqt8)** and *α*-cadinol **(Sqt24)** (Becerra et al., 2010). Phenolic compounds detected in this species include quercetin **(Flv1)**, quercetin 3-methyl ether **(Flv2)**, rhamnetin **(Flv17)**, isorhamnetin **(Flv18)**, kaempferol **(Flv21)**, rhamnocitrin **(Flv25)**, velutin **(Flv57)**, sakuranetin **(Flv67)**, persicogenin **(Flv69)**, sternbin **(Flv70)**, 7,4′-dimethylaromadendrin **(Flv75)**, 7-*O*-methylaromadenrin **(Flv76)**, 7,3′-di-*O*-methyltaxifolin **(Flv81)**, dihydromyricetin **(Flv82)**, prenyletin **(Cum3)**, and 3,5-dicaffeoylquinic acid **(Cin12)** (Schwenker et al., 1967; Hörhammer et al., 1973; Nuñez-Alarcon et al., 1993; Vera et al., 2001; Schmeda-Hirschmann et al., 2015).

### 4.5 *H. bezanillanus* (Remy) Reiche

The compounds detected in the aerial parts of *H. bezanillanus* are the diterpenoid labd-13(E)-ene-8α,15-diol **(Dit62)**, the steroid *β*-sitosterol **(Str2)** and the flavonol jaceidin 7-methyl ether **(Flv44)** (Maldonado et al., 1993).

### 4.6 *H. bustillosianus* Remy

The aerial parts of *H. bustillosianus* contain the alkenes C_11_H_24_ – C_14_H_30_ **(Ala2 – Ala5)**, C_16_H_34_ – C_33_H_68_ **(Ala7 – Ala24)**, along with 3-hydroxyacetophenone **(Ket1)** and the flavonoids santin **(Flv41)** and 3,6-dimethoxyapigenin **(Flv48)** (Urzúa et al., 2007a). Their phenolic profile includes *α*-linalool **(Mon4)**, *α*-pinene **(Mon37)**, *β*-pinene **(Mon38)**, *α*-bisabolol **(Sqt4)**, humulene **(Sqt5)**, *α*-cadinene **(Sqt18)**, *γ*-cadinene **(Sqt20)**, *δ*-cadinene **(Sqt21)**, (−)-isocaryophyllene **(Sqt30)**, *α*-cubebene **(Sqt48)**, *β*-cubebene **(Sqt49)**, *α*-copaene **(Sqt58)**, populifolic acid **(Dit89)** and its methyl ester **(Dit90)**, and thunbergol **(Dit102)** (Urzúa et al., 2007a).

### 4.7 *H. chrysanthemifolius* (Less.) DC

The phytochemistry of *H. chrysanthemifolius* has been thoroughly investigated and various chemical groups of compounds have been identified in this species. Among them, in the flower heads there are present the alkanes C_10_H_22_ – C_19_H_40_ **(Ala1 – Ala10)**, C_21_H_44_ – C_33_H_68_ **(Ala12 – Ala24)**, 2-methyldecalin **(Ala25)**, 2,4,6-trimethyloctane **(Ala26)**, 2,6-dimethylundecane **(Ala27)**, 4,6-dimethylundecane **(Ala28)**, and 2,10-dimethylundecane **Ala29)** (Urzúa et al., 2007a). Furthermore, the terpenoid profile of the species includes *β*-myrcene **(Mon3)**, limonene **(Mon8)**, *α*-pinene **(Mon37)**, *β*-pinene **(Mon38)**, humulene **(Sqt5)**, *δ*-cadinene **(Sqt21)**, (−)-isocaryophyllene **(Sqt30)**, *β*-bulgarene **(Sqt31)**, *γ*-bulgarene **(Sqt32)**, (−)-amorpha-4,11-diene **(Sqt33)**, *α*-cubebene **(Sqt48)**, *β*-cubebene **(Sqt49)**, (−)-calarene **(Sqt50)**, 1,3,4,5,6,7-hexahydro-2,5,5-trimethyl-2*H*-2,4a-ethanonaphthalene **(Sqt51)**, *α*-copaene **(Sqt58)**, 6*α*-hydroxy-*ent*-labd-8(17)-en-15-oic acid **(Dit1)**, 3*β*-acetoxy-*ent*-labd-8(17)-en-15-oic acid **(Dit2)**, and 18*α*-acetoxylabd-8(17)-en-15-oic acid **(Dit3)** (Faini et al., 1999; Urzua et al., 2007b). Regarding the phenolic compounds of *H. chrysanthemifolius*, it is reported the presence of quercetin **(Flv1)**, tamarixetin **(Flv3)**, ayanin **(Flv7)**, myricetin 3,7,4′-trimethyl ether **(Flv36)**, luteolin **(Flv53)**, and diosmetin **(Flv58)** (Faini et al., 1999; Urzua et al., 2007b; Urzúa et al., 2012).

### 4.8 *H. coquimbensis* (Hook. & Arn.) Klingenb

The aerial parts of *H. coquimbensis* (syn. *H. hirtellus* Phil. (Klingenberg, 2007)) contain the terpenoids 7,13-labdadien-15,18-dioic acid 15-methyl ester **(Dit44)** and *epi*-friedelin **(Tri2)**, as well as stigmasterol **(Str1)** (Maldonado et al., 1993). Regarding its flavonoid profile, the following compounds were detected in its aerial parts: kaempferol 7,4′-dimethyl ether **(Flv27)**, kaempferol 3,7,4′-trimethyl ether **(Flv29)**, pachypodol **(Flv46)**, sakuranetin 4′-methyl ether **(Flv68)**, eriodictyol 7,3′-dimethyl ether **(Flv72)**, 7,4′-dimethylaromadendrin **(Flv75)**, and 7,3′-di-*O*-methyltaxifolin **(Flv81)** (Maldonado et al., 1993).

### 4.9 *H. deserticola* Phil

In the aerial parts of *H. deserticola* there were detected the diterpenoids methyl-*ent*-4-*epi*-agath-18-oate **(Dit17)**, dimethyl-*ent*-4-*epi*-agathoate **(Dit18)**, copaiferolic acid **(Dit19)**, copaiferolic acid 15-methyl ester **(Dit20)**, methyl haplodesertoate **(Dit26)**, 8*α*-hydroxyanticopalic acid **(Dit60)**, 8*α*-hydroxyanticopalic acid methyl ester **(Dit61)**, *ent*-19-hydroxy-*cis*-cleroda-3,13(*E*)-dien-15-oic acid **(Dit98)**, and 18-acetoxy-*cis*-cleroda-3,13(*E*)-dien-15-oic acid **(Dit99)**, along with the sesquiterpenoid germacrene D **(Sqt7)** (Zdero et al., 1990; Urzúa Moll et al., 1997; Tojo et al., 1999).

Regarding its phenolic composition, the aerial parts of this species are reported to contain the flavonoids quercetin **(Flv1)**, quercetin 3-methyl ether **(Flv2)**, isokaempferide **(Flv23)**, 3,8-dimethylherbacetin **(Flv37)**, 3,8,4′-trimethylherbacetin **(Flv38)**, and 5,7,4′-trihydroxy-3,8,3′-trimethoxyflavone **(Flv39)**; the coumarins 7-*O*-prenylscopoletin **(Cum10)**, 7-*O*-geranylscopoletin **(Cum11)**, *O*-prenylumbelliferone **(Cum15)** and the dimeric umbelliferone 3,3-dimethylallyl ether **(Cum16)**, as well as the cinnamic acid derivatives chlorogenic acid **(Cin10)**, 3,4-dicaffeoylquinic acid **(Cin11)**, and 3,5-dicaffeoylquinic acid **(Cin12)** (Zdero et al., 1990; Tojo et al., 1999; Schmeda-Hirschmann et al., 2015).

### 4.10 *H. diplopappus* Remy

The resinous exudate of *H. diplopappus* is reported to contain the diterpenoid *ent*-manool **(Dit9)** and its 13-*O*-*β-*xylopyranoside **(Dit8)** (Urzua et al., 1995a).

### 4.11 *H. foliosus* (Hook. & Arn.) Hook. & Arn

*H. foliosus* is the species for which the greatest number of compounds has been reported. Among them, there are the alkanes C_12_H_26_ **(Ala3)**, C_14_H_30_ **(Ala5)**, C_16_H_34_ **(Ala7)**, C_18_H_38_ **(Ala9)**, and C_23_H_48_ – C_33_H_68_ **(Ala14 – Ala24)** (Silva and Sammes, 1973; Urzúa et al., 2000; Urzúa, 2004). Furthermore, the aerial parts of this species contain 11-tricosene **(Ale1)**, hexacosanol **(Alc1)**, ethylresorcinol **(Alc2),** diisopropyl ether **(Eth1)**, *α*-asarone **(Arh3)**, 1,2,3,4,5,6,7,8-octahydro-1-methylphenantrene **(Arh5)**, eugenol **(Arh6)**, styrene **(Arh7)**, safrol **(Arh8)**, elemicin **(Arh9)**, dihydrobenzofuran **(Arh10)**, benzaldehyde **(Ald1)**, 2,3-dichloro-2-methylpropanal **(Ald2)**, *trans*-2-hexenal **(Ald3)**, nonanal **(Ald4)**, decanal **(Ald5)**, 3-ethylbenzaldehyde **(Ald6)**, 4-vinylbenzaldehyde **(Ald7)**, 3-hydroxyacetophenone **(Ket1)**, 3-ethylacetophenone **(Ket3)**, 4-ethylacetophenone **(Ket4)**, dihydro-*α*-ionone **(Ket6)**, 4,4-dimethyl-2-allylcyclohexanone **(Ket7)**, (*Z*)-3-hexenyl acetate **(Est8)**, tetrahydroactinidiolide **(Ltn2)**, 4-phenyl-2-azetidinone **(Ltm1)**, and stigmasterol **(Str1)** (Silva and Sammes, 1973; Urzúa et al., 2000; 2010; Urzúa, 2004; Villagra et al., 2021).

The terpenoid fraction of *H. foliosus* has been thoroughly studied and more than 70 compounds have been reported. Among them, there are the monoterpenoids *cis*-*α*-ocimene **(Mon1)**, *β*-ocimene **(Mon2)**, *β*-myrcene **(Mon3)**, limonene **(Mon8)**, *α*-terpinene **(Mon9)**, *γ*-terpinene **(Mon10)**, terpinen-4-ol **(Mon11)**, terpinolene **(Mon16)**, isoterpinolene **(Mon17)**, *α*-terpineol **(Mon18)**, *p*-menth-2-en-4-ol **(Mon19)**, *trans*-*p*-menth-2-en-1-ol **(Mon21)**, *cis*-*p*-menth-2-en-1-ol **(Mon22)**, *α*-phellandrene **(Mon25)**, *m*-cymene **(Mon27)**, *p*-cymene **(Mon28)**, *p*-cymen-8-ol **(Mon29)**, *o*-cumenol **(Mon30)**, 3-carene **(Mon31)**, thujane **(Mon32)**, *α*-thujene **(Mon33)**, *cis*-(+/−)-4-thujanol **(Mon34)**, 4-thujanol **(Mon35)**, *α*-thujone **(Mon36)**, *α*-pinene **(Mon37)**, *β*-pinene **(Mon38)**, pinocarveol **(Mon39)**, borneol **(Mon42)**, bornyl acetate **(Mon43)**, camphor **(Mon44)**, camphene **(Mon45)**, fenchol **(Mon46)**, 1,5-dimethyl-6-methylenespiro[2.4]heptane **(Mon48)**, sabinene **(Mon49)**, 5-(acetyloxy)-4,6,6-trimethyl-endobiciclo[2.2.1]heptan-2-one **(Mon50)**, ascaridole **(Mon52)**, and tricyclene **(Mon54)** (Urzúa et al., 2000; 2010; Urzúa, 2004; Villagra et al., 2021). The equally diverse sesquiterpenoid fraction includes germacrene D **(Sqt7)**, (1α,7β,10β)-11-hydroxy-4-guaien-3-one **(Sqt9)**, (1β,7β,10β)-1,11-dihydroxy-4-guaien-3-one **(Sqt10)**, (1α,6α,7β,10β)-6,11-dihydroxy-4-guaien-3-one **(Sqt11)**, *α*-selinene **(Sqt13)**, *γ*-selinene **(Sqt14)**, 5-eudesmen-11-ol **(Sqt15)**, *γ*-eudesmol **(Sqt16)**, cadalene **(Sqt17)**, *α*-cadinene **(Sqt18)**, *β*-cadinene **(Sqt19)**, *γ*-cadinene **(Sqt20)**, *δ*-cadinene **(Sqt21)**, guaiol **(Sqt22)**, 1(10),11-eremophiladiene **(Sqt23)**, *α*-cadinol **(Sqt24)**, ionene **(Sqt25)**, 6-(1,1-dimethylethyl)-2,3-dihydro-1,1-dimethyl-3-methylene-1*H*-indene **(Sqt26)**, *δ*-ambrinol **(Sqt27)**, decahydro-3a,8-dimethyl-5-(1-methylethenyl)azulene **(Sqt28)**, 1,2,3,4,5,6,7,8-octahydro-1,4-dimethyl-7-(1-methylethylidene)azulene **(Sqt29)**, *β-*guaiene **(Sqt36)**, (−)-caryophyllene **(Sqt38)**, *epi*-bicyclosesquiphellandrene **(Sqt39)**, *α*-muurolene **(Sqt40)**, *γ*-muurolene **(Sqt41)**, agarospirol **(Sqt42)**, aromadendrene **(Sqt47)**, *α*-cubebene **(Sqt48)**, *β*-cubebene **(Sqt49)**, spathulenol **(Sqt52)**, *β*-bourbonene **(Sqt55)**, *α*-copaene **(Sqt58)**, *β*-copaene **(Sqt59)**, and *β*-ylangene **(Sqt60)** (Labbé et al., 1998; Urzúa et al., 2000; 2010; Urzúa, 2004; Villagra et al., 2021). Much less diverse are the reported di- and triterpenoid profiles of the species, which include 2*α*-hydroxy-*cis*-clero-3,13(*Z*),8(17)-trien-15-oic acid **(Dit87)**, 2*α*-acetoxy-*cis*-clero-3,13(*Z*),8(17)-trien-15-oic acid **(Dit88)**, haplopappic acid **(Dit96)**, friedelin **(Tri1)**, and *epi*-friedelinol **(Tri3)** (Silva and Sammes, 1973; Urzúa et al., 2003).

The flavonoid profile of *H. foliosus* has also been thoroughly investigated and reported to include quercetin 3-methyl ether **(Flv2)**, rhamnazin **(Flv4)**, isoquercitrin **(Flv10)**, hyperoside **(Flv11)**, beturetol **(Flv15)**, eupatolin **(Flv16)**, isorhamnetin **(Flv18)**, isorhamnetin 3-*β*-D-glucoside **(Flv19)**, kaempferol **(Flv21)**, astragalin **(Flv22)**, isokaempferide **(Flv23)**, kaempferol 3-methyl ether 7-*β*-D-glucoside **(Flv24)**, ermanin **(Flv26)**, kumatakenin **(Flv28)**, haplopappin **(Flv31)**, and haplopappin A **(Flv32)** (Ulubelen et al., 1982; Tschesche et al., 1985; Urzúa, 2004).

Furthermore, the following coumarins were detected in *H. foliosus*: esculetin **(Cum1)**, prenyletin **(Cum3)**, scopoletin **(Cum9)**, and scoparone **(Cum12)** (Ulubelen et al., 1982; Urzúa, 2004), along with the benzoic and cinnamic acid derivatives methyl salicylate **(Ben3)**, *trans*-cinnamic acid **(Cin1)**, *cis*-cinnamic acid **(Cin2)**, isobutyl-(*E*)-cinnamate **(Cin3)**, pentyl-(*E*)-cinnamate **(Cin4)**, benzyl-(*E*)-cinnamate **(Cin5)**, and 2-phenylethyl-(*E*)-cinnamate **(Cin6)** (Urzúa et al., 2000; Urzúa, 2004; Villagra et al., 2021).

### 4.12 *H. glutinosus* Cass

The aerial parts of *H. glutinosus* are reported to contain 4-hydroxyacetophenone **(Ket2)**, *β*-farnesene **(Sqt2)**, germacrene D **(Sqt7)**, 6,18-dihydroxy-*ent*-labd-7,13*E*-dien-15-oic acid **(Dit41)**, 4-hydroxybenzoic acid **(Ben1)**, syringic acid **(Ben2)**, *trans*-cinnamic acid **(Cin1)**, caffeic acid **(Cin9)**, and chlorogenic acid **(Cin10)** (Jakupovic et al., 1986; Marambio and Silva, 1996). Furthermore, the flavonoid profile of the species includes isokaempferide **(Flv23)**, ermanin **(Flv26)**, santin **(Flv41)**, jaceidin **(Flv43)**, apigenin **(Flv47)**, 3,6-dimethoxyapigenin **(Flv48)**, luteolin 5- **(Flv54)** and 7- **(Flv55)** glucosides, hispidulin **(Flv62)**, pectolinaringenin **(Flv63)**, 3′,4′-dihydroxyflavone 5-glucoside **(Flv65)**, and verbenacoside **(Flv66)** (Marambio and Silva, 1996; Valant-Vetschera and Wollenweber, 2007).

### 4.13 *H. integerrimus* (Hook. & Arn.) H.M. Hall

Scientific literature only contains information on the flavonoid profile of the leaves of *H. integerrimus* var. *punctatus* (Willd.) G.K.Br. & W.D.Clark, according to which the following compounds were detected: quercetin **(Flv1)**, quercetin 3-methyl ether **(Flv2)**, rhamnazin **(Flv4)**, quercetin 3,3′-dimethyl ether **(Flv5)**, quercetin 3,7-dimethyl ether **(Flv6)**, isoquercitrin **(Flv10)**, isorhamnetin **(Flv18)**, myricetin 3′,4′dimethyl ether **(Flv34)**, and myricetin 3,3′,4′-trimethyl ether **(Flv35)** (Ayanoglu et al., 1981).

### 4.14 *H. litoralis* Phil

The resin of *H. litoralis* is reported to contain the diterpenoids 18*α*-acetoxylabd-8(17)-en-15-oic acid **(Dit3)**,18-hydroxylabd-8(17)-en-15-oic acid **(Dit14)**, (+)-copalic acid **(Dit16)**, and (−)-eperuic acid **(Dit21)** (Urzúa et al., 2004b). Moreover, the flavonols ayanin **(Flv7)** and retusin **(Flv8)** were identified in the resinous exudate of this species (Urzúa et al., 2012).

### 4.15 *H. multifolius* Reiche

The terpenoids 2,9-epoxy-*p*-menth-6-en-8-ol **(Mon51)**, 9-*cis*-*p*-coumaroyloxy-*α*-terpineol **(Sqt12)**, 18-hydroxylabda-7,13(*E*)-dien-15-oic acid **(Dit39)**, and 18-hydroxylabda-7,13(*Z*)-dien-15-oic acid **(Dit42)** are present in the aerial parts of *H. multifolius* (Maatooq et al., 2002). However, the phenolic composition of this species has been more thoroughly investigated and the following compounds have been identified: quercetin **(Flv1)**, quercetin 3-methyl ether **(Flv2)**, isorhamnetin **(Flv18)**, persicogenin **(Flv69)**, sternbin **(Flv70)**, 3-*O*-acetylpadmatin **(Flv79)**, blumeatin B **(Flv80)**, esculetin **(Cum1)**, esculin **(Cum2)**, prenyletin **(Cum3)**, haplopinol **(Cum4)**, 6-deoxyhaplopinol **(Cum5)**, 6-hydroxy-7-(5′-hydroxy-3′,7′-dimethylocta-2′,6′-dien)-oxycoumarin **(Cum6)**, 6-hydroxy-7-(7′-hydroxy-3′,7′-dimethylocta-2′,5′-dien)-oxycoumarin **(Cum7)**, 6-hydroxy-7-[(*E*,*E*)-3′,7′-dimethyl-2′,4′,7′-octatrienyloxy] coumarin **(Cum8)**, hernianin **(Cum13)**, umbelliferone **(Cum14)**, *O*-prenylumbelliferone **(Cum15)**, and 3,5-dicaffeoylquinic acid **(Cin12)** (Chiang et al., 1982; Nuñez-Alarcón and Quiñones, 1995; Urzúa et al., 1995b; Maatooq et al., 2002; Torres et al., 2004; 2006; 2013; Schmeda-Hirschmann et al., 2015).

### 4.16 *H. parvifolius* (DC.) Gay

The group of compounds identified in the aerial parts of *H. parvifolius* includes mainly diterpenoids, as well as the sesquiterpenoids 2,8-dimethyl-2′-vinyl-5-[4-methyl-pent-3-enyl]-chromane **(Sqt43)** and aphanamol I **(Sqt46)** (Zdero et al., 1991b). The diterpenoids detected in this species are 13-hydroxylabda-6,8,14-triene **(Dit27)**, 13-hydroxylabda-6,8(17),14-triene **(Dit28)**, 9*α*,13-epoxy-labda-6,8(17),14-triene **(Dit29)**, 6*β*-acetoxy-13-hydroxylabda-8,14-dien-7-one **(Dit30)**, 6*β*-acetoxy-7*β*,13-dihydroxylabda-8,14-diene **(Dit31)**, 6*β*-acetoxy-13-hydroxylabda-7,14-diene **(Dit47)**, 13-hydroxy-6*α*-butyryloxylabda-7,14-diene **(Dit48)**, 13-hydroxylabda-7,14-diene-6-one **(Dit49)**, 9*α*,13-dihydroxylabda-7,14-dien-6-one **(Dit50)**, 6*α*,13-dihydroxylabda-7,14-dien-17-al **(Dit51)**, isomanool **(Dit52)**, 6*α*-hydroxy-9*α*,13-epoxy-labda-7,14-diene **(Dit53)**, 6*α*-acetoxy-9*α*,13-epoxy-labda-7,14-diene **(Dit54)**, 6*α*-butyryloxy-9*α*,13-epoxy-labda-7,14-diene **(Dit55)**, 5*α*-hydroxy-9*α*,13-epoxy-labda-7,14-diene-6-one **(Dit56)**, 6*α*-acetoxy-9α,13-epoxy-labda-7,14-dien-17-al **(Dit57)**, 6-oxo-14,15-*nor*-labda-7-ene **(Dit58)**, 8*α*,13-dihydroxylabda-6,14-diene **(Dit66)**, 8*α*,13-dihydroxylabda-5,14-dien-7-one **(Dit67)**, *epi*-manoyl oxide **(Dit68)**, 6,7-dehydro-13-*epi*-manoyl oxide **(Dit69)**, 6,7-dehydro-8,13-bis-*epi*-manoyl oxide **(Dit70)**, 13,17-epoxy-labda-5,7,14-triene **(Dit71)**, 9*α*,13-epoxy-5*α*,8*α*-dihydroxylabda-6,14-diene **(Dit72)**, 5*α*-hydroxy-7,8-epoxy-7,8-*seco*-6,7-dehydro-13-*epi*-manoyl oxide **(Dit73)**, haploparvone **(Dit103)**, 5*α*-hydroxyhaploparvone **(Dit104)**, haploparviolide **(Dit105)**, 1,1,5,6-tetramethyl-4-[3-hydroxy-3-methyl-pent-(4)-enyl]-tetralin **(Dit106)**, and 1,1,5-trimethyl-6-(3-hydroxy-3-methyl-pent-4-enyl)-tetralin **(Dit107)** (Zdero et al., 1991b).

### 4.17 *H. poeppigianus* (Hook. & Arn.) A. Gray

The aerial parts of *H. poeppigianus* (syn. *H. canescens* (Phil.) Reiche (Klingenberg, 2007)) contain the flavonoid compounds centaureidin **(Flv14)**, myricetin **(Flv33)**, chrysoeriol **(Flv56)**, diosmetin **(Flv58)**, hispidulin **(Flv62)**, and scutellarein 6-*β*-D-glucoside **(Flv64)** (Oksuz et al., 1981).

### 4.18 *H. paucidentatus* Phil

The aerial parts of *H. paucidentatus* contain 4-hydroxyacetophenone **(Ket2)** and the terpenoids germacrene D **(Sqt7)**, caryophyllene oxide **(Sqt34)**, 8-oxo-*β*-cyperone **(Sqt45)**, 18-hydroxy-friedolabd-5-en-15-oic acid **(Dit78)**, 18-hydroxy-*cis*-cleroda-3-en-15-oic acid (10*βH*, 16*ξ,* 19*β*, 17*β*, 20*α* form) **(Dit83)**, 19-hydroxy-*cis*-cleroda-3-en-15-oic acid (10*βH*, 16*ξ,* 19*β*, 17*β*, 20*α* form) **(Dit85)**, 18-hydroxy-*cis*-cleroda-3,13(*E*)-dien-15-oic acid **(Dit93)**, and 18-acetoxy-*cis*-cleroda-3,13(*E*)-dien-15-oic acid **(Dit99)** (Jakupovic et al., 1986).

### 4.19 *H. pulchellus* DC

Regarding the compounds identified in the aerial parts of *H. pulchellus*, those include the diterpenoids 7*α*-hydroxylabd-8(17)-en-15,18-dioic acid **(Dit4)**, labd-7-en-15,18-dioic acid **(Dit36)**, 18-acetoxy-friedolabd-5-en-15-oic acid **(Dit76)**, 18-acetoxy-friedolabd-5-en-7-one-15-oic acid **(Dit77)**, 18-hydroxy-friedolabd-5-en-15-oic acid **(Dit78)**, 18-hydroxy-7-oxo-friedolabd-5-en-15-oic acid **(Dit79)**, friedolabd-5-en-15,18-dioic acid **(Dit80)**, and 15-hydroxy-friedolabd-5-en-18-oic acid **(Dit81)** (Zdero et al., 1991a).

### 4.20 *H. remyanus* Wedd

The esters benzenepropanoic acid, 2-methyl-6-methylene-2,7-octadienyl ester **(Est3)**, (±)-1-acetoxy-2-(p-tolyl)-2-propanol **(Est4)**, 2-hydroxy-2-(4-methylphenyl)propyl benzenepropanoate **(Est5)**, 2-hydroxy-2-(4-methyl-3-cyclohexen-1-yl)propyl benzenepropanoate **(Est6)**, and 2-hydroxy-2-(4-methyl-3-cyclohexen-1-yl)propyl 3-phenyl-2-propenoate **(Est7)** have been detected in the aerial parts of *H. remyanus* (Zdero et al., 1991a). Regarding its terpenoid profile, the species contains uroterpenol **(Mon12)**, 9-benzoyloxy-(1-formyl)-*α*-terpineol **(Mon13)**, 9-benzoyloxy-*α*-terpineol **(Mon14)**, 7-hydroxy-9-benzoyloxy-*α*-terpineol **(Mon15)**, 8-hydroxy-9-acetoxy-*β*-phellandrene **(Mon26)**, 18-hydroxylabda-7,13(*E*)-dien-15-oic acid **(Dit39)**, 18-acetoxy-labda-7,13(*E*)-dien-15-oic acid **(Dit40)**, and 18-dihydrocinnamoyloxy-labda-7,13*E*-dien-l5-oic acid **(Dit46)** (Zdero et al., 1991a; Faini et al., 2011). Morever, the following flavonoid compounds are present in *H. remyanus*: quercetin **(Flv1)**, 3-*O*-acetyl-7-methylquercetin **(Flv9)**, kaempferol 7,4′-dimethyl ether **(Flv27)**, kaempferol 3,7,4′-trimethyl ether **(Flv29)**, 3-*O*-acetyl-7,4′-dimethylkaempferol **(Flv30)**, sakuranetin 4′-methyl ether **(Flv68)**, eriodictyol **(Flv71)**, pinostrobin **(Flv74)**, 7,4′-dimethylaromadendrin **(Flv75)** and alpinone 3-acetate **(Flv83)** (Zdero et al., 1991a; Faini et al., 2011).

### 4.21 *H. rengifoanus* Remy

The aerial parts and/or leaves of *H. rengifoanus* are reported to contain the sesquiterpenoid liguloxide **(Sqt57)** and the flavonoids quercetagetin 3-methyl ether **(Flv12)**, quercetagetin 3,7-dimethyl ether **(Flv13)**, isorhamnetin **(Flv18)**, isorhamnetin 3-*β*-D-glucoside **(Flv19)**, isorhamnetin 3-*β*-D-galactoside **(Flv20)**, apigenin **(Flv47)**, luteolin **(Flv53)**, and scutellarein 6-*β*-D-glucoside **(Flv64)** (Ulubelen et al., 1981; Zdero et al., 1991a).

### 4.22 *H. rigidus* Phil

The diterpenoids rigiduside **(Dit6)**, 18-acetoxy-*cis*-clerode 3,13(*Z*)-dien-15 oic acid **(Dit82)**, rigidusol **(Dit100)**, and deacetylrigidusol **(Dit101)** are present in the aerial parts of *H. rigidus* (Morales et al., 2000a; 2000b; 2003). Furthermore, the flavonoids quercetin 3-methyl ether **(Flv2)**, beturetol **(Flv15)**, kaempferol **(Flv21)**, isokaempferide **(Flv23)**, sakuranetin **(Flv67)** and sternbin **(Flv70)** were detected in the aerial parts (Morales et al., 2000a; 2003; 2009; Schmeda-Hirschmann et al., 2015), along with 3,5-dicaffeoylquinic acid **(Cin12)** (Schmeda-Hirschmann et al., 2015).

### 4.23 *H. schumannii* (Kuntze) G.K. Br. & W.D. Clark

The alkanes C_23_H_48_ – C_31_H_64_ **(Ala14 – Ala22)** and C_33_H_68_ **(Ala14)** have been identified in the aerial parts of *H. schumannii*, along with 1-octadecyne **(Aly1)**, dihydro-*α*-ionone **(Ket6)**, and the lactone tetrahydroactinidiolide **(Ltn2)** (Urzúa et al., 2004a). The terpenoid profile of this species includes the sesquiterpenoids *β*-cadinene **(Sqt19)**, *β*-bourbonene **(Sqt55)**, and globulol **(Sqt56)**, as well as the diterpenoids manool **(Dit7)**, (−)-eperuic acid **(Dit21)**, *epi*-manool **(Dit25)**, 8*α*-hydroxylabdan-15-oic acid **(Dit59)**, and 2-oxoclerod-3-en-15-oic acid **(Dit86)** (Urzúa et al., 1997; 2004a). Moreover, the flavonoids quercetin **(Flv1)**, isoquercitrin **(Flv10)**, vicenin-2 **(Flv49)**, vitexin **(Flv50)**, and isovitexin **(Flv51)** are present in the leaves of *H. schumannii* (Ates et al., 1982).

### 4.24 *H. scrobiculatus* (Nees) DC

The presence of the terpenoids *α-*farnesene **(Sqt1)**, 18-hydroxymanool **(Dit15)**, and 2-oxokolavenic acid **(Dit94)** has been reported in the case of the aerial parts and resinous exudates of *H. scrobicultus* (Rossomando et al., 1995; Urzúa et al., 2004b). However, the largest group of compounds in this species is that of phenolics, namely, quercetin **(Flv1)**, isoquercitrin **(Flv10)**, isorhamnetin **(Flv18)**, isorhamnetin 3-*β*-D-glucoside **(Flv19)**, rhamnocitrin **(Flv25)**, santin **(Flv41)**, eupatorin **(Flv42)**, penduletin **(Flv45)**, vicenin-2 **(Flv49)**, vitexin **(Flv50)**, isovitexin **(Flv51)**, isoschaftoside **(Flv52)**, eupafolin **(Flv59)**, 6-methoxyluteolin 4′-methyl ether **(Flv60)**, cirsiliol **(Flv61)**, and esculetin **(Cum1)** (Ates et al., 1982; Rossomando et al., 1995; Urzúa et al., 2012).

### 4.25 *H. taeda* Reiche

The terpenoid profile of *H. taeda* includes taedol **(Mon41)**, 18-hydroxylabda-7,13(*E*)-dien-15-oic acid **(Dit39)**, 7,13-labdadien-15,18-dioic acid **(Dit43)**, cleroda-3,13 (*E*)-dien-15,18-diol **(Dit95)**, and 18-acetoxy-*cis*-cleroda-3,13(*E*)-dien-15-oic acid **(Dit99)** (Marambio and Silva, 1989; Torres et al., 2005; Faini et al., 2007; 2008). However, scientific literature provides more information on the phenolic composition of this species, with the following compounds being reported: quercetin **(Flv1)**, quercetin 3-methyl ether **(Flv2)**, quercetin 3,7-dimethyl ether **(Flv6)**, kaempferol **(Flv21)**, sakuranetin **(Flv67)**, sternbin **(Flv70)**, eriodictyol 7,3′-dimethyl ether **(Flv72)**, eriodictyol 7,3′,4′-trimethyl ether **(Flv73)**, 3-*O*-acetyl-7-*O*-aromadendrin **(Flv77)**, padmatin **(Flv78)**, 3-*O*-acetylpadmatin **(Flv79)**, 9-*trans*-*p*-coumaroyloxy-α-terpineol **(Cin7)**, 7-*trans*-*p*-coumaroyloxy-taedol **(Cin8)**, chlorogenic acid **(Cin10)**, 3,4-dicaffeoylquinic acid **(Cin11)**, and 3,5-dicaffeoylquinic acid **(Cin12)** (Marambio and Silva, 1989; Faini et al., 2007; 2008; Schmeda-Hirschmann et al., 2015).

### 4.26 *H. uncinatus* Phil

The alkanes C_23_H_48_ – C_31_H_64_ **(Ala14 – Ala22)** and C_33_H_68_ **(Ala14)** have been identified in the resinous exudates and/or aerial parts of *H. uncinatus* (Urzúa et al., 2000; 2004a; 2006), along with 2,7-dimethyl-5-(1-methylethenyl)-1,8-nonadiene **(Ale3)** and 3,5-dihydroxy-3′,4′,6,7-tetramethoxyflavone **(Flv40)** (Urzúa et al., 2004a; 2006). Regarding its terpenoid profile, the species is reported to synthesize 3,3,7,7-tetramethyl-5-(2-methyl-1-propenyl)-tricyclo[4.1.0.0(2,4)]heptane **(Mon53)**, the sesquiterpenoids cadalene **(Sqt17)**, aromadendrene **(Sqt47)**, *α*-cubebene **(Sqt48)**, *β*-cubebene **(Sqt49)**, spathulenol **(Sqt52)**, cedryl acetate **(Sqt53)**, *β*-bourbonene **(Sqt55)**, globulol **(Sqt56)**, *α*-copaene **(Sqt58)**, as well as the clerodane diterpenoid 18-acetoxy-*cis*-cleroda-3-en-15-oic acid (10*βH*, 16*ξ,* 19*β*, 17*β*, 20*α* form) **(Dit84)** (Urzúa et al., 2000; 2004a; 2006).

### 4.27 *H. velutinus* Remy; *H. velutinus* Remy subsp. *illinitus* (Phil.) Klingenb

Several compounds are reported to be present in both *H. velutinus* and the subspecies *H. velutinus* subsp. *illinitus.* These are the alkanes C_23_H_48_ – C_31_H_64_ **(Ala14 – Ala22)** and C_33_H_68_ **(Ala14)**, 5,5-dimethyl-2(5*H*)-furanone **(Fur1)**, *β*-myrcene **(Mon3)**, limonene **(Mon8)**, *α*-pinene **(Mon37)**, *β*-pinene **(Mon38)**, labd-7-en-15,18-dioic acid-18*α*-methylester **(Dit37)**, *β*-sitosterol **(Str2)**, and quercetin **(Flv1)** (Latorre et al., 1990; Marambio and Silva, 1996; Faini et al., 2002; Urzúa et al., 2004a; Echeverría et al., 2019).

In contrast, compounds solely identified in *H. velutinus* include 3-ethyl-1,4-hexadiene **(Ale2)**, 2-nonyn-1-ol **(Alc3)**, 2-pentadecen-1-ol **(Al4)**, *n*-dodecenyl-1-ol **(Alc5)**, vanillin **(Ald8)**, picein **(Ket5)**, lavender lactone **(Ltn1)**, linalyl anthranilate **(Mon5)**, davanone **(Mon6)**, davana ether **(Mon7)**, 1,2:8,9-diepoxy-*p*-menthane **(Mon19)**, *cis*-*p*-menth-2-en-1-ol **(Mon22)**, *trans*-pulegone oxide **(Mon23)**, *α*-campholenal **(Mon24)**, *m*-cymene **(Mon27)**, *α*-thujene **(Mon33)**, pinocarveol **(Mon39)**, *trans*-2-pinanol **(Mon40)**, *cis-*verbenol **(Mon47)**, *α*-sinensal **(Sqt3)**, humulene epoxide II **(Sqt6)**, caryophyllene oxide **(Sqt34)**, *α*-guaiene **(Sqt35)**, (−)-oplopanone **(Sqt37)**, spathulenol **(Sqt52)**, patchouli alcohol **(Sqt54)**, dehydropinipholic acid 19-methyl ester **(Dit11)**, 4*α*-hydroxy-18-norlabd-8(17)-en-15-oic acid **(Dit12)**, 4*β*-hydroxy-19-norlabd-8(17)-en-15-oic acid **(Dit13)**, 18-hydroxylabd-8(17)-en-15-oic acid **(Dit14)**, 7,13-(*E*)-labdadien-15,18-dioic-acid-18-methyl ester **(Dit45)**, friedelin **(Tri1)**, *epi*-friedelinol **(Tri3)**, taraxerol **(Tri4)**, erythrodiol **(Tri5)**, stigmasterol **(Str1)**, isoquercitrin **(Flv10)**, isokaempferide **(Flv23)**, kumatakenin **(Flv28)**, luteolin **(Flv53)**, and scopoletin **(Cum9)** (Urzúa and Mendoza, 1989; Urzúa et al., 1991; Urzúa et al., 1995a; 2004a; Urzua and Mendoza, 1993; Marambio and Silva, 1996; Echeverría et al., 2019).

The group of compounds identified solely in the subspecies *H. velutinus* subsp. *illinitus* consists of 3,3,5,5-tetramethylcyclopentene **(Ale4)**, methyl octanoate **(Est1)**, 5-methyl-octanoic acid methyl ester **(Est2)**, *β*-cadinene **(Sqt19)**, procerin **(Mer1)**, as well as the diterpenoids 7*α*-hydroxylabd-8(17)-en-15,18-dioic acid-15-methylester **(Dit5)**, pinifolic acid 15-methyl ester **(Dit22)**, pinifolic acid 18-methyl ester **(Dit23)**, pinifolic acid dimethyl ester **(Dit24)**, labd-7-en-15,18-dioic acid **(Dit36)**, labd-7-en-15,18-dioic acid-15-methylester **(Dit38)**, and 7-oxo-labd-8(9)-en-15,18-dioic acid-15-methylester **(Dit10)**, (Faini et al., 2002; Urzúa et al., 2004a).

## 5 Traditional uses and evidence-based pharmacological activities related to human health

### 5.1 Traditional uses

The plants of the genus *Haplopappus* are of high medicinal value and form essential part of the traditional medicines of the Andean region (Chile, Argentina), where the genus presents high endemicity. *Haplopappus* species and their preparations have traditionally been associated with numerous health benefits, associated with multiple aspects of the human health and also with veterinary applications (Table 5).

**TABLE 5:** Traditional uses of *Haplopappus* species.

Species	Plant part(s) – preparation(s)	Traditional use(s)	References
*Haplopappus* spp.	whole plant (alone or combined with *Satureja parvifolia* or *Lycopodium Saururus*); aerial parts; leaf/aerial parts infusion (with or without milk); stem juice; resin (applied externally or ingested)	antidiarrheic; antiseptic; antispasmodic; antitussive; aphrodisiac; cholagogue; choleretic; cicatrizant (in particular, to treat horses); digestive; disinfectant; emmenagogue; hepatic; stimulant; sudorific; against altitude sickness, abdominal colic, dysentery, chronic dyspepsia, colds, flu and urinary diseases	Alonso, 2005; de Mösbach (1992), Hoffmann et al. (1992), Mellado Campos (1996), Ministerio de Salud (2010), Montes and Wilkomirsky (1987), Ratera and Ratera (1980), Schrickel and Bittner (2001)
*H. baylahuen*	whole plant; aerial parts; leaf/aerial parts infusion; leaf decoction; stem juice; taken with milk	aphrodisiac; antidiarrheic; antirheumatic; antiseptic; antispasmodic; antitussive; antiviral, astringent; carminative; cholagogue; choleretic; cicatrizant (in particular, to treat horses and other animals); digestive; disinfectant; emmenagogue; expectorant; hepatic; stimulant; stomachic; against altitude sickness, chronic hemorrhagic intestinal inflammation, colds, flu, flatulent dyspepsia, dysentery, gastritis, male and female hormonal disorders, pneumonia, pains provoked by air currents, genital, renal and urinary disorders	Cárdenas (1998), Del Vitto et al. (2010), Espinoza (1897), Gómez-Parra and Siarez Flores (1995), Hoffmann et al. (1992), Houghton and Manby (1985), Laval (1957), Madaleno and Delatorre-Herrera (2013), Ministerio de Salud (2010), Montes and Wilkomirsky (1987), Mostny et al. (1954), Munizaga (1963), Munizaga and Gunkel (1958), Muñoz S. et al. (1981), Murillo (1861), 1889; Remington and Woods (1918), Serracino et al. (1974), Steinmetz (1954), Vogel et al. (2005b)
*H. multifolius*	whole plant; leaf infusion	antidiarrheic; antiseptic; digestive; emmenagogue; hepatic; stomachic; against dysentery and urinary disorders	Muñoz S. et al. (1981), Vogel et al. (2005b)
*H. remyanus*	whole plant; leaf infusion	antidiarrheic; antiseptic; antispasmodic; digestive; emmenagogue; hepatic; stomachic; against dysentery and urinary disorders	Montes and Wilkomirsky (1987), Muñoz S. et al. (1981), Vogel et al. (2005b)
*H. rigidus*	whole plant; aerial parts infusion; taken with milk; decoction with fruits of *Opuntia camachoi* Espinosa	antirheumatic; antitussive; aphrodisiac; diuretic; febrifuge; hepatic; laxative; stomachic; against colds, flu, pains provoked by air currents, pneumonia, renal colic, cardiac pain, gastrointestinal, ovary and urinary disorders; against veterinary ailments	Aldunate et al. (1981), Gómez et al. (1997), Hoffmann et al. (1992), Mellado Campos (1996), Monterrey (1996), Montes and Wilkomirsky (1987), Muñoz S. et al. (1981), Ratera and Ratera (1980), Villagrán et al. (2003), 1998; Wickens (1993)
*H. taeda*	whole plant; resinous leaves; leaf infusion	antidiarrheic; antiseptic; digestive; emmenagogue; hepatic; stomachic; against dysentery, intestinal and urinary disorders	Faini et al. (2007), Vogel et al. (2005b)

The main health benefits traditionally attributed to different preparations of *Haplopappus* plants are associated with pathologies of the human alimentary tract and metabolism. Various species and preparations have widespread use as digestives, antidiarrheic, remedies against dyspepsia, dysentery and gastrointestinal ailments, in general.

Moreover, there are reported several traditional uses associated with the human genitourinary system, with *Haplopappus* preparations being considered as aphrodisiacs, emmenagogues, diuretic and as remedies against urinary and renal disorders and colics or even against male and female hormonal disorders.

Other traditional uses are associated with health benefits for the human respiratory (antitussives, expectorants, cold remedies) and nervous (stimulant, antispasmodic) system, as well as with their role as disinfectants.

Finally, it is well-documented in traditional Andean medicines the use of *Haplopappus* preparations as cicatrizants with veterinary applications, especially to treat horses’ wounds.

It has to be mentioned that *H. baylahuen* Remy is recognized by the Chilean health authorities as a traditional herbal medicine against liver diseases, abdominal colics, chronic dyspepsia, kidney stones, flus and colds, as well as an aphrodisiac and wound disinfectant (Ministerio de Salud, 2010). Meanwhile, pharmaceutical products that include *bailahuén*, e.g., the formulations ‘Ulcenat’ and ‘Ubenat’ (Grüne Leben) and ‘Bailahuen extracto fluido’ (Knop Laboratorios S.A.) are commercialized in Chile as treatments against digestive disorders. However, there are no internationally or nationally established norms and/or protocols regarding quality, standardization, safety, and adulteration control of *bailahuén* preparations and commercial products.

### 5.2 Evidence-based pharmacological activity related to the human health

Scientific literature provides evidence related to various human health-promoting effects of extracts and isolated compounds of *Haplopappus* species (Table 6), with their inhibitory effect against human pathogens of bacterial origin being the most thoroughly investigated.

**TABLE 6:** Biological activity attributed to the species of the genus *Haplopappus*.

Biological activity	Plant species	Plant part(s)	Type of extract and/or isolated compound	Outcome	References
Antibacterial	*H. anthylloides*	resin	extract (CH_2_Cl_2_)	*In vitro* growth inhibition of *Bacillus anthracis*, *B. pumilis, B. subtilis, Escherichia coli, Micrococcus flavus, M. luteus, Proteus vulgaris, Pseudomonas aeruginosa, Staphylococcus aureus*, *S. epidermidis*	Urzúa et al. (1995b)
	*H. baylahuen*	aerial parts	decoction, extracts (EtOH, EtOAc)	*In vitro* growth inhibition of *Acremonium falciforme*, *Bacillus subtilis*, *Staphylococcus aureus*	Lazo, (1990)
		leaves	extract (H_2_O/EtOH)	Bactericide activity against *Salmonella enteritidis* and inhibition of its ability to form biofilm, express adrA/hilA genes and adhere to Caco-2 cells	Elgueta et al. (2021)
	*H. chrysanthemifolius*	resin	extract (MeOH)	*In vitro* growth inhibition of *Bacillus cereus, B. subtilis, Enterococcus faecalis, Listeria monocytogens, Micrococcus luteus, Staphylococcus aureus*	Urzúa et al., 2004a, 2012
	*H. deserticola*	resin	18-acetoxy-*cis*-cleroda-3,13(*E*)-dien-15-oic acid **(Dit99)**	Bactericidal effect against *Streptococcus mutans*	Urzúa Moll et al. (1997)
	*H. diplopappus* subsp. *diplopappus*	resin	extract (CH_2_Cl_2_)	*In vitro* growth inhibition of *Bacillus anthracis, B. pumilis, B. subtilis Bordetella bronchiseptica, Micrococcus flavus, M. luteus, Proteus vulgaris, Pseudomonas aeruginosa, Staphylococcus aureus*, *S. epidermidis*	Urzúa et al. (1995b)
			13-*O*-*β-*xylopyranosyl-*ent*-manool **(Dit8)**		Urzúa et al. (1995a)
	*H. foliosus*	resin	extracts (MeOH, CH_2_Cl_2_)	*In vitro* growth inhibition of *Bacillus anthracis, B.cereus, B. coagulans, B. pumilis, B. subtilis*, *Micrococcus luteus, Proteus vulgaris, Staphylococcus aureus*, *S. epidermidis*	Urzúa et al., 1995b, 2003; Urzúa and Mendoza, 2001
			2*α*-hydroxy-*cis*-clero-3,13(*Z*),8(17)-trien-15-oic acid **(Dit87)**; 2*α*-acetoxy-*cis*-clero-3,13(*Z*),8(17)-trien-15-oic acid **(Dit88)**	*In vitro* growth inhibition of *Bacillus cereus, B. coagulans, B. subtilis*, *Micrococcus luteus, Staphylococcus aureus*	Urzúa et al. (2003)
	*H. litoralis*	resin	extract (MeOH)	*In vitro* growth inhibition of *Bacillus cereus, B. subtilis, Enterococcus faecalis, Listeria monocytogens, Micrococcus luteus, Staphylococcus aureus*	Urzúa et al., 2004, 2012
	*H. multifolius*	aerial parts	esculetin **(Cum1)**	*In vitro* growth inhibition and bactericide effect against *Escherichia coli, Sarcina lutea*, *Staphylococcus aureus*	Chiang et al. (1982)
			prenyletin **(Cum3)**	*In vitro* growth inhibition and bactericide effect against *Sarcina lutea, Staphylococcus aureus*	
			haplopinol **(Cum4)**	*In vitro* growth inhibition and bactericide effect against *Escherichia coli, Staphylococcus aureus*	
		aerial parts	extracts (EtOH), infusion	*In vitro* growth inhibition of *Bacillus cereus, B. subtilis, Staphylococcus aureus, S. epidermidis, S. pyogenes*	Padilla et al. (2021)
		resin	extract (CH_2_Cl_2_)	*In vitro* growth inhibition of *Bacillus anthracis, B. pumilis, B. subtilis*, *Bordetella bronchiseptica, Micrococcus flavus Proteus vulgaris, Pseudomonas aeruginosa, Staphylococcus aureus*	Urzúa et al. (1995b)
	*H. rigidus*	aerial parts	extracts (EtOH/H_2_O, CHCl_3_, EtOAc)	*In vitro* growth inhibition of *Bacillus cereus, B. subtilis, Corynobacterium minutissimum, Enterococcus faecalis*, *Listeria monocytogenes, Staphylococcus aureus*, *S. lugdunesis*	Morales et al., 2003; Ortiz et al., 2019
	*H. schumannii*	resin	extract (CH_2_Cl_2_)	*In vitro* growth inhibition of *Bacillus anthracis, B. pumilis, B. subtilis*, *Bordetella bronchiseptica, Escherichia coli, Micrococcus flavus, M. luteus, Proteus vulgaris, Staphylococcus aureus*, *S. epidermidis*	Urzúa et al. (1995b)
	*H. scrobiculatus*	resin	extracts (MeOH, CH_2_Cl_2_)	*In vitro* growth inhibition of *Bacillus anthracis, B. cereus, B. pumilis, B. subtilis*, *Enterococcus faecalis, Escherichia coli, Listeria monocytogens, Micrococcus flavus, M. luteus, Proteus vulgaris, Staphylococcus aureus*, *S. epidermidis*	Urzúa et al., 1995b; Urzúa et al., 2004, 2012
	*H. taeda*	aerial parts	extracts (EtOH), infusion	*In vitro* growth inhibition of *Bacillus cereus, B. subtilis, Staphylococcus agalactiae, S. aureus, S.epidermidis, S. pyogenes*	Padilla et al. (2021)
	*H. uncinatus*	resin	extract (MeOH)	*In vitro* growth inhibition of *Bacillus cereus, B. coagulans, B. subtilis*, *Micrococcus luteus, Staphylococcus aureus*	Urzúa and Mendoza, (2001)
			extract (CH_2_Cl_2_)	*In vitro* growth inhibition of *Bacillus anthracis, B. pumilis, B. subtilis*, *Bordetella bronchiseptica, Escherichia coli, Micrococcus flavus, M. luteus, Proteus vulgaris, Staphylococcus aureus*, *S. epidermidis*	Urzúa et al. (1995b)
		aerial parts	resin	*In vitro* growth inhibition of *Bacillus cereus, B. subtilis*, *Micrococcus luteus*	Urzúa et al. (2006)
			18-acetoxy-*cis*-cleroda-3-en-15-oic acid (10*βH*, 16*ξ,* 19*β*, 17*β*, 20*α* form) **(Dit84)**		
	*H. velutinus*	resin	extract (CH_2_Cl_2_)	*In vitro* growth inhibition of *Bacillus anthracis, B. pumilis, B. subtilis*, *Bordetella bronchiseptica, Proteus vulgaris, Micrococcus flavus, M. luteus, Staphylococcus aureus*, *S. epidermidis*	Urzúa et al. (1995b)
	*H. velutinus* subsp. *illinitus*	resin	extract (CH_2_Cl_2_)	*In vitro* growth inhibition of *Bacillus anthracis, B. pumilis, B. subtilis*, *Bordetella bronchiseptica, Micrococcus flavus, Pseudomonas aeruginosa, Staphylococcus aureus*	Urzúa et al. (1995b)
Antidysenteric	*H. baylahuen*	resin	extract suspended in milk, cream or almond emulsion	Symptomatic treatment of dysentery in humans	Fingland, (1903)
Anti-inflammatory	*H. baylahuen*	aerial parts	aqueous extract	Inhibition of carrageenan-induced edema in rats	Adzet and Gene, (1991)
	*H. multifolius*	leaves	esculetin **(Cum1)**; esculin **(Cum2)**; prenyletin **(Cum3)**; 6-hydroxy-7-(5′-hydroxy-3′,7′-dimethylocta-2′,6′-dien)-oxycoumarin **(Cum6)**; 6-hydroxy-7-(7′-hydroxy-3′,7′-dimethylocta-2′,5′-dien)-oxycoumarin **(Cum7)**; umbelliferone **(Cum14)**; *O*-prenylumbelliferone **(Cum15)**	*In vitro* inhibition of soybean 15-lipoxygenase (15-sLOX)	Torres et al. (2013)
	*H. remyanus*	resin	extract	Inhibition of arachidonic acid-induced ear edema in mice	Faini et al. (2011)
	*H. taeda*	-	extract (EtOH); taedol **(Mon41)**; 18-acetoxy-*cis*-cleroda-3,13(*E*)-dien-15-oic acid **(Dit99)**; sakuranetin **(Flv67)**	Inhibition of arachidonic acid-induced ear edema in mice	Faini et al. (2008)
Antioxidant	*H. baylahuen*	commercial product (herbal tea)	infusion	Antioxidant capacity *in vitro* (ORAC, TEAC-ABTS, HClO quenching and ONOO^−^quenching assays)	Speisky et al., 2006; Alarcón et al., 2008
		aerial parts	infusion, extract (MeOH)	Antioxidant capacity *in vitro* (DPPH assay)	Schmeda-Hirschmann et al. (2015)
		leaves	infusion, extract (MeOH, H_2_O/EtOH, EtOH), resin	Antioxidant capacity *in vitro* (DPPH assay)	Vogel et al., 2005a; Méttola et al., 2018; Elgueta et al., 2021
	*H. deserticola*	aerial parts	infusion, extract (MeOH)	Antioxidant capacity *in vitro* (DPPH assay)	Schmeda-Hirschmann et al. (2015)
	*H. multifolius*	aerial parts	infusion, extract (MeOH)	Antioxidant capacity *in vitro* (DPPH assay)	Schmeda-Hirschmann et al. (2015)
		aerial parts	quercetin **(Flv1)**; isorhamnetin **(Flv18)**; prenyletin **(Cum3)**; haplopinol **(Cum4)**; 6-hydroxy-7-(5′-hydroxy-3′,7′-dimethylocta-2′,6′-dien)-oxycoumarin **(Cum6)**; 6-hydroxy-7-(7′-hydroxy-3′,7′-dimethylocta-2′,5′-dien)-oxycoumarin **(Cum7)**; 6-hydroxy-7-[(*E*,*E*)-3′,7′-dimethyl-2′,4′,7′-octatrienyloxy] coumarin **(Cum8)**	Antioxidant capacity *in vitro* (DPPH assay)	Torres et al. (2006)
		leaves	infusion, extract (MeOH), resin	Antioxidant capacity *in vitro* (DPPH assay)	Vogel et al. (2005a)
	*H. remyanus*	leaves	infusion, extract (MeOH), resin	Antioxidant capacity *in vitro* (DPPH assay)	Vogel et al. (2005b)
	*H. rigidus*	aerial parts	sternbin **(Flv70)**	Antioxidant capacity *in vitro* (TEAC – ABTS, DPPH assay)	Morales et al. (2009)
		aerial parts	infusion, extract (MeOH)	Antioxidant capacity *in vitro* (DPPH assay)	Schmeda-Hirschmann et al. (2015)
	*H. taeda*	resin, aerial parts	9-*trans*-*p*-coumaroyloxy-*α*-terpineol **(Cin7)**; 7-*trans*-*p*-coumaroyloxy-taedol **(Cin8)**	Antioxidant capacity *in vitro* (DPPH assay)	Faini et al. (2007)
		aerial parts	infusion; extract (MeOH)	Antioxidant capacity *in vitro* (DPPH assay)	Schmeda-Hirschmann et al. (2015)
		leaves	infusion, extract (MeOH), resin	Antioxidant capacity *in vitro* (DPPH assay)	Vogel et al. (2005b)
Antitumoral	*H. remyanus*	resin	extract (CH_2_Cl_2_)	Cytotoxic effect against T-lymphoblastic leukemia cell line (CCRF-CEM)	Faini et al. (2011)
	*H. rigidus*	aerial parts	rigidusol **(Dit100)**	Cytotoxic effect against human breast adenocarcinoma cell line (MCF-7)	Morales et al., 2000a; Vogel et al., 2005a
			sternbin **(Flv70)**	Cytotoxic effect against human breast adenocarcinoma (MCF-7), human lung carcinoma (A-549) and human colon adenocarcinoma (HT–29) cell lines	Morales et al. (2009)
Diuretic	*H. baylahuen*	leaves	extract (EtOH)	Diuretic effect on Wistar rats	Méttola et al. (2018)
Hepatoprotective	*H. baylahuen*	aerial parts	infusion; 7-*O*-methylaromadenrin **(Flv76)**	Decrease of glutamic pyruvic transaminase (GTP) levels in serum of rats under CCl_4_-induced liver injury	Nuñez-Alarcon et al. (1993)
			infusion	Reduction of serum bilirubin concentration, bromosulfophthalein and alanine aminotransferase activity in dogs under CCl_4_-induced liver injury	Martin et al. (1988)
Inhibitory of lipid peroxidation	*H. baylahuen*	leaves	infusion; extracts (MeOH, EtOH)	Inhibition of lipid peroxidation *in vitro* and in erythrocyte membranes	Vogel et al., 2005; Méttola et al., 2018
	*H. multifolius*	leaves	infusion; extract (MeOH)	Inhibition of lipid peroxidation in erythrocyte membranes	Vogel et al. (2005a)
	*H. remyanus*	leaves	infusion; extract (MeOH)	Inhibition of lipid peroxidation in erythrocyte membranes	Vogel et al. (2005a)
	*H. rigidus*	aerial parts	sternbin **(Flv70)**	Inhibition of iron/ascorbate-induced lipid peroxidation in rat cells	Morales et al. (2009)
	*H. taeda*	leaves	infusion; extract (MeOH)	Inhibition of lipid peroxidation in erythrocyte membranes	Vogel et al. (2005a)
Muscle relaxant	*H. rigidus*	aerial parts	extracts (H_2_O, MeOH, CH_2_Cl_2_)	Relaxation of L-phenylephrine precontracted *corpus cavernosum* smooth muscles of Guinea pigs	Hnatyszyn et al. (2003)
Inhibition of GLUT1 transporter	*H. baylahuen*	leaves	rhamnetin **(Flv17)**	Inhibition of GLUT1 transporter in human myeloid HL-60 cells, in transfected Chinese hamster ovary cells overexpressing GLUT1, and in normal human erythrocytes; inhibition of binding of cytochalasin B to GLUT1 in erythrocyte ghosts	Vera et al. (2001)
			isorhamnetin **(Flv18)**		

#### 5.2.1 *H. anthylloides* meyen & walp

Although the bioactivity of the species *H. anthylloides* has not been extensively studied, it is reported that dichloromethane extracts of its resinous exudates present antibacterial effects, inhibiting the *in vitro* growth of several human pathogenic bacteria (Urzúa et al., 1995a).

#### 5.2.2 *H. baylahuen* remy

*Haplopappus baylahuen* is the species with the highest number of bioactivity studies. Extracts and decoctions of its aerial parts are reported to have antibacterial and bactericide effects against *Staphylococcus aureus*, *Bacillus subtilis*, *Acremonium falciforme* (Lazo, 1990) and *Salmonella enteritidis* (Elgueta et al., 2021). Moreover, emulsions of its resin have been successfully used to treat the symptoms of dysentery in affected individuals (Fingland, 1903), while extracts of the aerial parts of *H. baylahuen* have shown anti-inflammatory (Adzet and Gene, 1991), diuretic (Méttola et al., 2018) and hepatoprotective (Nuñez-Alarcon et al., 1993) effects in rat models and hepatoprotective activity in dog models (Martin et al., 1988). The hepatoprotective effect in rats under CCl_4_-induced liver injury has also been confirmed in the case of 7-*O*-methylaromadenrin **(Flv76)** isolated from the aerial parts of the plant (Nuñez-Alarcon et al., 1993). Moreover, rhamnetin **(Flv17)** and isorhamnetin **(Flv18)** isolated from the leaves of *H. baylahuen* have been found to inhibit in a dose-dependent manner the glucose transporter GLUT1 in human cell lines and *in vivo* in hamsters (Vera et al., 2001). Finally, extracts of the aerial parts of this species have demonstrated significant antioxidant capacity as measured by various *in vitro* assays (Vogel et al., 2005a; Speisky et al., 2006; Alarcón et al., 2008; Schmeda-Hirschmann et al., 2015; Méttola et al., 2018; Elgueta et al., 2021), while also inhibiting lipid peroxidation *in vitro* and in erythrocyte membranes (Vogel et al., 2005a; Méttola et al., 2018).

#### 5.2.3 *H. chrysanthemifolius* (Less.) DC

In the case of *H. chrysanthemifolius*, scientific evidence supports the antibacterial effect of the methanolic extracts of its resinous exudates, as this has been demonstrated through the *in vitro* growth inhibition of several Gram-positive human pathogenic bacterial strains (Urzúa et al., 2004b; 2012).

#### 5.2.4 *H. deserticola* Phil

The diterpene 18-acetoxy-*cis*-cleroda-3,13(*E*)-dien-15-oic acid **(Dit99)** isolated from the resin of *H. deserticola* presented a bactericidal effect against *Streptococcus mutans* (Urzúa Moll et al., 1997), while the *in vitro* antioxidant capacity of the infusion and methanolic extract of the plant’s aerial parts has also been documented (Schmeda-Hirschmann et al., 2015).

#### 5.2.5 *H. diplopappus* Remy subsp. *diplopappus*

The resin of *H. diplopappus* subsp. *diplopappus*, as well as the isolated diterpenoid 13-*O*-*β-*xylopyranosyl-*ent*-manool **(Dit8)** present antibacterial effect against various Gram-positive and Gram-negative human pathogenic bacteria (Urzúa et al., 1995a).

#### 5.2.6 *H. foliosus* (Hook. & Arn.) Hook. & Arn

Scientific evidence supports the antibacterial effect of the resinous exudate of *H. foliosus* against several Gram-positive and Gram-negative human pathogenic bacteria (Urzúa et al., 1995a; 2003; Urzúa and Mendoza, 2001). Similar bioactivity has been attributed to the diterpenes 2*α*-hydroxy-*cis*-clero-3,13(*Z*),8(17)-trien-15-oic acid **(Dit87)** and 2*α*-acetoxy-*cis*-clero-3,13(*Z*),8(17)-trien-15-oic acid **(Dit88)** which were isolated from the resin of *H. foliosus* (Urzúa et al., 2003).

#### 5.2.7 *H. litoralis* Phil

In the case of *H. litoralis,* it has been reported that its resinous exudate inhibits the *in vitro* growth of *Bacillus cereus, B. subtilis, Enterococcus faecalis, Listeria monocytogens, Micrococcus luteus, S. aureus* (Urzúa et al., 2004b; 2012).

#### 5.2.8 *H. multifolius* reiche

Scientific literature provides evidence that support the antibacterial effect of *H. multifolius* resin and aerial parts extracts against a wide spectrum of Gram-positive and Gram-negative human pathogenic bacteria (Urzúa et al., 1995a; Padilla et al., 2021). Moreover, similar antibacterial activity has been documented for the coumarins esculetin **(Cum1)**, prenyletin **(Cum3)** and haplopinol **(Cum4)** isolated from the aerial parts of this species (Chiang et al., 1982). Regarding the *in vitro* antioxidant capacity of *H. multifolius*, this has been demonstrated in the case of extracts, aerial parts infusions and resin (Vogel et al., 2005a; Schmeda-Hirschmann et al., 2015), as well as for the isolated compounds quercetin **(Flv1)**, isorhamnetin **(Flv18)**, prenyletin **(Cum3)**, haplopinol **(Cum4)**, 6-hydroxy-7-(5′-hydroxy-3′,7′-dimethylocta-2′,6′-dien)-oxycoumarin **(Cum6)**, 6-hydroxy-7-(7′-hydroxy-3′,7′-dimethylocta-2′,5′-dien)-oxycoumarin **(Cum7)** and 6-hydroxy-7-[(E,E)-3′,7′-dimethyl-2′,4′,7′-octatrienyloxy] coumarin **(Cum8)** (Torres et al., 2006). Furthermore, the isolated compounds esculetin **(Cum1)**, esculin **(Cum2)**, prenyletin **(Cum3)**, 6-hydroxy-7-(5′-hydroxy-3′,7′-dimethylocta-2′,6′-dien)-oxycoumarin **(Cum6)**, 6-hydroxy-7-(7′-hydroxy-3′,7′-dimethylocta-2′,5′-dien)-oxycoumarin **(Cum7)**, umbelliferone **(Cum14)** and *O*-prenylumbelliferone **(Cum15)** have demonstrated an anti-inflammatory effect associated to the *in vitro* inhibition of soybean 15-lipoxygenase (Torres et al., 2013). Finally, methanolic extracts and infusions of *H. multifolius* leaves inhibited the lipid peroxidation in erythrocyte membranes (Vogel et al., 2005a).

#### 5.2.9 *H. remyanus* wedd

Infusions, methanolic extracts and resin from the leaves of *H. remyanus* demonstrated a significant antioxidant capacity *in vitro*, while also inhibiting lipid peroxidation in erythrocyte membranes (Vogel et al., 2005a). Furthermore, the resinous exudates of the plant exhibited an anti-inflammatory effect in mice (Faini et al., 2011) and a cytotoxic effect against T-lymphoblastic leukemia cell line (CCRF-CEM) (Faini et al., 2011).

#### 5.2.10 *H. rigidus* Phil

Extracts of the aerial parts of *H. rigidus* have effectively inhibited the *in vitro* growth of several Gram-positive bacterial strains (Morales et al., 2003; Ortiz et al., 2019), presented a significant *in vitro* antioxidant capacity (Schmeda-Hirschmann et al., 2015) and also acted as muscle relaxants in Guinea pig models (Hnatyszyn et al., 2003). The isolated flavanone sternbin **(Flv70)** presented high *in vitro* antioxidant capacity, lipid peroxidation inhibitory effects in rat cells and also antitumoral effect against the human breast adenocarcinoma (MCF-7), human lung carcinoma (A-549) and human colon adenocarcinoma (HT–29) cell lines (Morales et al., 2009). The isolated diterpene rigidusol **(Dit100)** also had a cytotoxic effect on human breast adenocarcinoma cells line (MCF-7) (Morales et al., 2000b).

#### 5.2.11 *H. schumannii* (Kuntze) G.K. Br. & W.D. Clark

The resinous exudates of *H. schumannii* inhibited the *in vitro* growth of several Gram-positive bacterial human pathogens (Urzúa et al., 1995a).

#### 5.2.12 *H. scrobiculatus* (Nees) DC

Similarly, the only known bioactivity regarding the resin of *H. scrobiculatus* is that of the *in vitro* antibacterial effect against several Gram-positive bacteria (Urzúa et al., 1995a; 2004b; 2012).

#### 5.2.13 *H. taeda* reiche

Ethanolic extracts and infusions of aerial parts of *H. taeda* successfully inhibited the *in vitro* growth of several *Bacillus* and *Staphylococcus* bacterial strains (Padilla et al., 2021). Regarding the *in vitro* antioxidant capacity of the species, this has been shown to be significant in the case of aerial parts infusions, extracts and resinous exudates (Vogel et al., 2005a; Schmeda-Hirschmann et al., 2015), as well as for the isolated compounds 9-*trans*-*p*-coumaroyloxy-*α*-terpineol **(Cin7)** and 7-*trans*-*p*-coumaroyloxy-taedol **(Cin8)** (Faini et al., 2007). Moreover, leaf infusions and methanolic extracts of *H. taeda* inhibited lipid peroxidation in erythrocyte membranes (Vogel et al., 2005a). Ethanolic extracts, as well as the isolated compounds taedol **(Mon41)**, 18-acetoxy-*cis*-cleroda-3,13(*E*)-dien-15-oic acid **(Dit99)**, and sakuranetin **(Flv67)** exhibited an anti-inflammatory effect against arachidonic acid-induced ear edema in mice (Faini et al., 2008).

#### 5.2.14 *H. uncinatus* Phil

Extracts of the aerial parts and resinous exudates of *H. uncinatus*, as well as the isolated diterpenoid 18-acetoxy-*cis*-cleroda-3-en-15-oic acid (10*βH*, 16*ξ,* 19*β*, 17*β*, 20*α* form) **(Dit84)** have been reported to inhibit *in vitro* the growth of various Gram-positive human pathogenic bacteria (Urzúa et al., 1995a; 2006; Urzúa and Mendoza, 2001).

#### 5.2.15 *H. velutinus* remy, *H. velutinus* Remy subsp. *illinitus* (Phil.) Klingenb

Dichloromethane extracts of the resinous exudates of *H. velutinus* and its subspecies H*. velutinus* subsp. *illinitus* inhibited *in vitro* the growth of various Gram-positive and Gram-negative human pathogenic bacteria (Urzúa et al., 1995a).

## 6 Non-human health related bioactivity and toxicity

Among the pharmacological activities attributed to *Haplopappus* species and not related to the human health, the most studied is the antimicrobial effect against plant pathogens. The essential oil of the leaves of *H. baylahuen* inhibited the *in vitro* growth of the fungi *Aspergillus nigra* and *Fusarium oxysporum* (Becerra et al., 2010). Moreover, the diterpenoid 7,13-(*E*)-labdadien-15,18-dioic acid 18-methyl ester **(Dit45)** was isolated from the resinous exudate of *Haplopappus velutinus* and inhibiting *in vitro* the mycelial growth of *Botrytis cinerea* (Echeverría et al., 2019). In the case of the phytopathogenic bacterium *Clavibacter michiganensis* subsp. *michiganensis,* its *in vitro* growth was inhibited by the resin (Urzúa and Mendoza, 2001) and the isolated diterpene 18-acetoxy-*cis*-cleroda-3-en-15-oic acid (10*βH*, 16*ξ,* 19*β*, 17*β*, 20*α* form) **(Dit84)** (Urzúa et al., 2006) from *H. uncinatus*, as well as by the methanolic extract of the resin of *H. foliosus* (Urzúa and Mendoza, 2001).

The essential oil of *H. foliosus* also exhibited insecticide effects against house flies (*Musca domestica*) (Urzúa et al., 2010), while hydroethanolic and chloroform extracts of *H. rigidus* presented biotoxic activity against *Artemia salina* (Morales et al., 2003).

## 7 Concluding remarks and future perspectives

The available scientific literature on the genus *Haplopappus* can be said to support, although partially, its widespread and longstanding use as a medicinal plant. However, the results of the present review highlight several limitations that need to be addressed.

Firstly, phytochemical and bioactivity research of the genus *Haplopappus* is largely concentrated in the 1990s and 2000s, with almost 80% of the investigation having been performed before 2010. Therefore, a revival of scientific interest and the application of modern, more advanced and diverse analytical and biological techniques can further elucidate the composition and bioactivity of *Haplopappus* plant species, thus broadening the existing knowledge and promoting its potential uses.

Furthermore, phytochemical and pharmacological evidence is available only for the 40% and 23%, respectively, of the botanical taxa of the genus *Haplopappus*, while for many of the studied taxa, the available information is rather limited. Similarly, terpenoids and phenolics correspond to approximately 70% of the compounds reported in *Haplopappus* spp., suggesting that scientific investigation up to date has possibly understudied other chemical groups. It is, therefore, suggested to extend the focus of scientific research to more, if not all, *Haplopappus* species and groups of chemical compounds, thus permitting to fully explore its promising chemical and biological prospects.

Based on the available bioactivity and pharmacological evidence, *Haplopappus* species can be considered as a valuable plant resource for health-promoting applications. However, the majority of the investigation provides evidence associated to the *in vitro* antibacterial and antioxidant activity of the genus *Haplopappus.* In contrast, there is a lack of scientific evidence to support or refute various traditional uses, while, at the same time, the limited number of *in vivo* studies and/or clinical trials hinders its wider human health-promoting application and secure use.

In this context, the information presented in the present review supports the ethnopharmacological, phytochemical and bioactive potential of the genus *Haplopappus*, while addressing the aforementioned limitations could further promote and broaden both scientific research and future applications and uses.
