# Supplementary material for: The genus Haplopappus: botany, phytochemistry, traditional uses, and pharmacological properties
Source: Front Pharmacol. 2024 Oct 21;15:1490243. doi: 10.3389/fphar.2024.1490243 (PMC11532054; doi:10.3389/fphar.2024.1490243)
Supplement: Supplementary file 1 [file Table1.DOCX]

**Table S1.** Chemical compounds reported in the species of the genus *Haplopappus*.

| **No.** | **Compounds** | **Plant name(s)** | **Plant part(s)** | **References** |
| --- | --- | --- | --- | --- |
| **Alkanes** | | | | |
| **Ala1** | decane (C_10_H_22_) | *H. chrysanthemifolius* | flower heads | (Urzúa et al. 2007b) |
| **Ala2** | undecane (C_11_H_24_) | *H. bustillosianus* | aerial parts | (Urzúa et al. 2007a) |
|  |  | *H. chrysanthemifolius* | flower heads | (Urzúa et al. 2007b) |
| **Ala3** | dodecane (C_12_H_26_) | *H. bustillosianus* | aerial parts | (Urzúa et al. 2007a) |
|  |  | *H. chrysanthemifolius* | flower heads | (Urzúa et al. 2007b) |
|  |  | *H. foliosus* | aerial parts | (Urzúa 2004) |
| **Ala4** | tridecane (C_13_H_28_) | *H. bustillosianus* | aerial parts | (Urzúa et al. 2007a) |
|  |  | *H. chrysanthemifolius* | flower heads | (Urzúa et al. 2007b) |
| **Ala5** | tetradecane (C_14_H_30_) | *H. bustillosianus* | aerial parts | (Urzúa et al. 2007a) |
|  |  | *H. chrysanthemifolius* | flower heads | (Urzúa et al. 2007b) |
|  |  | *H. foliosus* | aerial parts | (Urzúa 2004) |
| **Ala6** | pentadecane (C_15_H_32_) | *H. chrysanthemifolius* | flower heads | (Urzúa et al. 2007b) |
| **Ala7** | hexadecane (C_16_H_34_) | *H. bustillosianus* | aerial parts | (Urzúa et al. 2007a) |
|  |  | *H. chrysanthemifolius* | flower heads | (Urzúa et al. 2007b) |
|  |  | *H. foliosus* | aerial parts | (Urzúa 2004) |
| **Ala8** | heptadecane (C_17_H_36_) | *H. bustillosianus* | aerial parts | (Urzúa et al. 2007a) |
|  |  | *H. chrysanthemifolius* | flower heads | (Urzúa et al. 2007b) |
| **Ala9** | octadecane (C_18_H_38_) | *H. bustillosianus* | aerial parts | (Urzúa et al. 2007a) |
|  |  | *H. chrysanthemifolius* | flower heads | (Urzúa et al. 2007b) |
|  |  | *H. foliosus* | aerial parts | (Urzúa 2004) |
| **Ala10** | nonadecane (C_19_H_40_) | *H. bustillosianus* | aerial parts | (Urzúa et al. 2007a) |
|  |  | *H. chrysanthemifolius* | flower heads | (Urzúa et al. 2007b) |
| **Ala11** | eicosane (C_20_H_42_) | *H. baylahuen* | essential oil (leaves) | (Becerra et al. 2010) |
|  |  | *H. bustillosianus* | aerial parts | (Urzúa et al. 2007a) |
| **Ala12** | heneicosane (C_21_H_44_) | *H. bustillosianus* | aerial parts | (Urzúa et al. 2007a) |
|  |  | *H. chrysanthemifolius* | flower heads | (Urzúa et al. 2007b) |
| **Ala13** | docosane (C_22_H_46_) | *H. bustillosianus* | aerial parts | (Urzúa et al. 2007a) |
|  |  | *H. chrysanthemifolius* | flower heads | (Urzúa et al. 2007b) |
| **Ala14** | tricosane (C_23_H_48_) | *H. bustillosianus* | aerial parts | (Urzúa et al. 2007a) |
|  |  | *H. chrysanthemifolius* | flower heads | (Urzúa et al. 2007b) |
|  |  | *H. foliosus* | resin, aerial parts | (Urzúa et al. 2000; Urzúa 2004) |
|  |  | *H. schumannii* | aerial parts | (Urzúa et al. 2004a) |
|  |  | *H. uncinatus* | aerial parts | (Urzúa et al. 2004a, 2006) |
|  |  | *H. velutinus* | aerial parts | (Urzúa et al. 2004a) |
|  |  | *H. velutinus* subsp. *illinitus* | aerial parts | (Urzúa et al. 2004a) |
| **Ala15** | tetracosane (C_24_H_50_) | *H. bustillosianus* | aerial parts | (Urzúa et al. 2007a) |
|  |  | *H. chrysanthemifolius* | flower heads | (Urzúa et al. 2007b) |
|  |  | *H. foliosus* | resin | (Urzúa et al. 2000) |
|  |  | *H. schumannii* | aerial parts | (Urzúa et al. 2004a) |
|  |  | *H. uncinatus* | aerial parts | (Urzúa et al. 2004a, 2006) |
|  |  | *H. velutinus* | aerial parts | (Urzúa et al. 2004a) |
|  |  | *H. velutinus* subsp. *illinitus* | aerial parts | (Urzúa et al. 2004a) |
| **Ala16** | pentacosane (C_25_H_52_) | *H. bustillosianus* | aerial parts | (Urzúa et al. 2007a) |
|  |  | *H. chrysanthemifolius* | flower heads | (Urzúa et al. 2007b) |
|  |  | *H. foliosus* | resin, aerial parts | (Urzúa et al. 2000; Urzúa 2004) |
|  |  | *H. schumannii* | aerial parts | (Urzúa et al. 2004a) |
|  |  | *H. uncinatus* | resin, aerial parts | (Urzúa et al. 2000, 2004a, 2006) |
|  |  | *H. velutinus* | aerial parts | (Urzúa et al. 2004a) |
|  |  | *H. velutinus* subsp. *illinitus* | aerial parts | (Urzúa et al. 2004a) |
| **Ala17** | hexacosane (C_26_H_54_) | *H. bustillosianus* | aerial parts | (Urzúa et al. 2007a) |
|  |  | *H. chrysanthemifolius* | flower heads | (Urzúa et al. 2007b) |
|  |  | *H. foliosus* | resin, aerial parts | (Urzúa et al. 2000; Urzúa 2004) |
|  |  | *H. schumannii* | aerial parts | (Urzúa et al. 2004a) |
|  |  | *H. uncinatus* | aerial parts | (Urzúa et al. 2004a) |
|  |  | *H. velutinus* | aerial parts | (Urzúa et al. 2004a) |
|  |  | *H. velutinus* subsp. *illinitus* | aerial parts | (Urzúa et al. 2004a) |
| **Ala18** | heptacosane (C_27_H_56_) | *H. bustillosianus* | aerial parts | (Urzúa et al. 2007a) |
|  |  | *H. chrysanthemifolius* | flower heads | (Urzúa et al. 2007b) |
|  |  | *H. foliosus* | resin, aerial parts | (Urzúa et al. 2000; Urzúa 2004) |
|  |  | *H. schumannii* | aerial parts | (Urzúa et al. 2004a) |
|  |  | *H. uncinatus* | resin, aerial parts | (Urzúa et al. 2000, 2004a, 2006) |
|  |  | *H. velutinus* | aerial parts | (Urzúa et al. 2004a) |
|  |  | *H. velutinus* subsp. *illinitus* | aerial parts | (Urzúa et al. 2004a) |
| **Ala19** | octacosane (C_28_H_58_) | *H. bustillosianus* | aerial parts | (Urzúa et al. 2007a) |
|  |  | *H. chrysanthemifolius* | flower heads | (Urzúa et al. 2007b) |
|  |  | *H. foliosus* | resin, aerial parts | (Urzúa et al. 2000; Urzúa 2004) |
|  |  | *H. schumannii* | aerial parts | (Urzúa et al. 2004a) |
|  |  | *H. uncinatus* | aerial parts | (Urzúa et al. 2004a, 2006) |
|  |  | *H. velutinus* | aerial parts | (Urzúa et al. 2004a) |
|  |  | *H. velutinus* subsp. *illinitus* | aerial parts | (Urzúa et al. 2004a) |
| **Ala20** | nonacosane (C_29_H_60_) | *H. bustillosianus* | aerial parts | (Urzúa et al. 2007a) |
|  |  | *H. chrysanthemifolius* | flower heads | (Urzúa et al. 2007b) |
|  |  | *H. foliosus* | resin, aerial parts | (Urzúa et al. 2000; Urzúa 2004) |
|  |  | *H. schumannii* | aerial parts | (Urzúa et al. 2004a) |
|  |  | *H. uncinatus* | resin, aerial parts | (Urzúa et al. 2000, 2004a, 2006) |
|  |  | *H. velutinus* | aerial parts | (Urzúa et al. 2004a) |
|  |  | *H. velutinus* subsp. *illinitus* | aerial parts | (Urzúa et al. 2004a) |
| **Ala21** | triacontane (C_30_H_62_) | *H. bustillosianus* | aerial parts | (Urzúa et al. 2007a) |
|  |  | *H. chrysanthemifolius* | flower heads | (Urzúa et al. 2007b) |
|  |  | *H. foliosus* | resin, aerial parts | (Urzúa et al. 2000; Urzúa 2004) |
|  |  | *H. schumannii* | aerial parts | (Urzúa et al. 2004a) |
|  |  | *H. uncinatus* | aerial parts | (Urzúa et al. 2004a, 2006) |
|  |  | *H. velutinus* | aerial parts | (Urzúa et al. 2004a) |
|  |  | *H. velutinus* subsp. *illinitus* | aerial parts | (Urzúa et al. 2004a) |
| **Ala22** | hentriacontane (C_31_H_64_) | *H. angustifolius* | aerial parts | (Silva and Sammes 1973) |
|  |  | *H. bustillosianus* | aerial parts | (Urzúa et al. 2007a) |
|  |  | *H. chrysanthemifolius* | flower heads | (Urzúa et al. 2007b) |
|  |  | *H. foliosus* | resin, aerial parts | (Silva and Sammes 1973; Urzúa et al. 2000; Urzúa 2004) |
|  |  | *H. schumannii* | aerial parts | (Urzúa et al. 2004a) |
|  |  | *H. uncinatus* | resin, aerial parts | (Urzúa et al. 2000, 2004a, 2006) |
|  |  | *H. velutinus* | aerial parts | (Urzúa et al. 2004a) |
|  |  | *H. velutinus* subsp. *illinitus* | aerial parts | (Urzúa et al. 2004a) |
| **Ala23** | dotriacontane (C_32_H_66_) | *H. bustillosianus* | aerial parts | (Urzúa et al. 2007a) |
|  |  | *H. chrysanthemifolius* | flower heads | (Urzúa et al. 2007b) |
|  |  | *H. foliosus* | resin, aerial parts | (Urzúa et al. 2000; Urzúa 2004) |
| **Ala24** | tritriacontane (C_33_H_68_) | *H. bustillosianus* | aerial parts | (Urzúa et al. 2007a) |
|  |  | *H. chrysanthemifolius* | flower heads | (Urzúa et al. 2007b) |
|  |  | *H. foliosus* | resin, aerial parts | (Urzúa et al. 2000; Urzúa 2004) |
|  |  | *H. schumannii* | aerial parts | (Urzúa et al. 2004a) |
|  |  | *H. uncinatus* | resin, aerial parts | (Urzúa et al. 2000, 2004a, 2006) |
|  |  | *H. velutinus* | aerial parts | (Urzúa et al. 2004a) |
|  |  | *H. velutinus* subsp. *illinitus* | aerial parts | (Urzúa et al. 2004a) |
| **Ala25** | 2-methyldecalin | *H. chrysanthemifolius* | flower heads | (Urzúa et al. 2007b) |
| **Ala26** | 2,4,6-trimethyloctane | *H. chrysanthemifolius* | flower heads | (Urzúa et al. 2007b) |
| **Ala27** | 2,6­dimethylundecane | *H. chrysanthemifolius* | flower heads | (Urzúa et al. 2007b) |
| **Ala28** | 4,6­dimethylundecane | *H. chrysanthemifolius* | flower heads | (Urzúa et al. 2007b) |
| **Ala29** | 2,10­dimethylundecane | *H. chrysanthemifolius* | flower heads | (Urzúa et al. 2007b) |
| **Alkenes** | | | | |
| **Ale1** | 11-tricosene (C_23_H_46_) | *H. foliosus* | aerial parts | (Urzúa 2004) |
| **Ale2** | 3-ethyl-1,4-hexadiene | *H. velutinus* | aerial parts | (Urzúa et al. 2004a) |
| **Ale3** | 2,7-dimethyl-5-(1-methylethenyl)-1,8-nonadiene | *H. uncinatus* | resin, aerial parts | (Urzúa et al. 2004a) |
| **Ale4** | 3,3,5,5-tetramethylcyclopentene | *H. velutinus* subsp. *illinitus* | aerial parts | (Urzúa et al. 2004a) |
| **Alkynes** | | | | |
| **Aly1** | 1-octadecyne (C_18_H_34_) | *H. schumannii* | aerial parts | (Urzúa et al. 2004a) |
| **Alcohols** | | | | |
| **Alc1** | hexacosanol | *H. angustifolius* | aerial parts | (Silva and Sammes 1973) |
|  |  | *H. foliosus* | aerial parts | (Silva and Sammes 1973) |
| **Alc2** | ethylresorcinol | *H. foliosus* | aerial parts | (Urzúa 2004) |
| **Alc3** | 2-nonyn-1-ol | *H. velutinus* | aerial parts | (Urzúa et al. 2004a) |
| **Alc4** | 2-pentadecen-1-ol | *H. velutinus* | aerial parts | (Urzúa et al. 2004a) |
| **Alc5** | *n*-dodecenyl-1-ol | *H. velutinus* | aerial parts | (Urzúa et al. 2004a) |
| **Ethers** | | | | |
| **Eth1** | diisopropyl ether | *H. foliosus* | aerial parts | (Urzúa 2004) |
| **Aromatic hydrocarbons and derivatives** | | | | |
| **Arh1** | benzene | *H. baylahuen* | essential oil (leaves) | (Becerra et al. 2010) |
| **Arh2** | azulene | *H. baylahuen* | essential oil (leaves) | (Becerra et al. 2010) |
| **Arh3** | *α*-asarone | *H. foliosus* | aerial parts | (Urzúa 2004) |
| **Arh4** | naphthalene | *H. baylahuen* | essential oil (leaves) | (Becerra et al. 2010) |
| **Arh5** | 1,2,3,4,5,6,7,8­octahydro­1­methylphenantrene | *H. foliosus* | aerial parts | (Urzúa 2004) |
| **Arh6** | eugenol | *H. foliosus* | aerial parts | (Urzúa 2004) |
| **Arh7** | styrene | *H. foliosus* | stems | (Villagra et al. 2021) |
| **Arh8** | safrol | *H. foliosus* | stems | (Villagra et al. 2021) |
| **Arh9** | elemicin | *H. foliosus* | aerial parts | (Urzúa 2004) |
| **Arh10** | dihydrobenzofuran | *H. foliosus* | resin, aerial parts | (Urzúa et al. 2000; Urzúa 2004) |
| **Aldehydes** | | | | |
| **Ald1** | benzaldehyde | *H. foliosus* | aerial parts, stems | (Urzúa 2004; Villagra et al. 2021) |
| **Ald2** | 2,3­dichloro­2­methylpropanal | *H. foliosus* | aerial parts | (Urzúa 2004) |
| **Ald3** | *trans*-2-hexenal | *H. foliosus* | essential oil | (Urzúa et al. 2010) |
| **Ald4** | nonanal | *H. foliosus* | stems | (Villagra et al. 2021) |
| **Ald5** | decanal | *H. foliosus* | stems | (Villagra et al. 2021) |
| **Ald6** | 3-ethylbenzaldehyde | *H. foliosus* | stems | (Villagra et al. 2021) |
| **Ald7** | 4-vinylbenzaldehyde | *H. foliosus* | aerial parts | (Urzúa 2004) |
| **Ald8** | vanillin | *H. velutinus* | stems | (Marambio and Silva 1996) |
| **Ketones** | | | | |
| **Ket1** | 3-hydroxyacetophenone | *H. foliosus* | aerial parts | (Urzúa 2004) |
|  |  | *H. bustillosianus* | aerial parts | (Urzúa et al. 2007a) |
| **Ket2** | 4-hydroxyacetophenone | *H. anthylloides* | aerial parts | (Zdero et al. 1990) |
|  |  | *H. arbutoides* | aerial parts | (Rossomando et al. 1995) |
|  |  | *H. glutinosus* | aerial parts | (Jakupovic et al. 1986) |
|  |  | *H. paucidentatus* | aerial parts | (Jakupovic et al. 1986) |
| **Ket3** | 3-ethylacetophenone | *H. foliosus* | stems | (Villagra et al. 2021) |
| **Ket4** | 4-ethylacetophenone | *H. foliosus* | stems | (Villagra et al. 2021) |
| **Ket5** | picein | *H. velutinus* | stems | (Marambio and Silva 1996) |
| **Ket6** | dihydro-*α*-ionone | *H. foliosus* | aerial parts | (Urzúa 2004) |
|  |  | *H. schumannii* | aerial parts | (Urzúa et al. 2004a) |
| **Ket7** | 4,4­dimethyl­2­allylcyclohexanone | *H. foliosus* | aerial parts | (Urzúa 2004) |
| **Esters** | | | | |
| **Est1** | methyl octanoate | *H. velutinus* subsp. *illinitus* | aerial parts | (Urzúa et al. 2004a) |
| **Est2** | 5-methyl-octanoic acid methyl ester | *H. velutinus* subsp. *illinitus* | aerial parts | (Urzúa et al. 2004a) |
| **Est3** | benzenepropanoic acid, 2-methyl-6-methylene-2,7-octadienyl ester | *H. remyanus* | aerial parts | (Zdero et al. 1991a) |
| **Est4** | (±)-1-acetoxy-2-(*p*-tolyl)-2-propanol | *H. remyanus* | aerial parts | (Zdero et al. 1991a) |
| **Est5** | 2-hydroxy-2-(4-methylphenyl)propyl benzenepropanoate | *H. remyanus* | aerial parts | (Zdero et al. 1991a) |
| **Est6** | 2-hydroxy-2-(4-methyl-3-cyclohexen-1-yl)propyl benzenepropanoate | *H. remyanus* | aerial parts | (Zdero et al. 1991a) |
| **Est7** | 2-hydroxy-2-(4-methyl-3-cyclohexen-1-yl)propyl 3-phenyl-2-propenoate | *H. remyanus* | aerial parts | (Zdero et al. 1991a) |
| **Est8** | (*Z*)-3-hexenyl acetate | *H. foliosus* | stems | (Villagra et al. 2021) |
| **Lactones** | | | | |
| **Ltn1** | lavender lactone | *H. velutinus* | aerial parts | (Urzúa et al. 2004a) |
| **Ltn2** | tetrahydroactinidiolide | *H. foliosus* | aerial parts | (Urzúa 2004) |
| **Furanones** | | | | |
| **Fur1** | 5,5-dimethyl-2(5*H*)-furanone | *H. velutinus* | aerial parts | (Urzúa et al. 2004a) |
|  |  | *H. velutinus* subsp. *illinitus* | aerial parts | (Urzúa et al. 2004a) |
|  |  | *H. schumannii* | aerial parts | (Urzúa et al. 2004a) |
| **Lactams** | | | | |
| **Ltm1** | 4-phenyl-2-azetidinone | *H. foliosus* | aerial parts | (Urzúa 2004) |
| **Monoterpenes and monoterpenoids** | | | | |
| **Mon1** | *cis­α*­ocimene | *H. foliosus* | resin, aerial parts | (Urzúa et al. 2000; Urzúa 2004) |
| **Mon2** | *β*-ocimene | *H. foliosus* | stems | (Villagra et al. 2021) |
| **Mon3** | *β*-myrcene | *H. chrysanthemifolius* | flower heads | (Urzúa et al. 2007b) |
|  |  | *H. foliosus* | essential oil, stems | (Urzúa et al. 2010; Villagra et al. 2021) |
|  |  | *H. velutinus* | aerial parts | (Urzúa et al. 2004a) |
|  |  | *H. velutinus* subsp. *illinitus* | aerial parts | (Urzúa et al. 2004a) |
| **Mon4** | *α*-linalool | *H. bustillosianus* | aerial parts | (Urzúa et al. 2007a) |
| **Mon5** | linalyl anthranilate | *H. velutinus* | aerial parts | (Urzúa et al. 2004a) |
| **Mon6** | davanone | *H. velutinus* | aerial parts | (Urzúa et al. 2004a) |
| **Mon7** | davana ether | *H. velutinus* | aerial parts | (Urzúa et al. 2004a) |
| **Mon8** | limonene | *H. chrysanthemifolius* | flower heads | (Urzúa et al. 2007b) |
|  |  | *H. foliosus* | aerial parts, essential oil, stems | (Urzúa 2004; Urzúa et al. 2010; Villagra et al. 2021) |
|  |  | *H. velutinus* | aerial parts | (Urzúa et al. 2004a) |
|  |  | *H. velutinus* subsp. *illinitus* | aerial parts | (Urzúa et al. 2004a) |
| **Mon9** | *α*­terpinene | *H. foliosus* | resin, aerial parts, essential oil, stems | (Urzúa et al. 2000, 2010; Urzúa 2004; Villagra et al. 2021) |
| **Mon10** | *γ*­terpinene | *H. foliosus* | aerial parts, essential oil, stems | (Urzúa 2004; Urzúa et al. 2010; Villagra et al. 2021) |
| **Mon11** | terpinen-4-ol (*p*­menth­1­en­4­ol) | *H. foliosus* | aerial parts, essential oil, stems | (Urzúa 2004; Urzúa et al. 2010; Villagra et al. 2021) |
| **Mon12** | uroterpenol (9-hydroxy-α-terpineol) | *H. remyanus* | resin | (Faini et al. 2011) |
| **Mon13** | 9-benzoyloxy-(1-formyl)-*α*-terpineol | *H. remyanus* | resin | (Faini et al. 2011) |
| **Mon14** | 9-benzoyloxy-*α*-terpineol | *H. remyanus* | resin | (Faini et al. 2011) |
| **Mon15** | 7-hydroxy-9-benzoyloxy-*α*-terpineol | *H. remyanus* | resin | (Faini et al. 2011) |
| **Mon16** | terpinolene | *H. foliosus* | stems | (Villagra et al. 2021) |
| **Mon17** | isoterpinolene | *H. foliosus* | essential oil | (Urzúa et al. 2010) |
| **Mon18** | *α*-terpineol | *H. foliosus* | essential oil, stems | (Urzúa et al. 2010; Villagra et al. 2021) |
| **Mon19** | 1,2:8,9-diepoxy-*p*-menthane | *H. velutinus* | aerial parts | (Urzúa et al. 2004a) |
| **Mon20** | *p*­menth­2­en­4­ol | *H. foliosus* | resin, aerial parts | (Urzúa et al. 2000; Urzúa 2004) |
| **Mon21** | *trans*­*p*­menth­2­en­1­ol | *H. foliosus* | resin, aerial parts | (Urzúa et al. 2000; Urzúa 2004) |
| **Mon22** | *cis*­*p*­menth­2­en­1­ol | *H. foliosus* | resin, aerial parts | (Urzúa et al. 2000; Urzúa 2004) |
|  |  | *H. velutinus* | aerial parts | (Urzúa et al. 2004a) |
| **Mon23** | *trans*-pulegone oxide | *H. velutinus* | aerial parts | (Urzúa et al. 2004a) |
| **Mon24** | *α*-campholenal | *H. velutinus* | aerial parts | (Urzúa et al. 2004a) |
| **Mon25** | *α*-phellandrene | *H. foliosus* | essential oil, stems | (Urzúa et al. 2010; Villagra et al. 2021) |
| **Mon26** | 8-hydroxy-9-acetoxy-*β*-phellandrene | *H. remyanus* | aerial parts | (Zdero et al. 1991a) |
| **Mon27** | *m*­cymene | *H. foliosus* | resin | (Urzúa et al. 2000) |
|  |  | *H. velutinus* | aerial parts | (Urzúa et al. 2004a) |
| **Mon28** | *p*­cymene | *H. foliosus* | aerial parts, essential oil, stems | (Urzúa 2004; Urzúa et al. 2010; Villagra et al. 2021) |
| **Mon29** | *p*-cymen-8-ol | *H. foliosus* | stems | (Villagra et al. 2021) |
| **Mon30** | *o*-cumenol | *H. foliosus* | aerial parts, resin | (Urzúa et al. 2000; Urzúa 2004) |
| **Mon31** | 3­carene | *H. foliosus* | aerial parts, stems | (Urzúa 2004; Villagra et al. 2021) |
| **Mon32** | thujane | *H. foliosus* | resin, aerial parts | (Urzúa et al. 2000; Urzúa 2004) |
| **Mon33** | *α*-thujene | *H. foliosus* | essential oil, stems | (Urzúa et al. 2010; Villagra et al. 2021) |
|  |  | *H. velutinus* | aerial parts | (Urzúa et al. 2004a) |
| **Mon34** | *cis*-(+/-)-4-thujanol | *H. foliosus* | stems | (Villagra et al. 2021) |
| **Mon35** | 4-thujanol | *H. foliosus* | stems | (Villagra et al. 2021) |
| **Mon36** | *α*-thujone | *H. foliosus* | stems | (Villagra et al. 2021) |
| **Mon37** | *α*­pinene | *H. bustillosianus* | aerial parts | (Urzúa et al. 2007a) |
|  |  | *H. foliosus* | essential oil, stems | (Urzúa et al. 2010; Villagra et al. 2021) |
|  |  | *H. chrysanthemifolius* | flower heads | (Urzúa et al. 2007b) |
|  |  | *H. velutinus* | aerial parts | (Urzúa et al. 2004a) |
|  |  | *H. velutinus* subsp. *illinitus* | aerial parts | (Urzúa et al. 2004a) |
| **Mon38** | *β*­pinene | *H. bustillosianus* | aerial parts | (Urzúa et al. 2007a) |
|  |  | *H. chrysanthemifolius* | flower heads | (Urzúa et al. 2007b) |
|  |  | *H. foliosus* | resin, aerial parts, essential oil, stems | (Urzúa et al. 2000, 2010; Urzúa 2004; Villagra et al. 2021) |
|  |  | *H. velutinus* | aerial parts | (Urzúa et al. 2004a) |
|  |  | *H. velutinus* subsp. *illinitus* | aerial parts | (Urzúa et al. 2004a) |
| **Mon39** | pinocarveol | *H. foliosus* | aerial parts | (Urzúa 2004) |
|  |  | *H. velutinus* | aerial parts | (Urzúa et al. 2004a) |
| **Mon40** | *trans*-2-pinanol | *H. velutinus* | aerial parts | (Urzúa et al. 2004a) |
| **Mon41** | taedol | *H. taeda* | resin, stems | (Marambio and Silva 1989; Torres et al. 2005; Faini et al. 2008) |
| **Mon42** | borneol | *H. foliosus* | aerial parts, essential oil, stems | (Urzúa 2004; Urzúa et al. 2010; Villagra et al. 2021) |
| **Mon43** | bornyl acetate | *H. foliosus* | essential oil, stems | (Urzúa et al. 2010; Villagra et al. 2021) |
| **Mon44** | camphor | *H. foliosus* | stems | (Villagra et al. 2021) |
| **Mon45** | camphene | *H. foliosus* | essential oil, stems | (Urzúa et al. 2010; Villagra et al. 2021) |
| **Mon46** | fenchol | *H. foliosus* | aerial parts | (Urzúa 2004) |
| **Mon47** | *cis-*verbenol | *H. velutinus* | aerial parts | (Urzúa et al. 2004a) |
| **Mon48** | 1,5­dimethyl­6­methylenespiro[2.4]heptane | *H. foliosus* | aerial parts | (Urzúa 2004) |
| **Mon49** | sabinene | *H. foliosus* | essential oil, stems | (Urzúa et al. 2010; Villagra et al. 2021) |
| **Mon50** | 5­(acetyloxy)­4,6,6­trimethyl­endobiciclo[2.2.1]heptan­2­one | *H. foliosus* | aerial parts | (Urzúa 2004) |
| **Mon51** | 2,9-epoxy-*p*-menth-6-en-8-ol (haplopappol) | *H. multifolius* | aerial parts | (Maatooq et al. 2002) |
| **Mon52** | ascaridole | *H. foliosus* | stems | (Villagra et al. 2021) |
| **Mon53** | 3,3,7,7-tetramethyl-5-(2-methyl-1-propenyl)-tricyclo[4.1.0.0(2,4)]heptane | *H. uncinatus* | resin, aerial parts | (Urzúa et al. 2000, 2006) |
| **Mon54** | tricyclene | *H. foliosus* | resin, aerial parts | (Urzúa et al. 2000; Urzúa 2004) |
| **Sesquiterpenes and sesquiterpenoids** | | | | |
| **Sqt1** | *α-*farnesene | *H. scrobiculatus* | resin | (Urzúa et al. 2004b) |
| **Sqt2** | *β*-farnesene | *H. glutinosus* | aerial parts | (Jakupovic et al. 1986) |
| **Sqt3** | *α*-sinensal | *H. velutinus* | aerial parts | (Urzúa et al. 2004a) |
| **Sqt4** | *α*­bisabolol | *H. bustillosianus* | aerial parts | (Urzúa et al. 2007a) |
| **Sqt5** | humulene | *H. bustillosianus* | aerial parts | (Urzúa et al. 2007a) |
|  |  | *H. chrysanthemifolius* | flower heads | (Urzúa et al. 2007b) |
| **Sqt6** | humulene epoxide II | *H. velutinus* | aerial parts | (Urzúa et al. 2004a) |
| **Sqt7** | germacrene D | *H. deserticola* | aerial parts | (Zdero et al. 1990) |
|  |  | *H. foliosus* | essential oil, stems | (Urzúa et al. 2010; Villagra et al. 2021) |
|  |  | *H. glutinosus* | aerial parts | (Jakupovic et al. 1986) |
|  |  | *H. paucidentatus* | aerial parts | (Jakupovic et al. 1986) |
| **Sqt8** | bergamotol | *H. baylahuen* | essential oil (leaves) | (Becerra et al. 2010) |
| **Sqt9** | (1α,7β,10β)-11-hydroxy-4-guaien-3-one | *H. foliosus* | leaves | (Labbé et al. 1998) |
| **Sqt10** | (1β,7β,10β)-1,11-dihydroxy-4-guaien-3-one | *H. foliosus* | leaves | (Labbé et al. 1998) |
| **Sqt11** | (1α,6α,7β,10β)-6,11-dihydroxy-4-guaien-3-one | *H. foliosus* | leaves | (Labbé et al. 1998) |
| **Sqt12** | 9-*cis*-*p*-coumaroyloxy-*α*-terpineol (haplofolin) | *H. multifolius* | aerial parts | (Maatooq et al. 2002) |
| **Sqt13** | *α*-selinene | *H. foliosus* | aerial parts | (Urzúa 2004) |
| **Sqt14** | *γ*-selinene | *H. foliosus* | aerial parts | (Urzúa 2004) |
| **Sqt15** | 5­eudesmen­11­ol | *H. foliosus* | aerial parts | (Urzúa 2004) |
| **Sqt16** | *γ*-eudesmol | *H. foliosus* | aerial parts | (Urzúa 2004) |
| **Sqt17** | cadalene | *H. foliosus* | aerial parts | (Urzúa 2004) |
|  |  | *H. uncinatus* | resin, aerial parts | (Urzúa et al. 2000, 2006) |
| **Sqt18** | *α*­cadinene | *H. bustillosianus* | aerial parts | (Urzúa et al. 2007a) |
|  |  | *H. foliosus* | aerial parts | (Urzúa 2004) |
| **Sqt19** | *β*­cadinene | *H. foliosus* | aerial parts | (Urzúa 2004) |
|  |  | *H. schumannii* | aerial parts | (Urzúa et al. 2004a) |
|  |  | *H. velutinus* subsp. *illinitus* | aerial parts | (Urzúa et al. 2004a) |
| **Sqt20** | *γ*­cadinene | *H. bustillosianus* | aerial parts | (Urzúa et al. 2007a) |
|  |  | *H. foliosus* | aerial parts, essential oil | (Urzúa 2004; Urzúa et al. 2010) |
| **Sqt21** | *δ*­cadinene | *H. bustillosianus* | aerial parts | (Urzúa et al. 2007a) |
|  |  | *H. chrysanthemifolius* | flower heads | (Urzúa et al. 2007b) |
|  |  | *H. foliosus* | essential oil, stems | (Urzúa et al. 2010; Villagra et al. 2021) |
| **Sqt22** | guaiol | *H. foliosus* | aerial parts | (Urzúa 2004) |
| **Sqt23** | 1(10),11­eremophiladiene | *H. foliosus* | aerial parts | (Urzúa 2004) |
| **Sqt24** | *α*-cadinol | *H. baylahuen* | essential oil (leaves) | (Becerra et al. 2010) |
|  |  | *H. foliosus* | aerial parts | (Urzúa 2004) |
| **Sqt25** | ionene | *H. foliosus* | aerial parts | (Urzúa 2004) |
| **Sqt26** | 6-(1,1-dimethylethyl)-2,3-dihydro-1,1-dimethyl-3-methylene-1*H*-indene | *H. foliosus* | aerial parts | (Urzúa 2004) |
| **Sqt27** | *δ*­ambrinol | *H. foliosus* | resin, aerial parts | (Urzúa et al. 2000; Urzúa 2004) |
| **Sqt28** | decahydro-3a,8-dimethyl-5-(1-methylethenyl)azulene | *H. foliosus* | aerial parts | (Urzúa 2004) |
| **Sqt29** | 1,2,3,4,5,6,7,8-octahydro-1,4-dimethyl-7-(1-methylethylidene)azulene | *H. foliosus* | resin | (Urzúa et al. 2000) |
| **Sqt30** | (-)-isocaryophyllene | *H. bustillosianus* | aerial parts | (Urzúa et al. 2007a) |
|  |  | *H. chrysanthemifolius* | flower heads | (Urzúa et al. 2007b) |
| **Sqt31** | *β*-bulgarene | *H. chrysanthemifolius* | flower heads | (Urzúa et al. 2007b) |
| **Sqt32** | *γ*-bulgarene | *H. chrysanthemifolius* | flower heads | (Urzúa et al. 2007b) |
| **Sqt33** | (-)-amorpha-4,11-diene | *H. chrysanthemifolius* | flower heads | (Urzúa et al. 2007b) |
| **Sqt34** | caryophyllene oxide | *H. paucidentatus* | aerial parts | (Jakupovic et al. 1986) |
|  |  | *H. velutinus* | aerial parts | (Urzúa et al. 2004a) |
| **Sqt35** | *α*-guaiene | *H. velutinus* | aerial parts | (Urzúa et al. 2004a) |
| **Sqt36** | *β-*guaiene | *H. foliosus* | stems | (Villagra et al. 2021) |
| **Sqt37** | (-)-oplopanone | *H. velutinus* | aerial parts | (Urzúa et al. 2004a) |
| **Sqt38** | (-)-caryophyllene | *H. foliosus* | essential oil, stems | (Urzúa et al. 2010; Villagra et al. 2021) |
| **Sqt39** | *epi*-bicyclosesquiphellandrene | *H. foliosus* | essential oil | (Urzúa et al. 2010) |
| **Sqt40** | *α*-muurolene | *H. foliosus* | essential oil | (Urzúa et al. 2010) |
| **Sqt41** | *γ*-muurolene | *H. foliosus* | essential oil | (Urzúa et al. 2010) |
| **Sqt42** | agarospirol | *H. foliosus* | essential oil | (Urzúa et al. 2010) |
| **Sqt43** | 2,8-dimethyl-2´-vinyl-5-[4-methyl-pent-3-enyl]-chromane | *H. parvifolius* | aerial parts | (Zdero et al. 1991b) |
| **Sqt44** | 1*β*-hydroxy-*β*-cyperone | *H. arbutoides* | aerial parts | (Zdero et al. 1991a) |
| **Sqt45** | 8-oxo-*β*-cyperone | *H. paucidentatus* | aerial parts | (Jakupovic et al. 1986) |
| **Sqt46** | aphanamol I | *H. parvifolius* | aerial parts | (Zdero et al. 1991b) |
| **Sqt47** | aromadendrene | *H. foliosus* | aerial parts | (Urzúa 2004) |
|  |  | *H. uncinatus* | aerial parts | (Urzúa et al. 2004a) |
| **Sqt48** | *α*-cubebene | *H. bustillosianus* | aerial parts | (Urzúa et al. 2007a) |
|  |  | *H. chrysanthemifolius* | flower heads | (Urzúa et al. 2007b) |
|  |  | *H. foliosus* | essential oil | (Urzúa et al. 2010) |
|  |  | *H. uncinatus* | resin, aerial parts | (Urzúa et al. 2004a) |
| **Sqt49** | *β*-cubebene | *H. bustillosianus* | aerial parts | (Urzúa et al. 2007a) |
|  |  | *H. chrysanthemifolius* | flower heads | (Urzúa et al. 2007b) |
|  |  | *H. foliosus* | essential oil, stems | (Urzúa et al. 2010; Villagra et al. 2021) |
|  |  | *H. uncinatus* | resin, aerial parts | (Urzúa et al. 2004a) |
| **Sqt50** | (-)-calarene | *H. chrysanthemifolius* | flower heads | (Urzúa et al. 2007b) |
| **Sqt51** | 1,3,4,5,6,7-hexahydro-2,5,5-trimethyl-2*H*-2,4a-ethanonaphthalene | *H. chrysanthemifolius* | flower heads | (Urzúa et al. 2007b) |
| **Sqt52** | spathulenol | *H. foliosus* | essential oil | (Urzúa et al. 2010) |
|  |  | *H. velutinus* | aerial parts | (Urzúa et al. 2004a) |
|  |  | *H. uncinatus* | aerial parts | (Urzúa et al. 2004a) |
| **Sqt53** | cedryl acetate | *H. uncinatus* | aerial parts | (Urzúa et al. 2004a) |
| **Sqt54** | patchouli alcohol | *H. velutinus* | aerial parts | (Urzúa et al. 2004a) |
| **Sqt55** | *β*-bourbonene | *H. foliosus* | stems | (Villagra et al. 2021) |
|  |  | *H. schumannii* | aerial parts | (Urzúa et al. 2004a) |
|  |  | *H. uncinatus* | aerial parts | (Urzúa et al. 2004a) |
| **Sqt56** | globulol | *H. schumannii* | aerial parts | (Urzúa et al. 2004a) |
|  |  | *H. uncinatus* | aerial parts | (Urzúa et al. 2004a) |
| **Sqt57** | liguloxide | *H. rengifoanus* | aerial parts | (Zdero et al. 1991a) |
| **Sqt58** | *α*-copaene | *H. bustillosianus* | aerial parts | (Urzúa et al. 2007a) |
|  |  | *H. chrysanthemifolius* | flower heads | (Urzúa et al. 2007b) |
|  |  | *H. foliosus* | aerial parts, stems | (Urzúa 2004; Villagra et al. 2021) |
|  |  | *H. uncinatus* | resin, aerial parts | (Urzúa et al. 2000, 2006) |
| **Sqt59** | *β*-copaene | *H. foliosus* | stems | (Villagra et al. 2021) |
| **Sqt60** | *β*-ylangene | *H. foliosus* | stems | (Villagra et al. 2021) |
| **Diterpenes and diterpenoids** | | | | |
| **Dit1** | 6*α*-hydroxy-*ent*-labd-8(17)-en-15-oic acid | *H. chrysanthemifolius* | leaves | (Faini et al. 1999) |
| **Dit2** | 3*β*-acetoxy-*ent*-labd-8(17)-en-15-oic acid | *H. chrysanthemifolius* | leaves | (Faini et al. 1999) |
| **Dit3** | 18*α*-acetoxylabd-8(17)-en-15-oic acid | *H. chrysanthemifolius* | leaves | (Faini et al. 1999) |
|  |  | *H. litoralis* | resin | (Urzúa et al. 2004b) |
| **Dit4** | 7*α*-hydroxylabd-8(17)-en-15,18-dioic acid | *H. pulchellus* | aerial parts | (Zdero et al. 1991a) |
| **Dit5** | 7*α*-hydroxylabd-8(17)-en-15,18-dioic acid-15-methylester | *H. velutinus* subsp. *illinitus* | aerial parts | (Faini et al. 2002) |
| **Dit6** | rigiduside (13-O-*β*-xylopyranosylmanool) | *H. rigidus* | aerial parts | (Morales et al. 2000a, 2003) |
| **Dit7** | manool | *H. schumannii* | aerial parts | (Urzúa et al. 2004a) |
| **Dit8** | 13-*O*-*β-*xylopyranosyl-*ent*-manool | *H. diplopappus* Remy | resin | (Urzúa et al. 1995a) |
| **Dit9** | *ent*-manool | *H. diplopappus* Remy | resin | (Urzúa et al. 1995a) |
| **Dit10** | 7-oxo-labd-8(9)-en-15,18-dioic acid-15-methylester | *H. velutinus* subsp. *illinitus* | aerial parts | (Faini et al. 2002) |
| **Dit11** | dehydropinipholic acid 19-methyl ester | *H. velutinus* | aerial parts | (Urzúa and Mendoza 1989, 1993) |
| **Dit12** | 4*α*-hydroxy-18-norlabd-8(17)-en-15-oic acid | *H. velutinus* | aerial parts | (Urzúa and Mendoza 1993; Urzúa et al. 1995b) |
| **Dit13** | 4*β*-hydroxy-19-norlabd-8(17)-en-15-oic acid | *H. velutinus* | aerial parts | (Urzúa and Mendoza 1993; Urzúa et al. 1995b) |
| **Dit14** | 18-hydroxylabd-8(17)-en-15-oic acid | *H. litoralis* | resin | (Urzúa et al. 2004b) |
|  |  | *H. velutinus* | aerial parts | (Urzúa and Mendoza 1993; Urzúa et al. 1995b) |
| **Dit15** | 18-hydroxymanool (torulosol) | *H. scrobiculatus* | resin | (Urzúa et al. 2004b) |
| **Dit16** | (+)-copalic acid | *H. litoralis* | resin | (Urzúa et al. 2004b) |
| **Dit17** | methyl-*ent*-4-*epi*-agath-18-oate | *H. deserticola* | aerial parts | (Zdero et al. 1990) |
| **Dit18** | dimethyl-*ent*-4-*epi*-agathoate | *H. deserticola* | aerial parts | (Zdero et al. 1990) |
| **Dit19** | copaiferolic acid | *H. deserticola* | aerial parts | (Zdero et al. 1990) |
| **Dit20** | copaiferolic acid 15-methyl ester | *H. deserticola* | aerial parts | (Zdero et al. 1990) |
| **Dit21** | (-)-eperuic acid | *H. litoralis* | resin | (Urzúa et al. 2004b) |
|  |  | *H. schumannii* | aerial parts | (Urzúa et al. 2004a) |
| **Dit22** | pinifolic acid 15-methyl ester | *H. velutinus* subsp. *illinitus* | aerial parts | (Urzúa et al. 2004a) |
| **Dit23** | pinifolic acid 18-methyl ester | *H. velutinus* subsp. *illinitus* | aerial parts | (Urzúa et al. 2004a) |
| **Dit24** | pinifolic acid dimethyl ester | *H. velutinus* subsp. *illinitus* | aerial parts | (Urzúa et al. 2004a) |
| **Dit25** | *epi*-manool | *H. schumannii* | aerial parts | (Urzúa et al. 2004a) |
| **Dit26** | methyl haplodesertoate | *H. deserticola* | aerial parts | (Zdero et al. 1990) |
| **Dit27** | 13-hydroxylabda-6,8,14-triene | *H. parvifolius* | aerial parts | (Zdero et al. 1991b) |
| **Dit28** | 13-hydroxylabda-6,8(17),14-triene | *H. parvifolius* | aerial parts | (Zdero et al. 1991b) |
| **Dit29** | 9*α*,13-epoxy-labda-6,8(17),14-triene | *H. parvifolius* | aerial parts | (Zdero et al. 1991b) |
| **Dit30** | 6*β*-acetoxy-13-hydroxylabda-8,14-dien-7-one | *H. parvifolius* | aerial parts | (Zdero et al. 1991b) |
| **Dit31** | 6*β*-acetoxy-7*β*,13-dihydroxylabda-8,14-diene | *H. parvifolius* | aerial parts | (Zdero et al. 1991b) |
| **Dit32** | 15-oxo-labda-8(17),14*E*-diene-18-oic acid | *H. arbutoides* | aerial parts | (Zdero et al. 1991a) |
| **Dit33** | 15-oxo-labda-8(17),14*Z*-diene-18-oic acid | *H. arbutoides* | aerial parts | (Zdero et al. 1991a) |
| **Dit34** | labda-8(17),13*E*-dien-15,18-dioic acid 15-methyl ester | *H. arbutoides* | aerial parts | (Zdero et al. 1991a; Rossomando et al. 1995) |
| **Dit35** | 15-hydroxylabd-8(17)-en-18-oic acid | *H. arbutoides* | aerial parts | (Zdero et al. 1991a) |
| **Dit36** | labd-7-en-15,18-dioic acid | *H. pulchellus* | aerial parts | (Zdero et al. 1991a) |
|  |  | *H. velutinus* subsp. *illinitus* | aerial parts | (Faini et al. 2002) |
| **Dit37** | labd-7-en-15,18-dioic acid-18*α*-methylester | *H. velutinus* | resin | (Echeverría et al. 2019) |
|  |  | *H. velutinus* subsp. *illinitus* | aerial parts | (Faini et al. 2002) |
| **Dit38** | labd-7-en-15,18-dioic acid-15-methylester | *H. velutinus* subsp. *illinitus* | aerial parts | (Faini et al. 2002) |
| **Dit39** | 18-hydroxylabda-7,13(*E*)-dien-15-oic acid | *H. multifolius* | aerial parts | (Maatooq et al. 2002) |
|  |  | *H. remyanus* | resin, aerial parts | (Zdero et al. 1991a; Faini et al. 2011) |
|  |  | *H. taeda* | stems | (Marambio and Silva 1989) |
| **Dit40** | 18-acetoxy-labda-7,13(*E*)-dien-15-oic acid | *H. remyanus* | resin | (Faini et al. 2011) |
| **Dit41** | 6,18-dihydroxy-*ent*-labd-7,13*E*-dien-15-oic acid | *H. glutinosus* | aerial parts | (Jakupovic et al. 1986) |
| **Dit42** | 18-hydroxylabda-7,13(*Z*)-dien-15-oic acid | *H. multifolius* | aerial parts | (Maatooq et al. 2002) |
| **Dit43** | 7,13-labdadien-15,18-dioic acid | *H. taeda* | stems | (Marambio and Silva 1989) |
| **Dit44** | 7,13-labdadien-15,18-dioic acid 15-methyl ester | *H. coquimbensis* | aerial parts | (Maldonado et al. 1993) |
| **Dit45** | 7,13-(*E*)-labdadien-15,18-dioic acid 18-methyl ester | *H. velutinus* | resin | (Echeverría et al. 2019) |
| **Dit46** | 18-dihydrocinnamoyloxy-labda-7,13*E*-dien-l5-oic acid | *H. remyanus* | aerial parts | (Zdero et al. 1991a) |
| **Dit47** | 6*β*-acetoxy-13-hydroxylabda-7,14-diene | *H. parvifolius* | aerial parts | (Zdero et al. 1991b) |
| **Dit48** | 13-hydroxy-6*α*-butyryloxylabda-7,14-diene | *H. parvifolius* | aerial parts | (Zdero et al. 1991b) |
| **Dit49** | 13-hydroxylabda-7,14-diene-6-one | *H. parvifolius* | aerial parts | (Zdero et al. 1991b) |
| **Dit50** | 9*α*,13-dihydroxylabda-7,14-dien-6-one | *H. parvifolius* | aerial parts | (Zdero et al. 1991b) |
| **Dit51** | 6*α*,13-dihydroxylabda-7,14-dien-17-al | *H. parvifolius* | aerial parts | (Zdero et al. 1991b) |
| **Dit52** | isomanool | *H. parvifolius* | aerial parts | (Zdero et al. 1991b) |
| **Dit53** | 6*α*-hydroxy-9*α*,13-epoxy-labda-7,14-diene | *H. parvifolius* | aerial parts | (Zdero et al. 1991b) |
| **Dit54** | 6*α*-acetoxy-9*α*,13-epoxy-labda-7,14-diene | *H. parvifolius* | aerial parts | (Zdero et al. 1991b) |
| **Dit55** | 6*α*-butyryloxy-9*α*,13-epoxy-labda-7,14-diene | *H. parvifolius* | aerial parts | (Zdero et al. 1991b) |
| **Dit56** | 5*α*-hydroxy-9*α*,13-epoxy-labda-7,14-diene-6-one | *H. parvifolius* | aerial parts | (Zdero et al. 1991b) |
| **Dit57** | 6*α*-acetoxy-9α,13-epoxy-labda-7,14-dien-17-al | *H. parvifolius* | aerial parts | (Zdero et al. 1991b) |
| **Dit58** | 6-oxo-14,15-*nor*-labda-7-ene | *H. parvifolius* | aerial parts | (Zdero et al. 1991b) |
| **Dit59** | 8*α*-hydroxylabdan-15-oic acid (labdanolic acid) | *H. schumannii* | resin | (Urzúa et al. 1997) |
| **Dit60** | 8*α*-hydroxyanticopalic acid | *H. deserticola* | aerial parts | (Zdero et al. 1990) |
| **Dit61** | 8*α*-hydroxyanticopalic acid methyl ester | *H. deserticola* | aerial parts | (Zdero et al. 1990) |
| **Dit62** | labd-13(E)-ene-8α,15-diol | *H. arbutoides* | aerial parts | (Zdero et al. 1991a) |
|  |  | *H. bezanillanus* | aerial parts | (Maldonado et al. 1993) |
| **Dit63** | 13*R-*labdane-8,15-diol | *H. arbutoides* | aerial parts | (Zdero et al. 1991a) |
| **Dit64** | 8*α*-hydroxy-*ent*-labd-13(14)*Z*-en-15-al | *H. arbutoides* | aerial parts | (Zdero et al. 1991a) |
| **Dit65** | 8*α*-hydroxylabdan-15-al | *H. arbutoides* | aerial parts | (Zdero et al. 1991a) |
| **Dit66** | 8*α*,13-dihydroxylabda-6,14-diene | *H. parvifolius* | aerial parts | (Zdero et al. 1991b) |
| **Dit67** | 8*α*,13-dihydroxylabda-5,14-dien-7-one | *H. parvifolius* | aerial parts | (Zdero et al. 1991b) |
| **Dit68** | *epi*-manoyl oxide | *H. arbutoides* | aerial parts | (Zdero et al. 1991a) |
|  |  | *H. parvifolius* | aerial parts | (Zdero et al. 1991b) |
| **Dit69** | 6,7-dehydro-13-*epi*-manoyl oxide | *H. parvifolius* | aerial parts | (Zdero et al. 1991b) |
| **Dit70** | 6,7-dehydro-8,13-bis-*epi*-manoyl oxide | *H. parvifolius* | aerial parts | (Zdero et al. 1991b) |
| **Dit71** | 13,17-epoxy-labda-5,7,14-triene | *H. parvifolius* | aerial parts | (Zdero et al. 1991b) |
| **Dit72** | 9*α*,13-epoxy-5*α*,8*α*-dihydroxylabda-6,14-diene | *H. parvifolius* | aerial parts | (Zdero et al. 1991b) |
| **Dit73** | 5*α*-hydroxy-7,8-epoxy-7,8-*seco*-6,7-dehydro-13-*epi*-manoyl oxide | *H. parvifolius* | aerial parts | (Zdero et al. 1991b) |
| **Dit74** | 8,13-epoxy-14-labdeb-3-ol | *H. arbutoides* | aerial parts | (Zdero et al. 1991a) |
| **Dit75** | 8,13-epoxy-labdan-15-al | *H. arbutoides* | aerial parts | (Zdero et al. 1991a) |
| **Dit76** | 18-acetoxy-friedolabd-5-en-15-oic acid | *H. pulchellus* | aerial parts | (Zdero et al. 1991a) |
| **Dit77** | 18-acetoxy-friedolabd-5-en-7-one-15-oic acid | *H. pulchellus* | aerial parts | (Zdero et al. 1991a) |
| **Dit78** | 18-hydroxy-friedolabd-5-en-15-oic acid | *H. paucidentatus* | aerial parts | (Jakupovic et al. 1986) |
|  |  | *H. pulchellus* | aerial parts | (Zdero et al. 1991a) |
| **Dit79** | 18-hydroxy-7-oxo-friedolabd-5-en-15-oic acid | *H. pulchellus* | aerial parts | (Zdero et al. 1991a) |
| **Dit80** | friedolabd-5-en-15,18-dioic acid | *H. pulchellus* | aerial parts | (Zdero et al. 1991a) |
| **Dit81** | 15-hydroxy-friedolabd-5-en-18-oic acid | *H. pulchellus* | aerial parts | (Zdero et al. 1991a) |
| **Dit82** | 18-acetoxy-*cis*-clerode 3,13(*Z*)-dien-15 oic acid | *H. rigidus* | aerial parts | (Morales et al. 2003) |
| **Dit83** | 18-hydroxy-*cis*-cleroda-3-en-15-oic acid (10*βH*, 16*ξ,* 19*β*, 17*β*, 20*α* form) | *H. paucidentatus* | aerial parts | (Jakupovic et al. 1986) |
| **Dit84** | 18-acetoxy-*cis*-cleroda-3-en-15-oic acid (10*βH*, 16*ξ,* 19*β*, 17*β*, 20*α* form) | *H. uncinatus* | aerial parts | (Urzúa et al. 2006) |
| **Dit85** | 19-hydroxy-*cis*-cleroda-3-en-15-oic acid (10*βH*, 16*ξ,* 19*β*, 17*β*, 20*α* form) | *H. paucidentatus* | aerial parts | (Jakupovic et al. 1986) |
| **Dit86** | 2-oxoclerod-3-en-15-oic acid (2-oxopopulifolic acid) | *H. schumannii* | resin | (Urzúa et al. 1997) |
| **Dit87** | 2*α*-hydroxy-*cis*-clero-3,13(*Z*),8(17)-trien-15-oic acid | *H. foliosus* | aerial parts | (Urzúa et al. 2003) |
| **Dit88** | 2*α*-acetoxy-*cis*-clero-3,13(*Z*),8(17)-trien-15-oic acid | *H. foliosus* | aerial parts | (Urzúa et al. 2003) |
| **Dit89** | populifolic acid | *H. bustillosianus* | aerial parts | (Urzúa et al. 2007a) |
| **Dit90** | populifolic acid methyl ester | *H. bustillosianus* | aerial parts | (Urzúa et al. 2007a) |
| **Dit91** | 15-oxocleroda-3,13*E*-dien-18-oic acid | *H. arbutoides* | aerial parts | (Zdero et al. 1991a) |
| **Dit92** | 15-oxocleroda-3,13*Z*-dien-18-oic acid | *H. arbutoides* | aerial parts | (Zdero et al. 1991a) |
| **Dit93** | 18-hydroxy-*cis*-cleroda-3,13(*E*)-dien-15-oic acid | *H. paucidentatus* | aerial parts | (Jakupovic et al. 1986) |
| **Dit94** | 2-oxokolavenic acid | *H. scrobiculatus* | aerial parts | (Rossomando et al. 1995) |
| **Dit95** | cleroda-3,13 (*E*)-dien-15,18-diol | *H. taeda* | resin | (Faini et al. 2007) |
| **Dit96** | haplopappic acid | *H. angustifolius* | aerial parts | (Silva and Sammes 1973) |
|  |  | *H. foliosus* | aerial parts | (Silva and Sammes 1973) |
| **Dit97** | haplopappic methylester | *H. angustifolius* | aerial parts | (Silva and Sammes 1973) |
| **Dit98** | *ent*-19-hydroxy-*cis*-cleroda-3,13(*E*)-dien-15-oic acid | *H. deserticola* | resin | (Tojo et al. 1999) |
| **Dit99** | 18-acetoxy-*cis*-cleroda-3,13(*E*)-dien-15-oic acid | *H. deserticola* | resin | (Urzúa Moll et al. 1997; Tojo et al. 1999) |
|  |  | *H. paucidentatus* | aerial parts | (Jakupovic et al. 1986) |
|  |  | *H. taeda* | - | (Faini et al. 2008) |
| **Dit100** | rigidusol [13-hydroxy-18-acetoxy-*cis*-cleroda-3,14-diene (8*β*H,10*β*H, 19*β*, 20*α* form)] | *H. rigidus* | aerial parts | (Morales et al. 2000b, 2003) |
| **Dit101** | deacetylrigidusol [13,18-dihydroxy-*cis*-cleroda-3,14-diene (8*β*H,10*β*H, 19*β*, 20*α* form)] | *H. rigidus* | aerial parts | (Morales et al. 2000b, 2003) |
| **Dit102** | thunbergol | *H. bustillosianus* | aerial parts | (Urzúa et al. 2007a) |
| **Dit103** | haploparvone | *H. parvifolius* | aerial parts | (Zdero et al. 1991b) |
| **Dit104** | 5*α*-hydroxyhaploparvone | *H. parvifolius* | aerial parts | (Zdero et al. 1991b) |
| **Dit105** | haploparviolide | *H. parvifolius* | aerial parts | (Zdero et al. 1991b) |
| **Dit106** | 1,1,5,6-tetramethyl-4-[3-hydroxy-3-methyl-pent-(4)-enyl]-tetralin | *H. parvifolius* | aerial parts | (Zdero et al. 1991b) |
| **Dit107** | 1,1,5-trimethyl-6-(3-hydroxy-3-methyl-pent-4-enyl)-tetralin | *H. parvifolius* | aerial parts | (Zdero et al. 1991b) |
| **Triterpenes and triterpenoids** | | | | |
| **Tri1** | friedelin (friedelan-3-one) | *H. angustifolius* | aerial parts | (Silva and Sammes 1973) |
|  |  | *H. foliosus* | aerial parts | (Silva and Sammes 1973) |
|  |  | *H. velutinus* | stems | (Marambio and Silva 1996) |
| **Tri2** | *epi*-friedelin | *H. coquimbensis* | aerial parts | (Maldonado et al. 1993) |
| **Tri3** | *epi*-friedelinol | *H. angustifolius* | aerial parts | (Silva and Sammes 1973) |
|  |  | *H. foliosus* | aerial parts | (Silva and Sammes 1973) |
|  |  | *H. velutinus* | stems | (Marambio and Silva 1996) |
| **Tri4** | taraxerol | *H. velutinus* | stems | (Marambio and Silva 1996) |
| **Tri5** | erythrodiol | *H. velutinus* | stems | (Marambio and Silva 1996) |
| **Meroterpenoids** | | | | |
| **Mer1** | procerin | *H. velutinus* subsp. *illinitus* | aerial parts | (Urzúa et al. 2004a) |
| **Steroids** | | | | |
| **Str1** | stigmasterol | *H. coquimbensis* | aerial parts | (Maldonado et al. 1993) |
|  |  | *H. foliosus* | aerial parts | (Silva and Sammes 1973) |
|  |  | *H. velutinus* | stems | (Marambio and Silva 1996) |
| **Str2** | *β*-sitosterol | *H. bezanillanus* | aerial parts | (Maldonado et al. 1993) |
|  |  | *H. velutinus* | stems | (Marambio and Silva 1996) |
|  |  | *H. velutinus* subsp. *illinitus* | leaves | (Latorre et al. 1990) |
| **Flavonoids** | | | | |
| *** Flavonols** | | | | |
| **Flv1** | quercetin | *H. baylahuen* J. Remy | aerial parts | (Hörhammer et al. 1973) |
|  |  | *H. chrysanthemifolius* | flower heads | (Urzúa et al. 2007b) |
|  |  | *H. deserticola* | aerial parts | (Schmeda-Hirschmann et al. 2015) |
|  |  | *H. multifolius* | aerial parts, resin | (Nuñez-Alarcón and Quiñones 1995; Urzúa et al. 1995b; Torres et al. 2006; Schmeda-Hirschmann et al. 2015) |
|  |  | *H. integerrimus* | leaves | (Ayanoglu et al. 1981) |
|  |  | *H. remyanus* | resin | (Faini et al. 2011) |
|  |  | *H. schumannii* | leaves | (Ates et al. 1982) |
|  |  | *H. scrobiculatus* | leaves | (Ates et al. 1982) |
|  |  | *H. taeda* | aerial parts | (Schmeda-Hirschmann et al. 2015) |
|  |  | *H. velutinus* | stems | (Marambio and Silva 1996) |
|  |  | *H. velutinus* subsp. *illinitus* | leaves | (Latorre et al. 1990) |
| **Flv2** | quercetin 3-methyl ether (3-methoxyluteolin) | *H. baylahuen* | aerial parts | (Hörhammer et al. 1973; Schmeda-Hirschmann et al. 2015) |
|  |  | *H. deserticola* | aerial parts | (Schmeda-Hirschmann et al. 2015) |
|  |  | *H. foliosus* | leaves | (Ulubelen et al. 1982) |
|  |  | *H. integerrimus* | leaves | (Ayanoglu et al. 1981) |
|  |  | *H. multifolius* | aerial parts | (Nuñez-Alarcón and Quiñones 1995; Schmeda-Hirschmann et al. 2015) |
|  |  | *H. rigidus* | aerial parts | (Schmeda-Hirschmann et al. 2015) |
|  |  | *H. taeda* | aerial parts | (Schmeda-Hirschmann et al. 2015) |
| **Flv3** | tamarixetin (quercetin 4´-methyl ether) | *H. chrysanthemifolius* | flower heads | (Urzúa et al. 2007b) |
| **Flv4** | rhamnazin (quercetin 7,3´-dimethyl ether) | *H. foliosus* | aerial parts | (Urzúa 2004) |
|  |  | *H. integerrimus* | leaves | (Ayanoglu et al. 1981) |
| **Flv5** | quercetin 3,3´-dimethyl ether | *H. integerrimus* | leaves | (Ayanoglu et al. 1981) |
| **Flv6** | quercetin 3,7-dimethyl ether | *H. integerrimus* | leaves | (Ayanoglu et al. 1981) |
|  |  | *H. taeda* | stems | (Marambio and Silva 1989) |
| **Flv7** | ayanin | *H. chrysanthemifolius* | leaves, resin | (Faini et al. 1999; Urzúa et al. 2012) |
|  |  | *H. litoralis* | resin | (Urzúa et al. 2012) |
| **Flv8** | retusin (5-hydroxy-3,7,3´,4´-tetramethoxyflavone) | *H. litoralis* | resin | (Urzúa et al. 2012) |
| **Flv9** | 3-*O*-acetyl-7-methylquercetin | *H. remyanus* | resin | (Faini et al. 2011) |
| **Flv10** | isoquercitrin (quercetin-3-*β*-D-glucoside) | *H. foliosus* | leaves | (Ulubelen et al. 1982) |
|  |  | *H. integerrimus* | leaves | (Ayanoglu et al. 1981) |
|  |  | *H. schumannii* | leaves | (Ates et al. 1982) |
|  |  | *H. scrobiculatus* | leaves | (Ates et al. 1982) |
|  |  | *H. velutinus* | stems | (Marambio and Silva 1996) |
| **Flv11** | hyperoside (quercetin-3-*β*-D-galactoside) | *H. foliosus* | leaves | (Ulubelen et al. 1982) |
| **Flv12** | quercetagetin 3-methyl ether | *H. rengifoanus* | leaves | (Ulubelen et al. 1981) |
| **Flv13** | quercetagetin 3,7-dimethyl ether | *H. rengifoanus* | leaves | (Ulubelen et al. 1981) |
| **Flv14** | centaureidin | *H. poeppigianus* | aerial parts | (Oksuz et al. 1981) |
| **Flv15** | beturetol (3,5,7-trihydroxy-6,4´-dimethoxyflavone) | *H. foliosus* | roots | (Tschesche et al. 1985) |
|  |  | *H. rigidus* | aerial parts | (Morales et al. 2000a, 2003) |
| **Flv16** | eupatolitin | *H. foliosus* | leaves, aerial parts | (Ulubelen et al. 1982; Urzúa 2004) |
| **Flv17** | rhamnetin | *H. baylahuen* | leaves, resin | (Nuñez-Alarcon et al. 1993; Vera et al. 2001) |
| **Flv18** | isorhamnetin | *H. baylahuen* | leaves, resin | (Nuñez-Alarcon et al. 1993; Vera et al. 2001) |
|  |  | *H. foliosus* | leaves, aerial parts | (Ulubelen et al. 1982; Urzúa 2004) |
|  |  | *H. integerrimus* | leaves | (Ayanoglu et al. 1981) |
|  |  | *H. multifolius* | aerial parts, resin | (Urzúa et al. 1995b; Torres et al. 2006) |
|  |  | *H. rengifoanus* | leaves | (Ulubelen et al. 1981) |
|  |  | *H. scrobiculatus* | leaves | (Ates et al. 1982) |
| **Flv19** | isorhamnetin 3-*β*-D-glucoside | *H. foliosus* | leaves | (Ulubelen et al. 1982) |
|  |  | *H. rengifoanus* | leaves | (Ulubelen et al. 1981) |
|  |  | *H. scrobiculatus* | leaves | (Ates et al. 1982) |
| **Flv20** | isorhamnetin 3-*β*-D-galactoside | *H. rengifoanus* | leaves | (Ulubelen et al. 1981) |
| **Flv21** | kaempferol | *H. baylahuen* | aerial parts, resin | (Hörhammer et al. 1973; Nuñez-Alarcon et al. 1993) |
|  |  | *H. foliosus* | leaves, aerial parts | (Ulubelen et al. 1982; Urzúa 2004) |
|  |  | *H. rigidus* | aerial parts | (Schmeda-Hirschmann et al. 2015) |
|  |  | *H. taeda* | aerial parts | (Schmeda-Hirschmann et al. 2015) |
| **Flv22** | astragalin (kaempferol 3-*β*-D-glucoside) | *H. foliosus* | leaves | (Ulubelen et al. 1982) |
| **Flv23** | isokaempferide (kaempferol 3-methyl ether) | *H. deserticola* | resin | (Tojo et al. 1999) |
|  |  | *H. foliosus* | leaves, aerial parts, roots | (Ulubelen et al. 1982; Tschesche et al. 1985; Urzúa 2004) |
|  |  | *H. glutinosus* | aerial parts | (Valant-Vetschera and Wollenweber 2007) |
|  |  | *H. rigidus* | aerial parts | (Schmeda-Hirschmann et al. 2015) |
|  |  | *H. velutinus* | resin | (Urzúa et al. 1991; Urzúa and Mendoza 1993) |
| **Flv24** | kaempferol 3-methyl ether 7-*β*-D-glucoside | *H. foliosus* | leaves | (Ulubelen et al. 1982) |
| **Flv25** | rhamnocitrin (kaempferol 7-methyl ether) | *H. baylahuen* | resin | (Nuñez-Alarcon et al. 1993) |
|  |  | *H. scrobiculatus* | leaves | (Ates et al. 1982) |
| **Flv26** | ermanin (kaempferol 3,4'-dimethyl ether) | *H. foliosus* | aerial parts, roots | (Tschesche et al. 1985; Urzúa 2004) |
|  |  | *H. glutinosus* | aerial parts | (Valant-Vetschera and Wollenweber 2007) |
| **Flv27** | kaempferol 7,4´-dimethyl ether | *H. remyanus* | resin | (Faini et al. 2011) |
|  |  | *H. coquimbensis* | aerial parts | (Maldonado et al. 1993) |
| **Flv28** | kumatakenin (kaempferol 3,7-dimethyl ether) | *H. foliosus* | aerial parts | (Urzúa 2004) |
|  |  | *H. velutinus* | resin | (Urzúa et al. 1991; Urzúa and Mendoza 1993) |
| **Flv29** | kaempferol 3,7,4´-trimethyl ether | *H. coquimbensis* | aerial parts | (Maldonado et al. 1993) |
|  |  | *H. remyanus* | resin | (Faini et al. 2011) |
| **Flv30** | 3-*O*-acetyl-7,4´-dimethylkaempferol | *H. remyanus* | resin | (Faini et al. 2011) |
| **Flv31** | haplopappin | *H. foliosus* | roots | (Tschesche et al. 1985) |
| **Flv32** | haplopappin A | *H. foliosus* | roots | (Tschesche et al. 1985) |
| **Flv33** | myricetin | *H. poeppigianus* | aerial parts | (Oksuz et al. 1981) |
| **Flv34** | myricetin 3´,4´dimethyl ether | *H. integerrimus* | leaves | (Ayanoglu et al. 1981) |
| **Flv35** | myricetin 3,3´,4´-trimethyl ether | *H. integerrimus* | leaves | (Ayanoglu et al. 1981) |
| **Flv36** | myricetin 3,7,4´-trimethyl ether | *H. chrysanthemifolius* | leaves, resin | (Faini et al. 1999; Urzúa et al. 2012) |
| **Flv37** | 3,8-dimethylherbacetin (5,7,4'-trihydroxy-3,8-dimethoxyflavone) | *H. deserticola* | resin | (Tojo et al. 1999) |
| **Flv38** | 3,8,4´-trimethylherbacetin (5,7-dihydroxy-3,8,4'-trimethoxyflavone) | *H. deserticola* | resin | (Tojo et al. 1999) |
| **Flv39** | 5,7,4'-trihydroxy-3,8,3´-trimethoxyflavone | *H. deserticola* | resin | (Tojo et al. 1999) |
| **Flv40** | 3,5-dihydroxy-3´,4´,6,7-tetramethoxyflavone | *H. uncinatus* | aerial parts | (Urzúa et al. 2006) |
| **Flv41** | santin | *H. arbutoides* | aerial parts | (Rossomando et al. 1995) |
|  |  | *H. bustillosianus* | aerial parts | (Urzúa et al. 2007a) |
|  |  | *H. glutinosus* | aerial parts | (Valant-Vetschera and Wollenweber 2007) |
|  |  | *H. scrobiculatus* | resin, aerial parts | (Rossomando et al. 1995; Urzúa et al. 2012) |
| **Flv42** | eupatorin | *H. scrobiculatus* | resin | (Urzúa et al. 2012) |
| **Flv43** | jaceidin | *H. glutinosus* | aerial parts | (Valant-Vetschera and Wollenweber 2007) |
| **Flv44** | jaceidin 7-methyl ether | *H. bezanillanus* | aerial parts | (Maldonado et al. 1993) |
| **Flv45** | penduletin | *H. arbutoides* | aerial parts | (Rossomando et al. 1995) |
|  |  | *H. scrobiculatus* | aerial parts | (Rossomando et al. 1995) |
| **Flv46** | pachypodol | *H. coquimbensis* | aerial parts | (Maldonado et al. 1993) |
| *** Flavones** | | | | |
| **Flv47** | apigenin | *H. glutinosus* | aerial parts | (Valant-Vetschera and Wollenweber 2007) |
|  |  | *H. rengifoanus* | leaves | (Ulubelen et al. 1981) |
| **Flv48** | 3,6-dimethoxyapigenin | *H. bustillosianus* | aerial parts | (Urzúa et al. 2007a) |
|  |  | *H. glutinosus* | aerial parts | (Valant-Vetschera and Wollenweber 2007) |
| **Flv49** | vicenin-2 | *H. schumannii* | leaves | (Ates et al. 1982) |
|  |  | *H. scrobiculatus* | leaves | (Ates et al. 1982) |
| **Flv50** | vitexin | *H. schumannii* | leaves | (Ates et al. 1982) |
|  |  | *H. scrobiculatus* | leaves | (Ates et al. 1982) |
| **Flv51** | isovitexin | *H. schumannii* | leaves | (Ates et al. 1982) |
|  |  | *H. scrobiculatus* | leaves | (Ates et al. 1982) |
| **Flv52** | isoschaftoside | *H. scrobiculatus* | leaves | (Ates et al. 1982) |
| **Flv53** | luteolin | *H. chrysanthemifolius* | flower heads | (Urzúa et al. 2007b) |
|  |  | *H. rengifoanus* | leaves | (Ulubelen et al. 1981) |
|  |  | *H. velutinus* | stems | (Marambio and Silva 1996) |
| **Flv54** | luteolin 5-glucoside | *H. glutinosus* | leaves | (Marambio and Silva 1996) |
| **Flv55** | luteolin 7-glucoside | *H. glutinosus* | leaves | (Marambio and Silva 1996) |
| **Flv56** | chrysoeriol | *H. poeppigianus* | aerial parts | (Oksuz et al. 1981) |
| **Flv57** | velutin (luteolin 7, 3´-dimethyl ether) | *H. baylahuen* | resin | (Nuñez-Alarcon et al. 1993) |
| **Flv58** | diosmetin | *H. chrysanthemifolius* | flower heads | (Urzúa et al. 2007b) |
|  |  | *H. poeppigianus* | aerial parts | (Oksuz et al. 1981) |
| **Flv59** | eupafolin (6-methoxyluteolin) | *H. scrobiculatus* | leaves | (Ates et al. 1982) |
| **Flv60** | 6-methoxyluteolin 4´-methyl ether | *H. scrobiculatus* | leaves | (Ates et al. 1982) |
| **Flv61** | cirsiliol (6-methoxyluteolin 7-methyl ether) | *H. scrobiculatus* | leaves | (Ates et al. 1982) |
| **Flv62** | hispidulin (scutellarein 6-methyl ether) | *H. glutinosus* | aerial parts | (Valant-Vetschera and Wollenweber 2007) |
|  |  | *H. poeppigianus* | aerial parts | (Oksuz et al. 1981) |
| **Flv63** | pectolinaringenin | *H. glutinosus* | aerial parts | (Valant-Vetschera and Wollenweber 2007) |
| **Flv64** | scutellarein 6-*β*-D-glucoside | *H. rengifoanus* | leaves | (Ulubelen et al. 1981) |
|  |  | *H. poeppigianus* | aerial parts | (Oksuz et al. 1981) |
| **Flv65** | 3´,4´-dihydroxyflavone 5-glucoside | *H. glutinosus* | leaves | (Marambio and Silva 1996) |
| **Flv66** | verbenacoside | *H. glutinosus* | leaves | (Marambio and Silva 1996) |
| *** Flavanones** | | | | |
| **Flv67** | sakuranetin (5,4'-dihydroxy-7-methoxyflavonone) | *H. baylahuen* | resin | (Nuñez-Alarcon et al. 1993) |
|  |  | *H. rigidus* | aerial parts | (Morales et al. 2003) |
|  |  | *H. taeda* | stems | (Marambio and Silva 1989; Faini et al. 2008) |
| **Flv68** | sakuranetin 4´-methyl ether | *H. coquimbensis* | aerial parts | (Maldonado et al. 1993) |
|  |  | *H. remyanus* | resin | (Faini et al. 2011) |
| **Flv69** | persicogenin (3',5-dihydroxy-4',7-dimethoxydihydroflavone) | *H. baylahuen* | resin | (Nuñez-Alarcon et al. 1993) |
|  |  | *H. multifolius* | aerial parts | (Maatooq et al. 2002) |
| **Flv70** | sternbin (3',4',5-trihydroxy-7-methoxydihydroflavone) | *H. baylahuen* | resin | (Nuñez-Alarcon et al. 1993) |
|  |  | *H. multifolius* | aerial parts | (Maatooq et al. 2002) |
|  |  | *H. rigidus* | aerial parts | (Morales et al. 2009) |
|  |  | *H. taeda* | stems | (Marambio and Silva 1989) |
| **Flv71** | eriodictyol | *H. remyanus* | aerial parts | (Zdero et al. 1991a) |
| **Flv72** | eriodictyol 7,3´-dimethyl ether | *H. coquimbensis* | aerial parts | (Maldonado et al. 1993) |
|  |  | *H. taeda* | stems | (Marambio and Silva 1989) |
| **Flv73** | eriodictyol 7,3´,4´-trimethyl ether | *H. taeda* | stems | (Marambio and Silva 1989) |
| **Flv74** | pinostrobin | *H. remyanus* | aerial parts | (Zdero et al. 1991a) |
| *** Flavanonols** | | | | |
| **Flv75** | 7,4´-dimethylaromadendrin | *H. baylahuen* | resin | (Nuñez-Alarcon et al. 1993) |
|  |  | *H. coquimbensis* | aerial parts | (Maldonado et al. 1993) |
|  |  | *H. remyanus* | resin | (Faini et al. 2011) |
| **Flv76** | 7-*O*-methylaromadenrin | *H. baylahuen* | resin | (Nuñez-Alarcon et al. 1993) |
| **Flv77** | 3-*O*-acetyl-7-*O*-aromadendrin | *H. taeda* | stems | (Marambio and Silva 1989) |
| **Flv78** | padmatin | *H. taeda* | stems | (Marambio and Silva 1989) |
| **Flv79** | 3-*O*-acetylpadmatin (3',4',5-trihydroxy-3-acetyl-7-methoxydihydroflavonol) | *H. multifolius* | aerial parts | (Maatooq et al. 2002) |
|  |  | *H. taeda* | stems | (Marambio and Silva 1989) |
| **Flv80** | blumeatin B (3',5-dihydroxy-4',7-dimethoxydihydroflavonol) | *H. multifolius* | aerial parts | (Maatooq et al. 2002) |
| **Flv81** | 7,3′-di-*O*-methyltaxifolin | *H. baylahuen* | resin | (Nuñez-Alarcon et al. 1993) |
|  |  | *H. coquimbensis* | aerial parts | (Maldonado et al. 1993) |
| **Flv82** | dihydromyricetin | *H. baylahuen* | aerial parts | (Schmeda-Hirschmann et al. 2015) |
| **Flv83** | alpinone 3-acetate | *H. remyanus* | aerial parts | (Zdero et al. 1991a) |
| **Coumarins** | | | | |
| *** Simple coumarins** | | | | |
| **Cum1** | esculetin | *H. foliosus* | leaves, aerial parts | (Ulubelen et al. 1982; Urzúa 2004) |
|  |  | *H. multifolius* | aerial parts, leaves, resin | (Chiang et al. 1982; Nuñez-Alarcón and Quiñones 1995; Urzúa et al. 1995b; Torres et al. 2013) |
|  |  | *H. scrobiculatus* | leaves | (Ates et al. 1982) |
| **Cum2** | esculin | *H. multifolius* | leaves | (Torres et al. 2013) |
| **Cum3** | prenyletin | *H. baylahuen* | aerial parts | (Schwenker et al. 1967) |
|  |  | *H. foliosus* | aerial parts | (Urzúa 2004) |
|  |  | *H. multifolius* | aerial parts | (Chiang et al. 1982; Nuñez-Alarcón and Quiñones 1995; Torres et al. 2006) |
|  |  |  | leaves | (Torres et al. 2013) |
|  |  |  | resin | (Urzúa et al. 1995b) |
| **Cum4** | haplopinol | *H. multifolius* | aerial parts | (Chiang et al. 1982; Nuñez-Alarcón and Quiñones 1995; Torres et al. 2006) |
| **Cum5** | 6-deoxyhaplopinol | *H. multifolius* | aerial parts | (Torres et al. 2006) |
| **Cum6** | 6-hydroxy-7-(5´-hydroxy-3´,7´-dimethylocta-2´,6´-dien)-oxycoumarin | *H. multifolius* | resin, aerial parts, leaves | (Torres et al. 2004, 2006, 2013) |
| **Cum7** | 6-hydroxy-7-(7´-hydroxy-3´,7´-dimethylocta-2´,5´-dien)-oxycoumarin | *H. multifolius* | resin, aerial parts, leaves | (Torres et al. 2004, 2006, 2013) |
| **Cum8** | 6-hydroxy-7-[(*E*,*E*)-3´,7´-dimethyl-2´,4´,7´-octatrienyloxy] coumarin | *H. multifolius* | aerial parts | (Torres et al. 2006) |
| **Cum9** | scopoletin | *H. foliosus* | aerial parts | (Urzúa 2004) |
|  |  | *H. velutinus* | stems | (Marambio and Silva 1996) |
| **Cum10** | 7-*O*-prenylscopoletin | *H. deserticola* | aerial parts | (Zdero et al. 1990) |
| **Cum11** | 7-*O*-geranylscopoletin | *H. deserticola* | aerial parts | (Zdero et al. 1990) |
| **Cum12** | scoparone | *H. foliosus* | aerial parts | (Urzúa 2004) |
| **Cum13** | hernianin | *H. multifolius* | aerial parts | (Torres et al. 2006) |
| **Cum14** | umbelliferone | *H. multifolius* | leaves | (Torres et al. 2013) |
| **Cum15** | *O*-prenylumbelliferone | *H. deserticola* | aerial parts | (Zdero et al. 1990) |
|  |  | *H. multifolius* | aerial parts, leaves | (Torres et al. 2006, 2013) |
| **Cum16** | dimeric umbelliferone 3,3-dimethylallyl ether | *H. deserticola* | aerial parts | (Zdero et al. 1990) |
| **Benzoic acid derivatives** | | | | |
| **Ben1** | 4-hydroxybenzoic acid | *H. glutinosus* | stems | (Marambio and Silva 1996) |
| **Ben2** | syringic acid | *H. glutinosus* | stems | (Marambio and Silva 1996) |
| **Ben3** | methyl salicylate | *H. foliosus* | stems | (Villagra et al. 2021) |
| **Cinnamic acid derivatives** | | | | |
| **Cin1** | *trans*-cinnamic acid | *H. foliosus* | resin, aerial parts | (Urzúa et al. 2000; Urzúa 2004) |
|  |  | *H. glutinosus* | stems | (Marambio and Silva 1996) |
| **Cin2** | *cis*-cinnamic acid | *H. foliosus* | aerial parts | (Urzúa 2004) |
| **Cin3** | isobutyl­(*E*)­cinnamate | *H. foliosus* | aerial parts | (Urzúa 2004) |
| **Cin4** | pentyl-(*E*)-cinnamate | *H. foliosus* | aerial parts | (Urzúa 2004) |
| **Cin5** | benzyl­(*E*)­cinnamate | *H. foliosus* | resin, aerial parts | (Urzúa et al. 2000; Urzúa 2004) |
| **Cin6** | 2­phenylethyl­(*E*)­cinnamate | *H. foliosus* | aerial parts | (Urzúa 2004) |
| **Cin7** | 9-*trans*-*p*-coumaroyloxy-α-terpineol | *H. taeda* | resin | (Faini et al. 2007) |
| **Cin8** | 7-*trans*-*p*-coumaroyloxy-taedol | *H. taeda* | resin | (Faini et al. 2007) |
| **Cin9** | caffeic acid | *H. glutinosus* | stems | (Marambio and Silva 1996) |
| **Cin10** | chlorogenic acid | *H. deserticola* | aerial parts | (Schmeda-Hirschmann et al. 2015) |
|  |  | *H. glutinosus* | stems | (Marambio and Silva 1996) |
|  |  | *H. taeda* | aerial parts | (Schmeda-Hirschmann et al. 2015) |
| **Cin11** | 3,4-dicaffeoylquinic acid | *H. deserticola* | aerial parts | (Schmeda-Hirschmann et al. 2015) |
|  |  | *H. taeda* | aerial parts | (Schmeda-Hirschmann et al. 2015) |
| **Cin12** | 3,5-dicaffeoylquinic acid | *H. baylahuen* | aerial parts | (Schmeda-Hirschmann et al. 2015) |
|  |  | *H. deserticola* | aerial parts | (Schmeda-Hirschmann et al. 2015) |
|  |  | *H. multifolius* | aerial parts | (Schmeda-Hirschmann et al. 2015) |
|  |  | *H. rigidus* | aerial parts | (Schmeda-Hirschmann et al. 2015) |
|  |  | *H. taeda* | aerial parts | (Schmeda-Hirschmann et al. 2015) |
